# Idarubicin combats abiraterone and enzalutamide resistance in prostate cells via targeting XPA protein

**DOI:** 10.1038/s41419-022-05490-5

**Published:** 2022-12-12

**Authors:** Ying Zhang, Wei Wei, Changying Li, Siyuan Yan, Shanshan Wang, Shudong Xiao, Chenchen He, Jing Li, Zhi Qi, Benyi Li, Kuo Yang, Changlin Li

**Affiliations:** 1 Institute of Precision Medicine, Jining Medical University, 272067 Jining, China; 2 Center for Experimental Medicine, School of Public Health, Jining Medical University, 272067 Jining, China; 3 Tianjin Institute of Urology, The Second Hospital of Tianjin Medical University, 300211 Tianjin, China; 4 Department of Radiation Oncology, The First Affiliated Hospital, Xi’An Jiaotong University School of Medicine, 710061 Xi’An, China; 5 Department of Histology and Embryology, School of Medicine, Nankai University, 300071 Tianjin, China; 6 Department of Urology, The University of Kansas Medical Center, Kansas City, KS 66160 USA

**Keywords:** Cancer therapy, Translational research

## Abstract

Although second-generation therapies like abiraterone (ABI) and enzalutamide (ENZ) benefit patients with castration-resistant prostate cancer (CRPC), drug resistance frequently occurs, eventually resulting in therapy failure. In this study, we used two libraries, FDA-approved drug library and CRISP/Cas9 knockout (GeCKO) library to screen for drugs that overcome treatment resistance and to identify the potential drug-resistant genes involved in treatment resistance. Our screening results showed that the DNA-damaging agent idarubicin (IDA) overcame abiraterone and enzalutamide resistance in prostate cancer cells. IDA treatment inhibited the DNA repair protein XPA expression in a transcription-independent manner. Consistently, XPA knockout sensitized prostate cancer cells to abiraterone and enzalutamide treatment. In conclusion, IDA combats abiraterone and enzalutamide resistance by reducing XPA protein level in prostate cancer.

## Introduction

Prostate cancer is a leading cause of cancer-related death in Western countries. Androgen deprivation therapy (ADT) remains the standard treatment for prostate cancer patients who cannot undergo prostatectomy. Although ADT initially provides a potent benefit for patients with prostate cancer, their disease state inevitably progresses to castration-resistant prostate cancer (CRPC) within 2–3 years [1]. Abiraterone (ABI) and enzalutamide (ENZ) are second-generation therapies used for patients with CRPC [2–5]. Although both Abiraterone and enzalutamide have demonstrated clinical benefits [3, 4, 6], therapeutic resistance is common, limiting their clinical benefits and resulting in therapy failure after 6 months of treatment [7]. In addition, some patients with CRPC do not respond to abiraterone or enzalutamide treatment, indicating primary resistance [8]. The percentage of abiraterone primary resistance was approximately 10% and 40% in patients with and without chemotherapy, respectively [4, 5]. Similarly, 10% of chemotherapy-naïve patients with CRPC showed enzalutamide primary resistance, whereas 20% of patients pretreated with chemotherapy displayed enzalutamide primary resistance [6, 9]. Therefore, therapeutic resistance is a significant obstacle in CRPC patients. The development of therapeutic strategies to overcome biraterone and enzalutamide resistance is urgently needed [10].

DNA damage response (DDR) is an attractive therapeutic target for CRPC [11–13]. This may be due to the following reasons: (1) most patients with CRPC have DNA damage repair alterations and depend on the remaining DNA repair pathway for survival [14–16]. Therefore, targeting the remaining DNA repair pathways exhibits greater anti-cancer efficacy, leading to prolonged survival of DDR defected patients via the synthetic lethality approach [17]; (2) androgen receptor (AR) signaling pathway remains active in CRPC or ABI/ENZ-resistant patients, which activates the transcriptional expression of several DDR-related proteins in prostate cancers [18, 19]. The human gene XPA encodes a zinc finger protein acting as a DNA damage recognition and repair factor. XPA protein is an essential member of the nucleotide excision repair (NER) complex, a specialized DNA repair mechanism for UV radiation- and chemotherapeutic drugs-induced DNA damage [20–22]. Accumulating evidence showed that XPA protein is closely associated with chemotherapy and radiotherapy resistance in cancer patients [20]. Higher XPA expression is usually associated with poor prognosis in multi-types of human cancers [23], representing a potential target for anti-cancer therapy [20].

Idarubicin (IDA) is an anthracycline that exhibits potent antitumor activity against leukemia, including acute myeloid leukemia and acute promyelocytic leukemia [24]. In addition, clinical studies have indicated that IDA treatment benefits patients with hepatocellular carcinoma [25, 26]. In this study, we identified IDA as a candidate drug to combat ABI/ENZ resistance using a Food and Drug Administration (FDA)-approved drug library. Furthermore, we also determined that XPA is an essential target gene of IDA using liquid chromatography with tandem mass spectrometry (LC-MS/MS) proteomic analysis and the CRISPR-Cas9 knockout (GeCKO) library. Furthermore, in this study, we illustrated how IDA regulates the expression of XPA.

## Results

### Idarubicin combats abiraterone resistance in prostate cancer cells

**Fig. 1:**
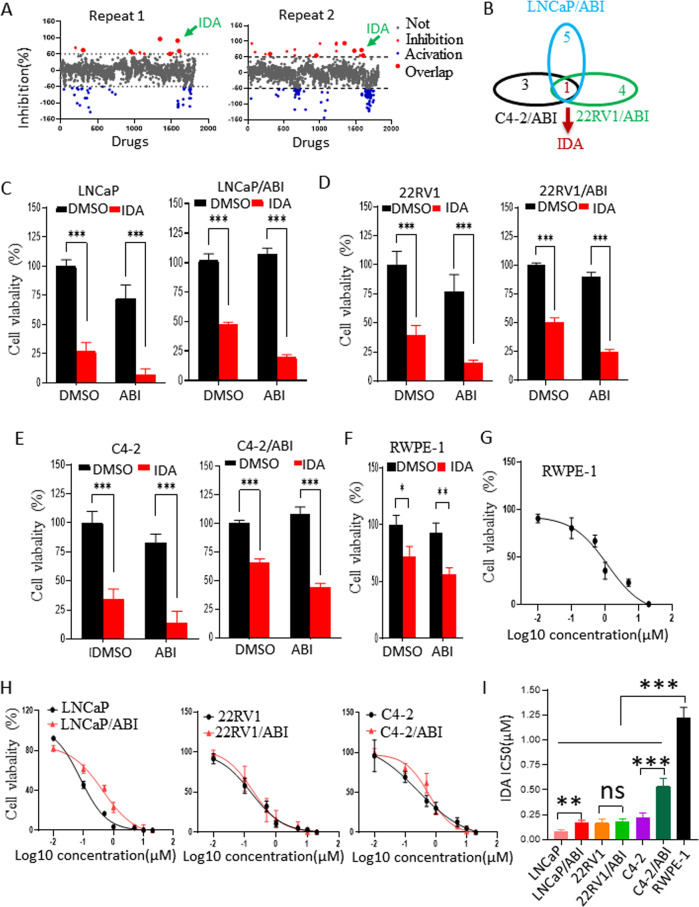
Identification of drugs combats abiraterone resistance using a high-throughput assay. **A** LNCaP/ABI cells were seeded in 96 wells and treated for 24 h with an FDA-approved drug library, including 1815 drugs (*n* = 2). Cell viability was determined by CCK-8 assay. **B** C4-2/ABI and 22RV1/ABI cells were treated with the six drugs (i.g. Otilonium, Idarubicin, Auranofin, Sitafloxacin, Pyrvinium pamoate, and Erdafitinib) for 24 h. Cell viability was determined by CCK-8 assay. The Venn diagram indicates that IDA is a candidate drug for overcoming abiraterone resistance in LNCaP/ABI, C4-2/ABI, and 22RV1/ABI cells. **C**–**F** Abiraterone-resistant cell lines (LNCaP/ABI, 22RV1/ABI, and C4-2/ABI), the parental control cell lines (LNCaP, 22RV1, and C4-2), and RWPE-1 cells were treated with DMSO, IDA, ABI, or a combination of ABI and IDA for 24 h. Cell viability was determined by the CCK-8 assay (*n* = 6). **G**–**I** Abiraterone-resistant cell lines (LNCaP/ABI, 22RV1/ABI, and C4-2/ABI), the parental control cell lines (LNCaP, 22RV1, and C4-2), and RWPE-1 cells were treated with indicated concentration (i.g., 0 μΜ, 0.01 μΜ, 0.1 μΜ, 0.5 μΜ, 1 μΜ, 2 μΜ, 5 μΜ, 10 μΜ, 20 μΜ) in the presence of abiraterone (10 μΜ) for 24 h. Cell viability was determined by CCK-8 assay(*n* = 6). IC_50_ was determined by dose-response curves. Data are presented as means ± s.e.m. The asterisks indicate significant differences (two-way ANOVA, *p < 0.05, **p < 0.01, ***p < 0.01).

To screen for abiraterone-sensitizing drugs, abiraterone-resistant LNCaP cells (LNCaP/ABI) were treated with an FDA-approved drug library containing 1,815 drugs in duplicate (Fig. S1A, B and Supplemental Table S1). Drugs with more than 50% inhibition in duplicates were considered positive hits. Using this criterion, six drugs (i.g. Otilonium, Idarubicin, Auranofin, Sitafloxacin, Pyrvinium pamoate, and Erdafitinib) were identified as the positive hits from LNCaP/ABI screening (Fig. 1A and Supplemental Table S2). We then tested these candidates in two additional abiraterone-resistant prostate cancer cell lines, 22RV1/ABI and C4-2/ABI, as a secondary screening approach. Idarubicin (IDA) was identified as the only drug that inhibited the growth of all abiraterone-resistant cell lines (Fig. 1B). This inhibitory efficacy was confirmed in the parental and abiraterone-resistant subline cells(LNCaP/ABI, 22RV1/ABI, and C4-2/ABI), their parental control cell lines (LNCaP, 22RV1, and C4-2) (Fig. 1C, D, E), and RWPE-1 cells (Fig. 1F). The values for the 50% inhibition of cell growth (IC_50_) were determined by Cell Counting Kit-8 (CCK-8) assay. The highest IC_50_ values were found in prostate epithelial RWPE-1 cells (Fig. 1G, I). The values for IC_50_ were significantly lower in the parental control cells than in abiraterone-resistant cells (Fig. 1H, I).

To further evaluate IDA inhibition of prostate cancer cell growth, the cell death assay was determined by Hoechst/PI staining. IDA treatment largely enhanced cell death in abiraterone-resistant cells, especially when abiraterone was added together with IDA (Fig. S2). These data indicate that Idarubicin (IDA) is the bona-fade candidate drug to combat Abiraterone resistance in castration-resistant prostate cancer cells.

**Fig. 2:**
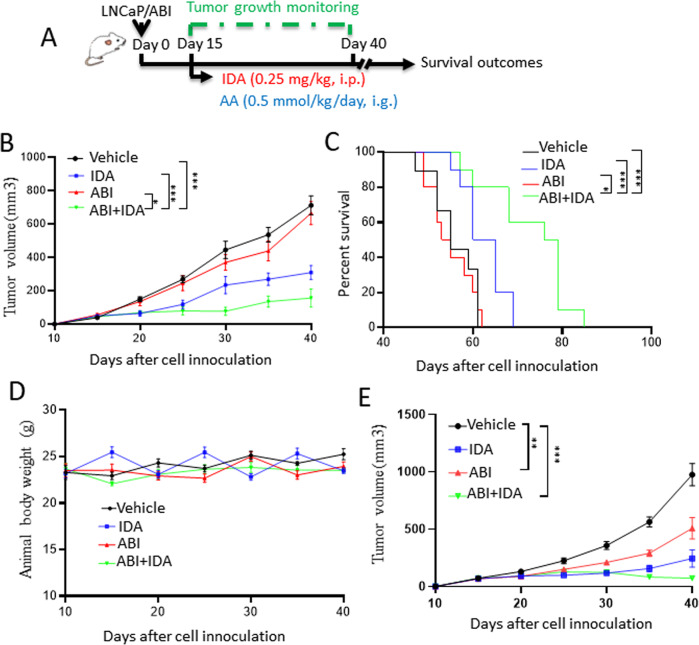
IDA combats abiraterone resistance in vivo. **A**–**D** LNCaP/ABI cells were injected subcutaneously into the flanks of 8-week-old castrated male null mice. When tumor sizes reached about 50 mm^3^, mice were administered with the vehicle (10% DMSO plus 90% corn oil mixture as control, *n* = 8), IDA (0.25 mg/kg, i.p.), AA (0.5 mmol/kg, i.g.), and combination AA(0.5 mmol/kg, i.g.) and IDA(0.25 mg/kg) (*n* = 10, per group) (**A**). Tumor volume (*n* = 10, per group) (**B**), survival (*n* = 10, per group) (**C**), and body weight (*n* = 10, per group) (**D**) were monitored as indicated days. **E** LNCaP cells were injected subcutaneously into the flanks of 8-week-old castrated male null mice. Mice were treated as shown in Fig. 2A. Tumor volume was monitored as indicated days (*n* = 7, per group). Data are presented as means ± s.e.m. The statistical significance of tumor growth (**B**, **E**) and body weight (**D**) were measured by two-way ANOVA analysis. Statistical analysis for overall animal survival (**C**) was performed using and Log-rank (Mantel-Cox) test analysis, respectively. The asterisks indicate significant differences between the indicated groups (*p < 0.05, **p < 0.01, ***p < 0.001).

To examine the in vivo antitumor effect of IDA on abiraterone-resistant prostate cancer, we established subcutaneous xenograft models in nude mice using LNCaP/ABI cells (Fig. 2A). IDA alone or in combination with abiraterone acetate (AA) treatment significantly reduced tumor growth (Fig. 2B) and prolonged mouse survival (Fig. 2C), while AA alone treatment had no obvious effect on tumor growth and animal survival. There were no significant changes in animal body weight among different treatment groups (Fig. 2D). Tissue examination by H&E staining showed no obvious abnormalities from major organs (Fig. S3). We also examined the antitumor effect of IDA on parental LNCaP-derived xenografts. LNCaP-derived mice were treated as described in Fig. 1A. LNCaP-derived xenografts were sensitive to AA treatment, and a combination of AA plus IDA treatments (Fig. 2E). These data indicated that IDA was effective in suppressing xenograft tumor growth without toxicity.

### XPA combats abiraterone resistance in prostate cancer cells

To illustrate the mechanism underlying IDA-induced sensitization of abiraterone resistance, we analyzed the alterations of cellular protein levels in LNCaP/ABI cells after abiraterone plus IDA treatment for 24 h. Mass spectrometry-based protein profiling revealed that 519 proteins were lost in the combination treatment of abiraterone and IDA compared to the abiraterone treatment (Supplemental Table S3). Among these lost proteins, 87 proteins were ubiquitinated proteins, 60 proteins were mitochondrial localized, 55 proteins were zinc finger proteins, 35 proteins were involved in cell cycle modulation, 33 proteins were related to ubiquitination modulation, and 20 proteins were related to DNA damage repair (Supplemental Table S4).

To determine the critical genes involved in the progression of abiraterone resistance, LNCaP/ABI cells were infected with the GeCKO library and injected subcutaneously into the flanks of 8-week-old castrated male nude mice. When tumors were ~50 mm^3^, mice were administered a dosage of AA (0.5 mmol/kg, every day). When tumors reached 1000 mm^3^, they were removed from the mice and subjected to next-generation sequencing (NGS) (Fig. S4A). The curve of the cumulative frequency of sgRNAs after AA treatment shifted toward the left compared with the sgRNA cumulative frequencies before AA treatment (Fig. S4B). CRISPR/Cas9 screening data in this study were obtained from the Sequence Read Archive (SRA) at PRJNA780179. The Venn diagram shows that the expression of 11 genes, including XPA, involved in the development of abiraterone resistance, was decreased by IDA treatment (Fig. S4C and Table S5).

**Fig. 3:**
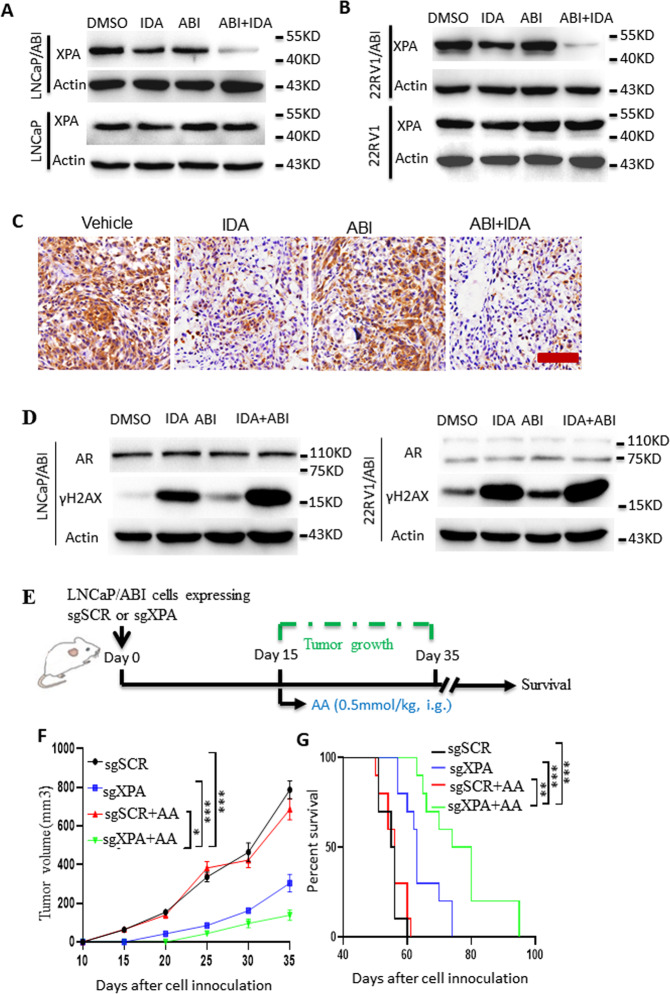
XPA knockout combats the abiraterone resistance. **A**, **B** Abiraterone-resistant cells (LNCaP/ABI and 22RV1/ABI) and their parental control cells were treated with DMSO, ABI (10 μM), IDA (0.25 μM), and combination ABI with IDA. XPA expressions were determined by western blot. **C** XPA expression of tumor tissues from mice administered as shown in Fig. 2A was determined by IPC. Scale bar, 200 μm. **D** LNCaP/ABI and 22RV1/ABI cells were treated with DMSO, ABI (10 μM), IDA (0.25 μM), and combination ABI with IDA. Expressions of γH2AX and AR were determined by western blot. **E**–**G** LNCaP/ABI cells (1 × 10^6^) were infected with lentivirus expressing sgSCR and sgXPA and then injected subcutaneously into the flank of 8-week-old male castrated mull mice. When tumor size reached about 50 mm^3^, mice were administered with vehicle (10% DMSO plus 90% corn oil mixture as control, *n* = 8), AA (0.5 mmol/kg, i.g.), (*n* = 10, per group) (**E**). Tumor volume (**F**) and survival (**G**) were monitored as indicated days. Data are presented as means ± s.e.m. Statistical analysis for tumor growth and overall animal survival were performed using two-way ANOVA analysis (**F**) and Log-rank (Mantel-Cox) test analysis (**G**), respectively. The asterisks indicate significant differences between the indicated groups (*p < 0.05, ***p < 0.001).

XPA is a potential drug target for tumor therapy. Therefore, we hypothesized that IDA overcomes abiraterone resistance by inhibiting XPA expression. To test this hypothesis, we first examined XPA expression after abiraterone treatment with or without IDA addition in resistant cells (LNCaP/ABI and 22RV1/ABI) and their parental control cell lines (LNCaP and 22RV1). IDA treatment largely reduced XPA protein levels in abiraterone-resistant cells but not their parental cells (Fig. 3A, B). Tumors from LNCaP/ABI-bearing mice treated with the combination of IDA and AA consistently displayed lower XPA expression compared with only IDA or DMSO treatment (Fig. 3C).

We next detected the effects of IDA treatment on DNA damage and AR expression in abiraterone-resistant cells. Both IDA alone and in combination with abiraterone treatment induced a drastic increase of γH2X protein, a DNA damage marker (Fig. 3D). AR protein expression did not change after treatment with these two agents (Fig. 3D). These data indicate that IDA inhibited the expression of XPA independent of the AR pathway, and XPA specifically targets IDA in abiraterone-resistant prostate cancer cells.

To verify the critical role of XPA reduction in overcoming abiraterone resistance, we knocked out the XPA gene using the CRISPR/Cas9 approach in abiraterone-resistant cell lines. LNCaP/ABI cells infected with sgSCR or sgXPA lentivirus were used to establish subcutaneous xenografts in nude mice (Fig. 3E). The combination of XPA knockout with AA treatment significantly inhibited tumor growth (Fig. 3F) and prolonged mouse survival (Fig. 3G) compared with the control sgSCR and AA alone treatment. To exclude the off-target effect of sgRNA on XPA, we knocked down XPA using anti-XPA shRNA in abiraterone-resistant cells. Consistent with the results of the XPA knockout using sgRNA for XPA, the knockdown of XPA significantly decreased cell viability in LNCaP/ABI (Fig. S5A, B). These data confirmed that XPA reduction is critical for IDA-induced abiraterone sensitization.

To further verify the role of XPA in abiraterone resistance, we then analyzed clinical profiles of XPA in prostate cancer using the TCGA database. A significantly higher level of XPA mRNA expression was seen in prostate cancer compared to that of control (Fig S6A). Furthermore, we then performed prognostic analysis for abiraterone-treated patients using the dataset (prad_su2c_2019) [27]. Kaplan-Myer survival analysis showed that higher XPA expression was significantly associated with worse overall survival (OS) in abiraterone-treated patients (Fig. S6B). These data suggest that XPA expression is negatively associated with the prognosis of abiraterone-treated patients with CRPC.

### IDA treatment enhances XPA protein degradation through the proteasome pathway

**Fig. 4:**
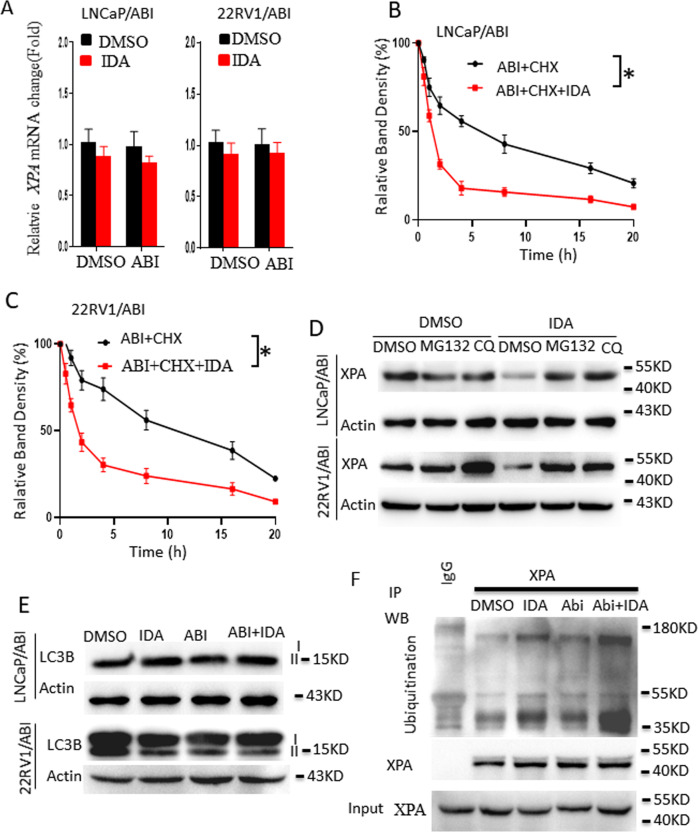
IDA regulates XPA protein levels by enhancing XPA protein degradation. **A** LNCaP/ABI and 22RV1/ABI cells were treated with DMSO, IDA (0.25 μM), ABI (10 μM), and a combination of IDA with ABI for 24 h. XPA mRNA expression was determined by Real-time PCR (*n* = 4). **B**, **C** LNCaP/ABI (**B**) and 22RV1/ABI (**C**) cells were treated with CHX (100 mM) alone or in a combination of IDA (0.25 μM) in the presence of ABI(10 μM) for the indicated periods. Band densities were determined by Image-J software, and the value at time-point 0 was set as 100%. **D** LNCaP/ABI and 22RV1/ABI cells were treated with DMSO, MG132, and CQ(30 μM) with or without IDA(0.25 μM) for 24 h. XPA protein levels were determined by western blot. **E** LNCaP/ABI and 22RV1/ABI cells were treated with DMSO, IDA(0.25 μM), ABI(10 μM), and a combination of IDA with ABI for 24 h. LC3B expressions were determined by western blot assay. **F** LNCaP/ABI cells were treated with DMSO, IDA(0.25 μM), ABI(10 μM), ABI plus IDA for 20 h. Proteasome inhibitor MG132 (1 μM) was included in the assay to protect the ubiquitinated protein from degradation. Ubiquitination of XPA was determined by western blot. Data are presented as means ± s.e.m. Statistical analysis was performed using two-way ANOVA analysis (Two-way ANOVA, *p < 0.05).

To understand how IDA treatment reduces XPA expression, we first analyzed XPA mRNA expression after IDA treatment. IDA alone and in combination with abiraterone did not affect XPA mRNA expression in both LNCaP/ABI and 22RV1/ABI cell lines, indicating a post-transcriptional mechanism (Fig. 4A).

We then examined XPA protein stability after pausing with the protein synthesis inhibitor cycloheximide (CHX) together with or without IDA treatment. After CHX addition, XPA protein levels gradually decreased along with time, while IDA plus CHX treatment significantly enhanced the reduction of XPA protein levels (Fig. 4B, C). These data indicate that IDA treatment promoted XPA protein degradation.

Ubiquitin-proteasome and autophagy are the common processes for protein degradation [28]. We then determined if these two pathways were involved in IDA-induced XPA degradation. LNCaP/ABI and 22RV1/ABI cells were treated with the proteasome inhibitor MG132 and the autophagy inhibitor Chloroquine (CQ) with or without IDA addition. IDA-induced XPA protein reduction was completely blocked by the addition of MG132 or CQ in both cell lines (Fig. 4D). However, IDA treatment did not stimulate the autophagy pathway since LC3B biosynthesis and cleavage were not altered after IDA treatment (Fig. 4E). In contrast, IDA treatment largely enhanced the level of XPA ubiquitination in LNCaP/ABI cells (Fig. 4F). These data strongly suggested that IDA promotes XPA protein degradation mainly via the proteasome pathway but the role of the autophagy pathway. The mechanism underlying XPA protein homeostasis needs further investigation.

### IDA/XPA also combats enzalutamide resistance in prostate cancer cells

**Fig. 5:**
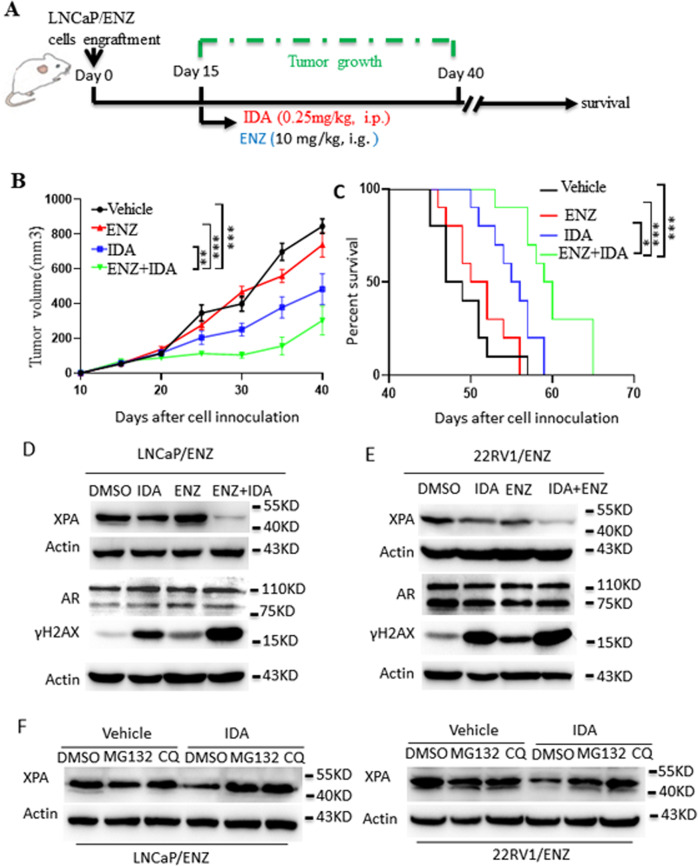
IDA/XPA also combats enzalutamide resistance in prostate cancer cells. **A**–**C** LNCaP/ENZ cells were injected subcutaneously into the flanks of 8-week-old castrated male null mice. When tumor sizes reached ~50 mm^3^, mice were administered with the vehicle (10% DMSO plus 90% corn oil mixture as control, *n* = 10), IDA (0.25 mg/kg, i.p.), EZN (10 mg/kg, i.g.), and combination EZN (10 mg/kg) and IDA (0.25 mg/kg) (**A**). Tumor volume (**B**) and survival (*n* = 10, per group) (**C**) were monitored as indicated days. **D**, **E** LNCaP/ENZ (**D**) and 22RV1/ENZ(E) cells were treated with DMSO, IDA(0.25 μM), ENZ(10 μM), and a combination of ENZ with IDA for 24 h. XPA protein levels, γH2AX, and AR were determined by western blot. F. LNCaP/ENZ and 22RV1/ENZ cells were treated with DMSO, MG132(1 μM), and CQ(30 μM) with or without IDA(0.25 μM) for 24 h. XPA protein levels were determined by western blot. Data are presented as means ± s.e.m. Statistical analysis for tumor growth and overall animal survival were performed using two-way ANOVA analysis (**B**) and Log-rank (Mantel–Cox) test analysis (**C**), respectively. The asterisks indicate significant differences between the indicated groups (*p < 0.05, ***p < 0.001).

Accumulated evidence indicates that abiraterone resistance cross-talks with ENZ resistance under certain circumstances by sharing a similar mechanism (e.g., AR reactivation [29, 30], DNA repair [18], AKT pathway inhibition [31, 32]). Therefore, we investigated whether IDA could overcome enzalutamide resistance. First, enzalutamide-resistant prostate cancer cells (LNCaP/ENZ) were injected into the nude mice. When the tumor size reached 50 mm^3^, the mice were randomly divided into four groups and administered vehicle, IDA, ENZ, or a combination of ENZ and IDA (Fig. 5A). Consistent with the effect of IDA on abiraterone resistance, IDA also or in combination with ENZ significantly inhibited ENZ-resistant tumor growth (Fig. 5B) and increased mouse survival (Fig. 5C). Body weight of mice treated with IDA alone or in combination with ENZ treatment did not display significant differentiation compared to those treated with ENZ alone and vehicle (Fig. S7). We also determined the effect of IDA treatment on parental cells. LNCaP-bearing mice were administered with vehicle, IDA, ENZ, and a combination of IDA and ENZ, respectively. IDA alone and combined with ENZ treatment displayed antitumor activity in LNCaP-bearing mice (Fig. S8). These data indicated that IDA combats enzalutamide resistance.

We further assessed whether IDA affects AR expression and DNA damage in enzalutamide-resistant prostate cancer cells and their parental cell lines. Both IDA alone and in combination with ENZ significantly decreased the expression of XPA and enhanced the expression ofγH2AX compared with that observed in DMSO and ENZ treatment in LNCaP/ENZ (Fig. 5D) and 22RV1/ENZ (Fig. 5E) cells. IDA alone and in combination with ENZ treatment displayed no effect on AR expression in LNCaP/ENZ and 22RV1/ENZ cells (Fig. 5D, E). These data indicate that IDA treatment enhanced DNA damage in ENZ-resistant cells.

We next determined whether ubiquitination and autophagy were involved in the IDA-induced decrease in XPA protein. Similarly, MG132 and CQ reversed the IDA-induced decrease in XPA protein levels in the presence of ENZ in LNCaP/ENZ and 22RV1/ENZ cells (Fig. 5F). These data indicate that IDA decreases XPA protein levels and DNA damage by regulating XPA protein stability.

**Fig. 6:**
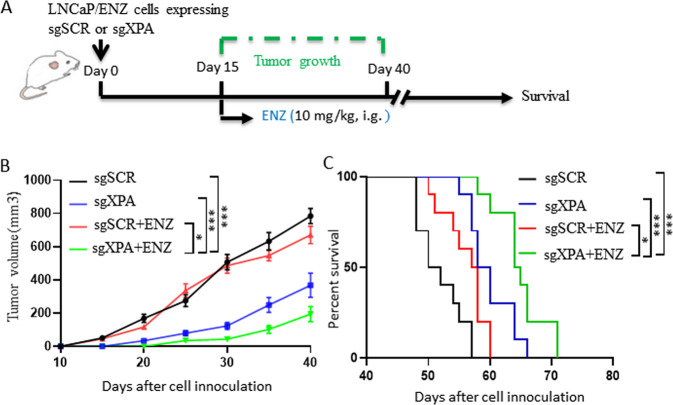
XPA knockout combats the enzalutamide resistance. **A**–**C** LNCaP/ENZ cells (1 × 10^6^) were infected with lentivirus expressing sgSCR and sgXPA and then injected subcutaneously into the flank of 8-week-old male castrated mull mice. When tumor size reached ~50 mm^3^, mice were administered with the vehicle (10% DMSO plus 90% corn oil mixture as control, *n* = 10), ENZ (10 mg/kg, i.g.) (**A**). Tumor volume (*n* = 10) (**B**) and survival (*n* = 10) (**C**) were monitored as indicated days. Data are presented as means ± s.e.m. Statistical analysis for tumor growth and overall animal survival were performed using two-way ANOVA analysis (**B**) and Log-rank (Mantel-Cox) test analysis (**C**), respectively. The asterisks indicate significant differences between the indicated groups (*p < 0.05, ***p < 0.001).

To assay the effect of XPA on ENZ-resistant prostate cancer cells in vivo, LNCaP/ENZ (1 × 10^6^) expressing sgSCR or sgXPA were injected subcutaneously into the flanks of 8-week-old male castrated nude mice (Fig. 6A). The combination of XPA knockout with ENZ treatment significantly inhibited tumor growth (Fig. 6B) and prolonged mouse survival (Fig. 6C) compared to vehicle, sgSCR, or a combination of ENZ and sgSCR. These data indicate that IDA shares a similar mechanism for combating enzalutamide resistance in prostate cancer cells.

## Discussion

In this study, we found that IDA significantly overcame abiraterone and enzalutamide resistance in prostate cancer cells. IDA decreases XPA expression in a transcription-independent manner. XPA is a novel regulator of abiraterone and enzalutamide resistance in prostate cancer. XPA knockout inhibited abiraterone- and enzalutamide-resistant prostate cancer cell growth both in vitro and in vivo. In conclusion, IDA may be a promising candidate drug treatment for patients with abiraterone- and enzalutamide-resistant prostate cancer. Furthermore, XPA is required for IDA-induced antitumor activity in abiraterone- and enzalutamide-resistant prostate cancer cells.

Although abiraterone and ENZ have been widely used for CRPC therapies, their clinical benefits are usually limited to 6 months due to resistance onset [7]. IDA, an anthracycline [33], is a mature clinical drug mainly used in leukemia treatment [24]. Recent studies have shown that IDA exhibits potent antitumor activity against several solid tumors such as hepatocellular carcinoma [25, 26]. In this study, we demonstrated that IDA inhibited the growth of abiraterone- and enzalutamide-resistant prostate cancer cells. Although previous studies have shown some toxic asocial effects with anthracycline treatment [34], we did not find significant side effects on mouse weight and tissues in this study. In addition, IDA treatment exhibited lower cytotoxicity in RWPE-1 normal cells than in prostate cancer cells. Thus, IDA may be a promising treatment option for patients with abiraterone- and enzalutamide -resistant prostate cancer.

The mechanisms underlying the progression of abiraterone and enzalutamide resistance remain unclear. It is widely accepted that AR reactivation [29, 30] and DNA repair [35] are involved in the advancement of resistance to second-generation androgen receptor antagonists. In addition, androgen deprivation regulated the DNA damage response via cross-talk between the AR pathway and DNA repair [18]. In this study, IDA treatment enhanced DNA damage but not AR expression, indicating that IDA treatment regulated DNA repair independent of the AR signaling pathway. Previous studies have demonstrated that combining abiraterone or enzalutamide with the DDR signaling pathway benefits patients with CRPC. Olaparib, a DNA repair inhibitor, in combination with abiraterone, significantly improved the survival of patients with CRPC compared to that observed with abiraterone treatment alone [36]. Similarly, the combination of ENZ with a DNA repair inhibitor effectively prolonged the survival of patients with CRPC compared to that observed with ENZ alone [37]. In this study, we found that IDA, in combination with AA, enhanced DNA damage by decreasing XPA protein levels and overcame the resistance of abiraterone and EZN, which is consistent with previous studies [36, 37].

Previous studies have demonstrated that XPA enhances chemo- and radiotherapy resistance in several cancers [20]. XPA is involved in several cisplatin-resistant cancers such as gastric cancer [38], germ cell tumors [39], prostate cancer [40], and lung cancer [41]. In addition, XPA enhances temozolomide resistance in glioblastoma cells by promoting nucleotide excision repair [42]. Thus, XPA is a potential drug target for tumor therapy. In this study, we found that targeting XPA overcame abiraterone and EZN resistance in prostate cancer cells. XPA is a potential target for abiraterone- and EZN-resistant prostate cancer patients.

Furthermore, XPA expression is controlled by transcriptional regulation [43] and post-translational modification [20]. Previous studies have demonstrated that the circadian clock system [44] and hypoxia-inducible factor 1 alpha (HIF1α) [41] positively regulate the transcriptional expression of XPA. However, non-histone high-mobility group A1 (HMGA1) negatively regulates XPA expression [43]. We found that IDA treatment did not affect XPA mRNA expression in the present study. Therefore, we believe that IDA decreases XPA levels independent of the circadian clock system-, and HIF1a- and HMGA-induced the transcriptional regulation of XPA. XPA is also involved in other biological processes, including transcription [45]. In future studies, we will determine whether XPA mediates the progression of resistance independently of NER.

## Materials and methods

### Cell lines

Human prostate cancer cell lines LNCaP, 22RV1, C4-2 cells, and human prostate epithelial cell line RWPE-1 were obtained from ATCC (Manassas, VA, USA). LNCaP, 22RV1, and C4-2 cells were cultured in RPMI 1640 medium supplemented with 10% fetal bovine serum plus 100 U/ml penicillin/streptomycin and 2 mmol/l l-glutamine (ThermoFisher Scientific, Shanghai, China). RWPE-1 cells were cultured in a Keratinocyte-SFM medium (Invitrogen, CA, USA). All cell lines were authenticated by DNA Nineteen short tandem repeat (STR) at the Institute of Cancer Pathology Research Center at Jining Medical University. LNCaP and 22RV1 cell lines were authenticated on 1/2021, while C4-2 and RWPE-1 cell lines were authenticated on 9/2022. Mycoplasma presence was determined using the MycoSEQ™ Mycoplasma Detection Kit (catalog #4460623; Applied Biosystems).

Abiraterone and enzalutamide-resistant prostate cancer cells were generated as previously described [29]. Briefly, LNCaP, 22RV1, and C4-2 cells were cultured in a steroid-free medium (RPMI + 10% charcoal-stripped serum) for at least six months to establish ADT-resistant prostate cancer cell lines. ADT-resistant prostate cancer cell lines were continuously treated with an increasing concentration of ABI or EZN from 0.5 μM to 10 μM for six months to establish ABI-resistant (i.e., LNCaP/ABI, 22RV1/ABI, and C4-2/ABI) and ENZ-resistant (i.e., LNCaP/ENZ and 22RV1/ENZ) sublines.

### Reagents and antibodies

Antibodies against XPA (ab85914), and anti-ubiquitin (clone number: EPR8830; ab134953) were purchased from Abcam (Cambridge, MA, USA).

Anti-γH2AX (clone number: 20E3; 9718) was obtained from cell signaling technology. Androgen receptor (clone number: PG-21; 06-680), Actin(clone number: AC-15; A5441), and LC3B (ABC432) antibodies were obtained from Sigma-Aldrich (St. Louis, MO). Protein A/G PLUS-Agarose (sc-2003) was obtained from Santa Cruz Biotech (Santa Cruz, CA). HRP-conjugated anti-rabbit IgG (light chain specific, 211-032-171) was ordered from Jackson immunoresearch lab (PA, USA). HRP-conjugated goat anti-mouse IgG and goat anti-rabbit IgG were obtained from Santa Cruz Biotech (Santa Cruz, CA, USA). Abiraterone acetate (AA, HY-75054), Abiraterone (HY-70013), Chloroquine (HY-17589A), MG-132 (Z-Leu-Leu-Leu-al) (HY-13259), Hoechst 33342 (HY-15559), and propidium iodide (PI) (HY-D0815) were obtained from MCE (New Jersey, NJ, USA).

### Cell Counting Kit-8 cell viability assay

Cell viability was determined by CCK-8 Assay, as previously described [46]. Briefly, 1 × 10^5^ cells were seeded in a 96-well culture plate. After 24 h incubation, the cell viability was determined using a CCK-8 (Beyotime, Shanghai, China) following the manufacturer’s instruction. The absorbance at 450 nm was measured by CytExpert (Beckman Coulter, Brea, CA, USA).

### High-throughput screening

The high-throughput screening was performed as described previously [47, 48]. Briefly, for the primary screen, LNCaP-ABI cells (1 × 10^4^/well) were seeded in 96-well plates and treated with an FDA-Approved Drug Library at a final concentration of 1 μM for 24 h. Drug hits were determined by an inhibitory rate above 50% in a CCK-8 assay. LNCaP/ABI, 22RV1/ABI, and C4-2/ABI cells were treated with the drug hits on a secondary screen (Fig. S1A). The first and last rows of the assay plate were treated with the solvent DMSO. The positive (DMSO treatment with CCK-8 reagent) and the negative (DMSO treatment without CCK-8 reagent) were included in the assay (Fig. S1B). The equation to determine cell viability inhibition was as follows: inhibition % = [(*V*_DMSO_−*V*_compound_)/(*V*_DMSO_−*V*_neg_)] × 100, where *V*_compound_ denotes the values in compound-treated wells, *V*_neg_ indicates the value of DMSO treatment without CCK-8 reagent, and V_DMSO_ conveys the values of DMSO treatment with CCK-8 reagent. The drugs with an inhibitory rate over 50% in duplicate replications were regarded as the candidates

### Cell death assay

Cell death was determined by Hoechst/propidium iodide (PI) staining assays as previously described [49, 50]. After drug treatment, cells were incubated in Hoechst (1 μg/ml) and PI(1 μg/ml) for 5 min. The microscopic images were taken using a Nikon Eclipse Ni (Nikon Instruments Inc., Melville, NY).

### CRISPR/Cas9 screen

CRISPR/Cas9 screen was performed as previous studies [51, 52]. Briefly, LNCaP/ABI cells were infected with lentivirus bearing Human GeCKO Lentiviral sgRNA Library v2 (LentiCRISPR) (Addgene plasmid #1000000048). After selection with puromycin, cells were split into two groups. Ones were frozen at −80 °C as control. The others were injected subcutaneously into the flanks of 8-week-old castrated male nude mice. Mice administered with AA (0.5 mmol/kg/day). When tumors reached 1000 mm^3^, Mice were euthanized using CO_2_ inhalation. Tumors were removed from mice. Genomic DNA were extracted form analysis cell pellets using a universal Genomic DNA Kit (Cwbio, Bejing, China). PCR was performed use a 2xGoldStar Best Mix kit according to manufacturer’s protocol (Cwbio, Bejing, China). Primer sequences: sense 5′- AAT GGA CTA TCA TAT GCT TAC CGT AAC TTG AAA GTA TTT CG -3′, and antisense, 5′-AGC CAA TTC CCA CTC CTT TCA AGA CCT AGC-3′. PCR production was extracted from 2% agarose gel using a Gel Extraction Kit (Cwbio, Bejing, China) and subjected to next-generation sequencing (NGS) by a BGISEQ sequencing platform. Data were analyzed using MAGeCKFlute package [53]. Cumulative frequency was determined as previous studies [54].

### Animal xenograft experiments

Tumor cells (1 × 10^6^ cells/100 μl mixed 1:1 with Matrigel) were injected subcutaneously into the flanks of 8-week-old castrated male nude mice (Charles River, Beijing). Once tumor sizes reached about 50 mm^3^, mice were randomized for different treatments (*n* = 7~10), including IDA (0.25 mg/kg, i.p, twice weekly), AA (0.5 mmol/kg, i.g, five days per week) [55], and ENZ (10 mg/kg, i.g, five days per week) [56]. Vehicle (10% DMSO plus 90% corn oil mixture as control). Tumor diameter was blindly measured every five days using a digital caliper. Tumor volumes were calculated using the formula: [(length) × (width)^2^]/2 (V, mm^3^; L, mm; W, mm). Mice were euthanized with CO_2_ inhalation at the end of the experiment. All animal procedures were performed according to the protocol approved by the institutional review committee of Jining Medical University for animal warfare (Ethical permission number: JNMC2021DW006). The animal experiments were carried out in compliance with the ARRIVE guidelines.

### Western blotting and immunohistochemistry assays

Western blotting assay was performed as previously described [57, 58]. Briefly, treated cells were lysed with radioimmunoprecipitation assay (RIPA) buffer (Cell Signaling, Danvers, MA, USA), plugging a 1× protease inhibitor cocktail (Roche, Switzerland). Equal amounts of proteins were then subjected to sodium dodecyl sulfate-polyacrylamide gel electrophoresis (SDS-PAGE) and transferred onto a polyvinylidene fluoride membrane (Merck Millipore, UK). The membranes were incubated with primary antibodies (1:1000) at 4 °C overnight, followed by the peroxidase-conjugated secondary antibody (1:2000) for 1 h at room temperature. Immunoblots were visualized with ECL reagent (Beyotime, Shanghai, China).

Immunohistochemistry (IHC) staining assay was performed using an IHC kit according to the manufacturer’s introduction (BOSTER, Wuhan, China). Briefly, paraffin-embedded tumor sections were deparaffinized and hydrated. Antigen retrieval was conducted in citric acid buffer (pH 6.0). After blocking for 30 min at room temperature, tissue sections were then incubated with primary antibodies at 4 °C overnight, followed by incubation with secondary antibodies for 1 h. Immunosignal visualization was performed with a DAPI-based reagent.

### Real-time PCR

Total RNA was isolated using a Trizol reagent (ThermoFisher Scientific, Shanghai, China). The first strand of cDNA was synthesized using a 5× All-In-One Reverse Transcription Kit (ABM Company, Canada). The reverse transcript products (0.5 μl) were subjected to real-time PCR using an SYBR Green qPCR kit (ABM Company, Canada) using LightCycler® 480 instrument (ROCHE Diagnostic Spa, Mannheim, Germany). 18 S rRNA was used as an endogenous control. The relative expression level was calculated with the 2^[−ΔΔCt]^ approach and expressed as a fold-change. All data were normalized to the levels of 18 S rRNA expression. The primer sequences were designed as follows: 18 s rRNA sense 5′-GAG GAT GAG GTG GAA CGT GT-3′ and antisense 5′-GGA CCT GGC TGT ATT TTC CA-3′; XPA sense 5′-GAG TAT CGA GCG GAA GCG-3′ and antisense 5′-CTC CTG TGT CAA TTA TCT TTG GG-3′.

### Cycloheximide assay

Cycloheximide Assay performed as previously described [59]. Briefly, LNCaP/ABI and 22RV1/ABI cells were treated with CHX (100 mM) alone or in a combination of IDA (0.25 μM) in the presence of ABI for the indicated periods. Band densities were determined by Image-J software, and the value at time-point 0 was set as 100%. The final density data were plotted against time using GraphPad Prism software.

### Plasmid constructs, lentivirus package, and cell infection

Single guide RNA (sgRNA) anti-XPA was cloned into lentiCRISPR V2 (Addgene plasmid #52961) using *Bsm*BI restriction enzyme sites [60]. Small hairpin interfering RNAs (shRNA) against XPA were cloned into PLKO.1-puro vector (Addgene Plasmid #8453) using AgeI/EcoRI restriction enzyme sites [61]. PAX2, pMD2.G (Addgene plasmid #12259-12260), and lenti-CRISPR V2 expressing sgRNA were co-transferred into 293 T cells. After 24 h incubation, the medium was changed to fresh medium and incubated for 24 h. Lentivirus-containing supernatants were collected and infected tumor cells. Stable expression clones were selected with puromycin (1 μg/ml). The sequence of sgRNA anti-XPA was listed as follows: sense, 5′-CAC CGT ACC TGC AGT TAT CAC AAG T-3′ and antisense, 5′-AAA CAC TTG TGA TAA CTG CAG GTA C-3′. The sequence of shRNA anti-XPA was listed as follows: sense, 5′-CAGAGATGCTGATGATAAA-3′ and antisense, 5′-TTTATCATCAGCATCTCTG-3′.

### Ubiquitination detection assay

For ubiquitination detection, LNCaP-ABI cells were treated with DMSO, IDA, ABI, and ABI plus IDA for 20 h. Proteasome inhibitor MG132 (1 μM) was included in the assay to protect the ubiquitinated protein from degradation. Cells were lysed with NP-40 lysis buffer (50 mM Tris pH 7.4, 50 mM NaCl, 1% NP-40, 1x complete protease inhibitors) [62]. Protein lysates were incubated with XPA antibody/G-Agarose complexes for 8 h at 4 °C. The immunoprecipitant elutes were subjected to western blot assay with an anti-ubiquitin antibody followed by HRP-conjugated light chain specific anti-rabbit IgG.

### Protein identification by Mass-Spectroscopy analysis

Total proteins were extracted using the SDT buffer (4% SDS, 100 mM Tris-HCl, 1 mM DTT, pH 7.6). After SDS-PAGE separation, proteins were digested in the gel. The desalted peptides were subjected to LC-MS/MS analysis using a Q Exactive Mass Spectrometer (Thermo Scientific, USA). Protein identification was conducted on the MaxQuant software (version 1.5.3.17) coupled with the UniProt database.

### Data analysis

Data are presented as means ± SEM. Data were derived from at least three independent experiments. One-way variance analysis (ANOVA) was performed to determine the statistical significance among multiple groups. Differences in growth curves and survival were measured by two-way ANOVA and Log-rank (Mantel-Cox) test, respectively. Statistical analyses were performed using GraphPad Prism 8.0.2 software (GraphPad). *P* < 0.05 was considered statistically significant.

### Reporting summary

Further information on research design is available in the Nature Research Reporting Summary linked to this article.

## Supplementary information

Reporting Summary

Anthoes agreements

supplementary Figure legends

Fig S1

Fig S2

Fig S3

Fig S4

Fig S5

Fig S6

Fig S7

Fig S8

Supplemental table legends

Table s1

Table s2

Tab S3 Lost proteins

Tab S4 DAVID cluster

Table s5

Full uncut Western films

## Data Availability

The data generated in this study are available within the article and its supplementary data files. Expression profile data analyzed of the CRISPR/Cas9 screen in this study were obtained from Sequence Read Archive (SRA) at PRJNA780179. Protein Mass-Spectroscopy analyses were obtained from ProteomeXchange (PXD030905).
