# Supplementary material for: Idarubicin combats abiraterone and enzalutamide resistance in prostate cells via targeting XPA protein
Source: Cell Death Dis. 2022 Dec 12;13(12):1034. doi: 10.1038/s41419-022-05490-5 (PMC9744908; doi:10.1038/s41419-022-05490-5)
Supplement: Supplementary file 2 — Anthoes agreements [file 41419_2022_5490_MOESM2_ESM.pdf]

学生邮箱 changlinli@mail.nankai.edu.cn | 设置 | 我的客服 | 自助查询 | 客户端 | English | 退出

首页 | 通讯录 | 应用中心 | 收件箱 | Re:CDD m... ×

收信 | 写信

<< 返回 | 回复 | 回复全部 | 转发 | 删除 | 举报 | 标记为 | 移动到 | 更多

收件箱
红旗邮件
待办邮件
星标联系人邮件
草稿箱
已发送
订阅邮件
其他3个文件夹
邮件标签
邮箱中心
文件中心

Re:CDD manuscript submit

优化阅读 | 精简信息

发件人：张营<zhangying831020@163.com>

收件人：李长林<changlinli@mail.nankai.edu.cn>

时　间：2022年11月21日 23:26 (星期一)

Dr. Changlin Li:

I got it. Thank you!

Ying Zhang

Jining Medical University

At 2022-11-21 21:04:03, "李长林" <changlinli@mail.nankai.edu.cn> wrote:

- 隐藏引用文字 -

> Hi Dr. Ying Zhang：
>
> The manuscript "Idarubicin combats abiraterone and enzalutamide resistance in prostate cells via targeting XPA protein" will be re-submitted to Cell Death & Disease. Co-authors are "*Ying Zhang, Wei Wei, Changying Li, Siyuan Yan, Shanshan Wang, Shudong Xiao, Chenchen He, Jing Li, Zhi Qi, Benyi Li, Kuo Yang, and Changlin Li*", Thank you for what you have done for the manuscript. If you have any question, please feel free to contact me.
>
> Bests,
>
> Changlin Li
>
> Associate professor
>
> Tianjin Institute of Urology
>
> The Second Hospital of Tianjin Medical University

学生邮箱 changlinli@mail.nankai.edu.cn | 设置 | 我的客服 | 自助查询 | 客户端 | English | 退出

首页 | 通讯录 | 应用中心 | 收件箱 | CDD manus... | Re: CDD m...

收信 | 写信

<< 返回 | 回复 | 回复全部 | 转发 | 删除 | 举报 | 标记为 | 移动到 | 更多

收件箱
红旗邮件
待办邮件
星标联系人邮件
草稿箱 (1)
已发送
订阅邮件
其他3个文件夹
邮件标签
邮箱中心
文件中心

Re: CDD manuscript submit

优化阅读 | 精简信息

发件人: wei<124554668@qq.com>

收件人: 李长林<changlinli@mail.nankai.edu.cn>

时　间: 2022年11月22日 09:08 (星期二)

I got it. Thank you!

Wei Wei
Jining Medical University

---Original---

From: "李长林"<changlinli@mail.nankai.edu.cn>
Date: Mon, Nov 21, 2022 22:03 PM
To: "124554668"<124554668@qq.com>;
Subject: CDD manuscript submit

Hi Dr. Wei Wei:

The manuscript "Idarubicin combats abiraterone and enzalutamide resistance in prostate cells via targeting XPA protein" will be re-submitted to Cell Death & Disease. Co-authors are "*Ying Zhang, Wei Wei, Changying Li, Siyuan Yan, Shanshan Wang, Shudong Xiao, Chenchen He, Jing Li, Zhi Qi, Benyi Li, Kuo Yang, and Changlin Li*", Thank you for what you have done for the manuscript. If you have any question, please feel free to contact me.

Bests,

Changlin Li

Associate professor

Tianjin Institute of Urology

The Second Hospital of Tianjin Medical University

学生邮箱 changlinli@mail.nankai.edu.cn | 设置 | 我的客服 | 自助查询 | 客户端 | English | 退出

首页 通讯录 应用中心 收件箱 回复：CDD...

收信 写信

收件箱
红旗邮件
待办邮件
星标联系人邮件
草稿箱
已发送
订阅邮件
其他3个文件夹
邮件标签
邮箱中心
文件中心

<< 返回 | 回复 | 回复全部 | 转发 | 删除 | 举报 | 标记为 | 移动到 | 更多

回复：CDD manuscript submit

发件人：cli_cvrl@tmu.edu.cn<cli_cvrl@tmu.edu.cn>

收件人：李长林<changlinli@mail.nankai.edu.cn>

时 间：2022年11月16日 08:40 (星期三)

优化阅读 | 精简信息

OK. Thanks a lot.

发自我的手机

-------- 原始邮件 --------
发件人：李长林 <changlinli@mail.nankai.edu.cn>
日期：2022年11月15日周二 下午3:25
收件人：cli_cvrl@tmu.edu.cn
主 题：CDD manuscript submit

Hi Dr. Changying Li：

The manuscript "Idarubicin combats abiraterone and enzalutamide resistance in prostate cells via targeting XPA protein" will be re-submitted to Cell Death & Disease. Co-authors are "*Ying Zhang, Wei Wei, Changying Li, Siyuan Yan, Shanshan Wang, Shudong Xiao, Chenchen He, Jing Li, Zhi Qi, Benyi Li, Kuo Yang, and Changlin Li*", Thank you for what you have done for the manuscript. If you have any question, please fell free to contact me.

Bests,

Changlin Li

Associate professor

Tianjin Institute of Urology

The Second Hospital of Tianjin Medical University

学生邮箱 changlinli@mail.nankai.edu.cn | 设置 | 我的客服 | 自助查询 | 客户端 | English | 退出

首页 | 通讯录 | 应用中心 | 收件箱 | Re: CDD m...

收信 | 写信

<< 返回 | 回复 | 回复全部 | 转发 | 删除 | 举报 | 标记为 | 移动到 | 更多

Re: CDD manuscript submit

发件人：yansy<yansy@mail.jnmc.edu.cn>

收件人：李长林<changlinli@mail.nankai.edu.cn>

时 间：2022年11月21日 22:19 (星期一)

Dr. Changlin Li,

I got it. Thank you and best wishes!

Siyuan Yan

Jining Medical University

11/21/2022

——原始邮件——

发件人："李长林" <changlinli@mail.nankai.edu.cn>

发送时间：2022-11-21 21:06:14 (星期一)

收件人： yansy@mail.jnmc.edu.cn

抄送：

主题： CDD manuscript submit

Hi Dr. Siyuan Yan:

The manuscript "Idarubicin combats abiraterone and enzalutamide resistance in prostate cells via targeting XPA protein" will be re-submitted to Cell Death & Disease. Co-authors are "*Ying Zhang, Wei Wei, Changying Li, Siyuan Yan, Shanshan Wang, Shudong Xiao, Chenchen He, Jing Li, Zhi Qi, Benyi Li, Kuo Yang, and Changlin Li*", Thank you for what you have done for the manuscript. If you have any question, please fell free to contact me.

Bests,

Changlin Li

Associate professor

学生邮箱 changlinli@mail.nankai.edu.cn | 设置 | 我的客服 | 自助查询 | 客户端 | English | 退出

首页 通讯录 应用中心 收件箱 回复：CDD...

收信 写信

<< 返回 回复 回复全部 转发 删除 举报 标记为 移动到 更多

收件箱
红旗邮件
待办邮件
星标联系人邮件
草稿箱
已发送
订阅邮件
其他3个文件夹
邮件标签
邮箱中心
文件中心

回复：CDD manuscript submit

优化阅读 | 精简信息

发件人：shuijing9020<shuijing9020@aliyun.com>

收件人：李长林<changlinli@mail.nankai.edu.cn>

时 间：2022年11月21日 22:13 (星期一)

Dr. Changlin Li:

I got it. Thank you!

Best regards

Shanshan Wang
Jining Medical University

发件人：李长林 <changlinli@mail.nankai.edu.cn>
发送时间：2022年11月21日(星期一) 08:38
收件人：shuijing9020 <shuijing9020@aliyun.com>
主 题：CDD manuscript submit

Hi Dr. Shanshan Wang：

The manuscript "Idarubicin combats abiraterone and enzalutamide resistance in prostate cells via targeting XPA protein" will be re-submitted to Cell Death & Disease. Co-authors are "*Ying Zhang, Wei Wei, Changying Li, Siyuan Yan, Shanshan Wang, Shudong Xiao, Chenchen He, Jing Li, Zhi Qi, Benyi Li, Kuo Yang, and Changlin Li*", Thank you for what you have done for the manuscript. If you have any question, please feel free to contact me.

Bests,

Changlin Li

Associate professor

Tianjin Institute of Urology

The Second Hospital of Tianjin Medical University

学生邮箱 changlinli@mail.nankai.edu.cn | 设置 | 我的客服 | 自助查询 | 客户端 | English | 退出

首页 通讯录 应用中心 收件箱 Re: CDD m...

收信 写信

<< 返回 回复 回复全部 转发 删除 举报 标记为 移动到 更多

收件箱
红旗邮件
待办邮件
星标联系人邮件
草稿箱
已发送
订阅邮件
其他3个文件夹
邮件标签
邮箱中心
文件中心

Re: CDD manuscript submit

优化阅读 精简信息

发件人: 舍予<1410749937@qq.com>

收件人: 李长林<changlinli@mail.nankai.edu.cn>

时 间: 2022年11月21日 21:32 (星期一)

Dr. Changlin Li:

I got it. Thank you!

Shudong Xiao

---Original---

From: "李长林"<changlinli@mail.nankai.edu.cn>
Date: Mon, Nov 21, 2022 21:25 PM
To: "1410749937"<1410749937@qq.com>;
Subject: CDD manuscript submit

Hi Dr. Shudong Xiao:

The manuscript "Idarubicin combats abiraterone and enzalutamide resistance in prostate cells via targeting XPA protein" will be re-submitted to Cell Death & Disease. Co-authors are "*Ying Zhang, Wei Wei, Changying Li, Siyuan Yan, Shanshan Wang, Shudong Xiao, Chenchen He, Jing Li, Zhi Qi, Benyi Li, Kuo Yang, and Changlin Li*", Thank you for what you have done for the manuscript. If you have any question, please feel free to contact me.

Bests,

Changlin Li

Associate professor

Tianjin Institute of Urology

The Second Hospital of Tianjin Medical University

生邮箱 changlinli@mail.nankai.edu.cn | 设置 | 我的客服 | 自助查询 | 客户端 | English | 退出

页 通讯录 应用中心 收件箱 Re: CDD m... ×

写信 << 返回 回复 回复全部 转发 删除 举报 标记为 移动到 更多

件
件
系人邮件
件夹

Re: CDD manuscript submit

优化阅读 | 精简信息

发件人: CHEN一叶扁舟<382120433@qq.com>

收件人: 李长林<changlinli@mail.nankai.edu.cn>

时 间: 2022年11月15日 21:04 (星期二)

Dear Changlin Li:

Thank you for your careful instruction!

Chenchen He

Department of Radiation Oncology,

The First Affiliated Hospital, Xi'an Jiaotong University, Xi'an, China.

发自我的iPhone

------------------ Original ------------------

From: 李长林 <changlinli@mail.nankai.edu.cn>
Date: Tue,Nov 15,2022 8:50 PM
To: hechenchen.19 <hechenchen.19@stu.xjtu.edu.cn>
Cc: 382120433 <382120433@qq.com>
Subject: Re: CDD manuscript submit

Hi Dr. Chenchen He:

The manuscript "Idarubicin combats abiraterone and enzalutamide resistance in prostate cells via targeting XPA protein" will be re-submitted to Cell Death & Disease. Co-authors are "*Ying Zhang, Wei Wei, Changying Li, Siyuan Yan, Shanshan Wang, Shudong Xiao, Chenchen He, Jing Li, Zhi Qi, Benyi Li, Kuo Yang, and Changlin Li*", Thank you for what you have done for the manuscript. If you have any question, please fell free to contact me.

Bests,

Changlin Li

Associate professor

学生邮箱 changlinli@mail.nankai.edu.cn | 设置 | 我的客服 | 自助查询 | 客户端 | English | 退出

首页 通讯录 应用中心 收件箱 Re:CDD m...

收信 写信

收件箱
红旗邮件
待办邮件
星标联系人邮件
草稿箱
已发送
订阅邮件
其他3个文件夹
邮件标签
邮箱中心
文件中心

返回 回复 回复全部 转发 删除 举报 标记为 移动到 更多

Re:CDD manuscript submit

优化阅读 | 精简信息

发件人：李静<stellarli@nankai.edu.cn>

收件人：李长林<changlinli@mail.nankai.edu.cn>

时 间：2022年11月21日 21:26 (星期一)

Got it. Thank you^_^

Jing Li

发件人："李长林" <changlinli@mail.nankai.edu.cn>

发送日期：2022-11-21 21:08:30

收件人：stellarli@nankai.edu.cn

主题：CDD manuscript submit

- 隐藏引用文字 -

Hi Dr. JIng Li：

The manuscript "Idarubicin combats abiraterone and enzalutamide resistance in prostate cells via targeting XPA protein" will be re-submitted to Cell Death & Disease. Co-authors are "*Ying Zhang, Wei Wei, Changying Li, Siyuan Yan, Shanshan Wang, Shudong Xiao, Chenchen He, Jing Li, Zhi Qi, Benyi Li, Kuo Yang, and Changlin Li*", Thank you for what you have done for the manuscript. If you have any question, please fell free to contact me.

Bests,

Changlin Li

Associate professor

Tianjin Institute of Urology

The Second Hospital of Tianjin Medical University

学生邮箱 changlinli@mail.nankai.edu.cn | 设置 | 我的客服 | 自助查询 | 客户端 | English | 退出

首页 | 通讯录 | 应用中心 | 收件箱 | Re:CDD m...

收信 | 写信

<< 返回 | 回复 | 回复全部 | 转发 | 删除 | 举报 | 标记为 | 移动到 | 更多

收件箱
红旗邮件
待办邮件
星标联系人邮件
草稿箱
已发送
订阅邮件
其他3个文件夹
邮件标签
邮箱中心
文件中心

Re:CDD manuscript submit

优化阅读 | 精简信息

发件人：漆智<qizhi@nankai.edu.cn>

收件人：李长林<changlinli@mail.nankai.edu.cn>

时　间：2022年11月21日 22:22 (星期一)

Dear Prof. Li

Thank you for your e-mail. I agree to all the changes in revised manuscript, including the changes for authors order.

Best regards

Zhi Qi MD. PhD
Professor, Doctoral Supervisor
School of Medicine, Nankai University
Add: 94 Weijin Road, Tianjin, China, 300071
Tel.: 86-22-23509877
Fax.: 86-22-23502554
E-mail: qizhi@nankai.edu.cn

发件人："李长林" <changlinli@mail.nankai.edu.cn>
发送日期：2022-11-21 21:09:27
收件人：qizhi@nankai.edu.cn
主题：CDD manuscript submit

- 显示引用文字 -

快捷回复给：漆智

学生邮箱 changlinli@mail.nankai.edu.cn | 设置 | 我的客服 | 自助查询 | 客户端 | English | 退出

首页 通讯录 应用中心 收件箱 Re: CDD m... ×

收信 写信

收件箱
红旗邮件
待办邮件
星标联系人邮件
草稿箱
已发送
订阅邮件
其他3个文件夹
邮件标签
邮箱中心
文件中心

返回 回复 回复全部 转发 删除 举报 标记为 移动到 更多

Re: CDD manuscript submit

发件人: Benyi Li<BLI@kumc.edu>
收件人: 李长林<changlinli@mail.nankai.edu.cn>
时 间: 2022年11月15日 19:53 (星期二)

优化阅读 精简信息

Okay.

Get Outlook for Android

From: 李长林 <changlinli@mail.nankai.edu.cn>
Sent: Tuesday, November 15, 2022 1:36:22 AM
To: Benyi Li <BLI@kumc.edu>
Subject: CDD manuscript submit

Dear Dr. Benyi Li:

The manuscript "Idarubicin combats abiraterone and enzalutamide resistance in prostate cells via targeting XPA protein" will be re-submitted to Cell Death & Disease. Co-authors are "*Ying Zhang, Wei Wei, Changying Li, Siyuan Yan, Shanshan Wang, Shudong Xiao, Chenchen He, Jing Li, Zhi Qi, Benyi Li, Kuo Yang, and Changlin Li*", Thank you for what you have done for the manuscript. If you have any question, please fell free to contact me.

Bests,

Changlin Li

Associate professor

Tianjin Institute of Urology

The Second Hospital of Tianjin Medical University

快捷回复给: Benyi Li

学生邮箱 changlinli@mail.nankai.edu.cn | 设置 | 我的客服 | 自助查询 | 客户端 | English | 退出

首页 | 通讯录 | 应用中心 | 收件箱 | Re:CDD m...

收信 | 写信

收件箱
红旗邮件
待办邮件
星标联系人邮件
草稿箱
已发送
订阅邮件
其他3个文件夹
邮件标签
邮箱中心
文件中心

返回 | 回复 | 回复全部 | 转发 | 删除 | 举报 | 标记为 | 移动到 | 更多

Re:CDD manuscript submit

发件人：杨阔<ykuoster@126.com>

收件人：李长林<changlinli@mail.nankai.edu.cn>

时 间：2022年11月22日 08:16 (星期二)

Hi. Changlin:

I got it. Thank you!

Kuo Yang

At 2022-11-21 21:52:25, "李长林" <changlinli@mail.nankai.edu.cn> wrote:

- 隐藏引用文字 -

Hi Dr. Kuo Yang：

The manuscript "Idarubicin combats abiraterone and enzalutamide resistance in prostate cells via targeting XPA protein" will be re-submitted to Cell Death & Disease. Co-authors are "*Ying Zhang, Wei Wei, Changying Li, Siyuan Yan, Shanshan Wang, Shudong Xiao, Chenchen He, Jing Li, Zhi Qi, Benyi Li, Kuo Yang, and Changlin Li*", Thank you for what you have done for the manuscript. If you have any question, please feel free to contact me.

Bests,

Changlin Li

Associate professor

Tianjin Institute of Urology

The Second Hospital of Tianjin Medical University
