# Supplementary material for: Idarubicin combats abiraterone and enzalutamide resistance in prostate cells via targeting XPA protein
Source: Cell Death Dis. 2022 Dec 12;13(12):1034. doi: 10.1038/s41419-022-05490-5 (PMC9744908; doi:10.1038/s41419-022-05490-5)
Supplement: Supplementary file 3 — supplementary Figure legends [file 41419_2022_5490_MOESM3_ESM.docx]

Figure S1. HTS process

A. Schematic of the HTS strategy. For the primary screen, LNCaP/ABI cells (1x10^4^/well) were seeded in 96-well plates and treated with an FDA-Approved Drug Library at a final concentration of 1 μM for 24 h(n=2). LNCaP/ABI, 22RV1/ABI, and C4-2/ABI cells were treated with the hits drugs on the secondary screen.

B. Layout of 96-well plate.

Fig. S2 IDA treatment induced cell death in ABI-resistant prostate cancer cells

LNCaP/ABI and 22RV1/ABI cells were treated with DMSO, IDA, ABI, or a combination of ABI and IDA for 24 h. Cell death was determined by Hoechst/PI staining assays. The microscopic images were taken using a Nikon Eclipse Ni.

 Fig. S3 H&E stainning of mice tissues

Mice were treated as shown in Fig.2A. H&E stainning of mice tissues (n=5 animals for each group). Bar, 200μm.

Fig. S4 GeCKO library scanning

A. LNCaP/ABI cells were infected with GeCKO library and injected subcutaneously into the 8-week-old castrated male nude mice flanks. When tumors were approximately 50 mm^3^, mice were administered with AA (0.5 mmol/kg/day). When tumor size was about 1000 mm^3^, tumors were removed from mice and subjected to deep sequencing.

B. The cumulative frequencies of sgRNAs were determined by next-generation sequencing (NGS).

**C**. Intersection of hits from MS and GeCKO datasets. Left circle: IDA targeting genes; Right circle: LNCaP/ABI cells were infected with lentivirus expressing GeCKO library. MAGeCKFlute determined hit genes. Middle part: the intersection of the two datasets.

Fig. S5 XPA knockdown inhibits cell viability of abiraterone-resistant prostate cells

LNCaP/ABI cells stably infected with lentiviruses harboring control vector, shRNA constructs were treated with DMSO, ABI, IDA, and in combination with ABI for 24h.

A. Cell viability was were determined by CCK-8 assay (*n*=5).

B. The efficiency of *XPA* knockdown was determined by western blot assay. Actin served as the protein loading control.

Fig. S6 Clinical profiles of XPA in prostate cancer

A, B. XPA expression was determined by the TCGA database in prostate cancer samples (n = 498) and normal mammary tissues (GTEx, n = 245).

B. The association of XPA with OS was analyzed in prad_su2c_2019 dataset. Statistical analysis for XPA mRNA expression was determined by Student’s *t*-test (A). Statistical analysis for overall animal survival (B) was performed using and Log-rank (Mantel-Cox) test analysis.

Fig. S7 IDA treatment did not affect the body weight of LNCaP/ENZ-bearing mice

LNCaP/ENZ-bearing mice were treated as described in Fig. 5A. Body weight of mice was monitored as indicated days (*n* = 10, per group).

Data are presented as means ± s.e.m. Statistical analysis was performed using two-way ANOVA analysis.

Fig. S8 IDA exhibits antitumor activity in ENZ-sensitive prostate cancer cells

LNCaP cells were injected subcutaneously into the flanks of 8-week-old castrated male null mice. When tumor sizes reached about 50 mm^3^, mice were administered with IDA (0.25 mg/kg), ENZ (10mg/kg, i.g.), and combination ENZ (10mg/kg, i.g.) and IDA(0.25 mg/kg, i.p.). Tumor volume was monitored as indicated days (*n*=7, per group).

Data are presented as means ± s.e.m. Statistical analysis was performed using two-way ANOVA analysis (*** *p* < 0.001).
