# Supplementary figures and images for: Idarubicin combats abiraterone and enzalutamide resistance in prostate cells via targeting XPA protein
Source: Cell Death Dis. 2022 Dec 12;13(12):1034. doi: 10.1038/s41419-022-05490-5 (PMC9744908; doi:10.1038/s41419-022-05490-5)

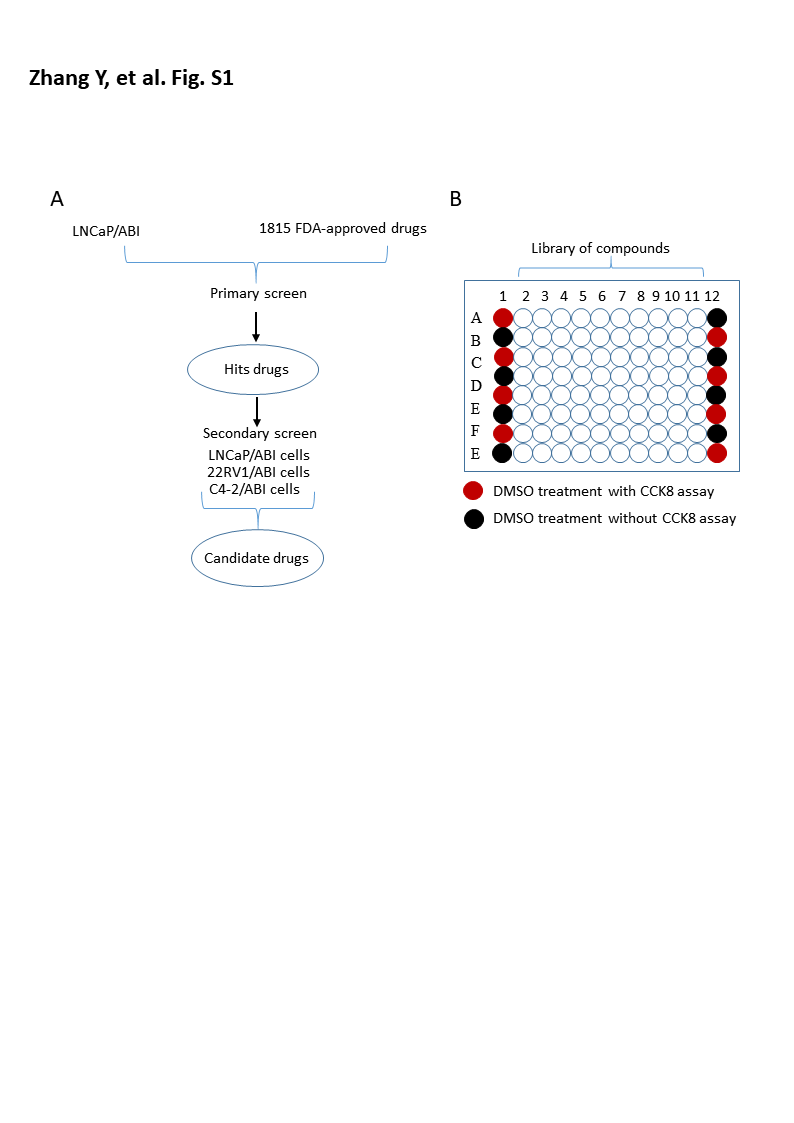

Supplement: Supplementary file 4 — Fig S1 [file 41419_2022_5490_MOESM4_ESM.tif]

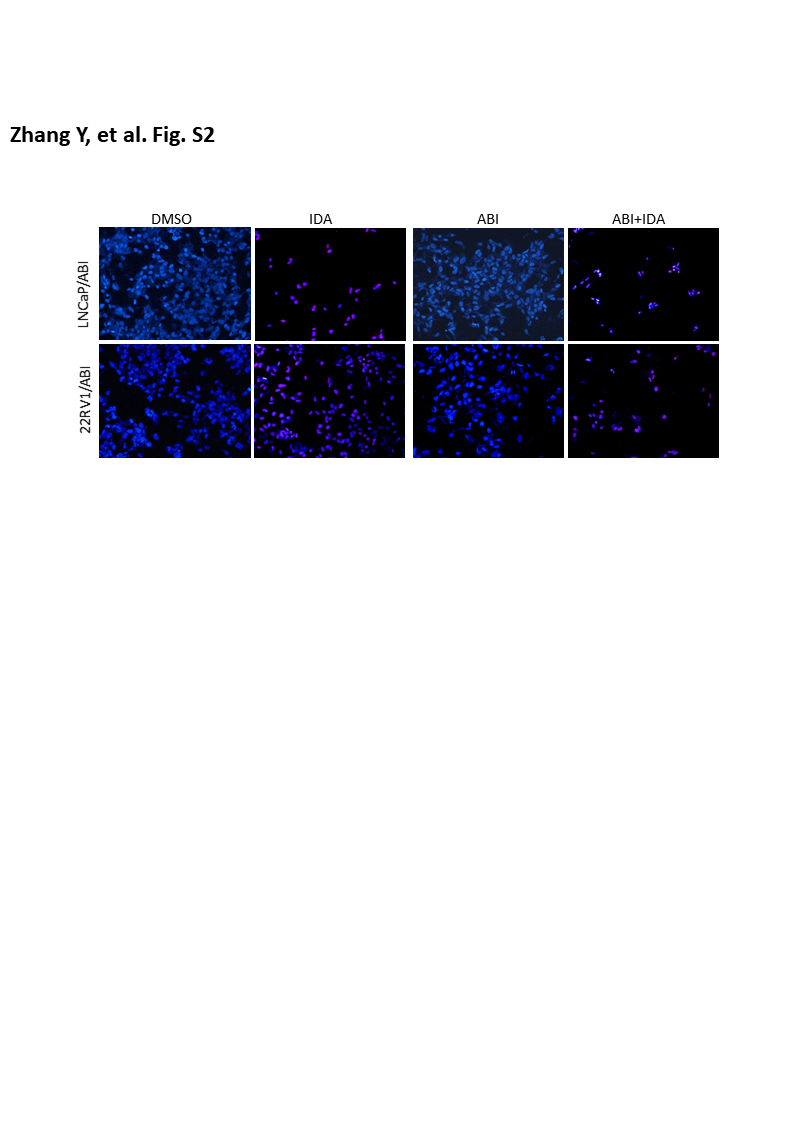

Supplement: Supplementary file 5 — Fig S2 [file 41419_2022_5490_MOESM5_ESM.tif]

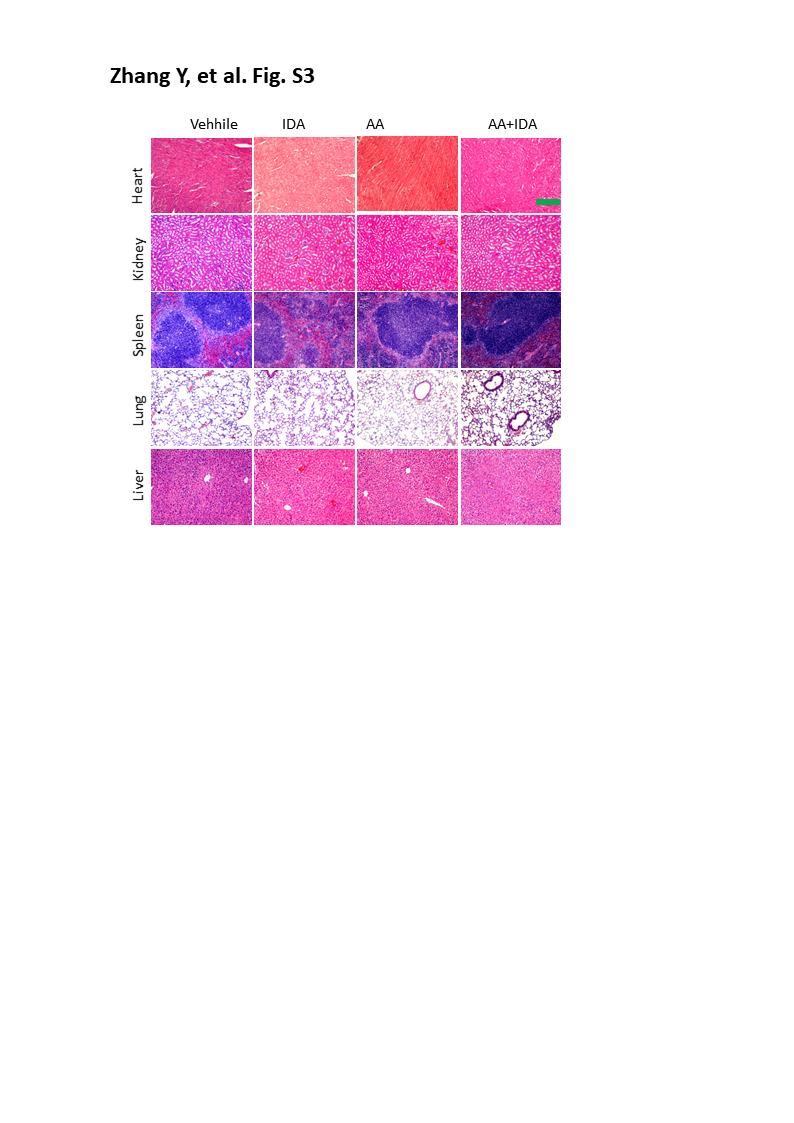

Supplement: Supplementary file 6 — Fig S3 [file 41419_2022_5490_MOESM6_ESM.tif]

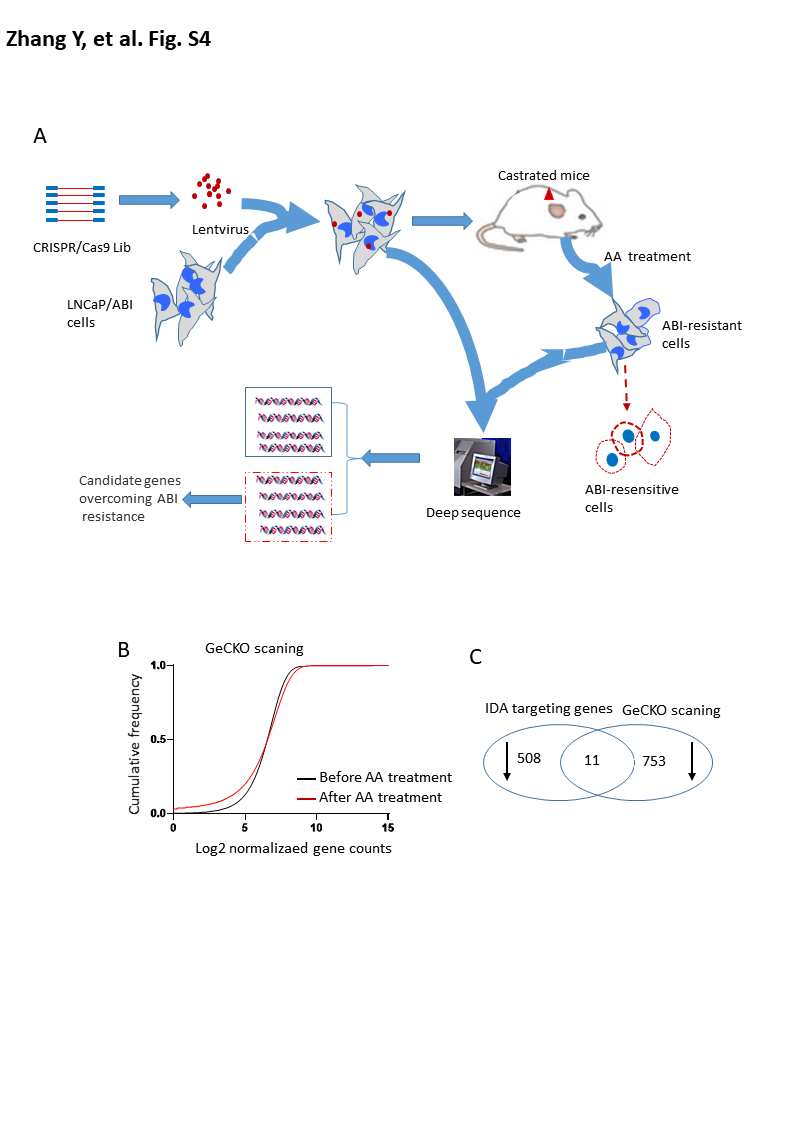

Supplement: Supplementary file 7 — Fig S4 [file 41419_2022_5490_MOESM7_ESM.tif]

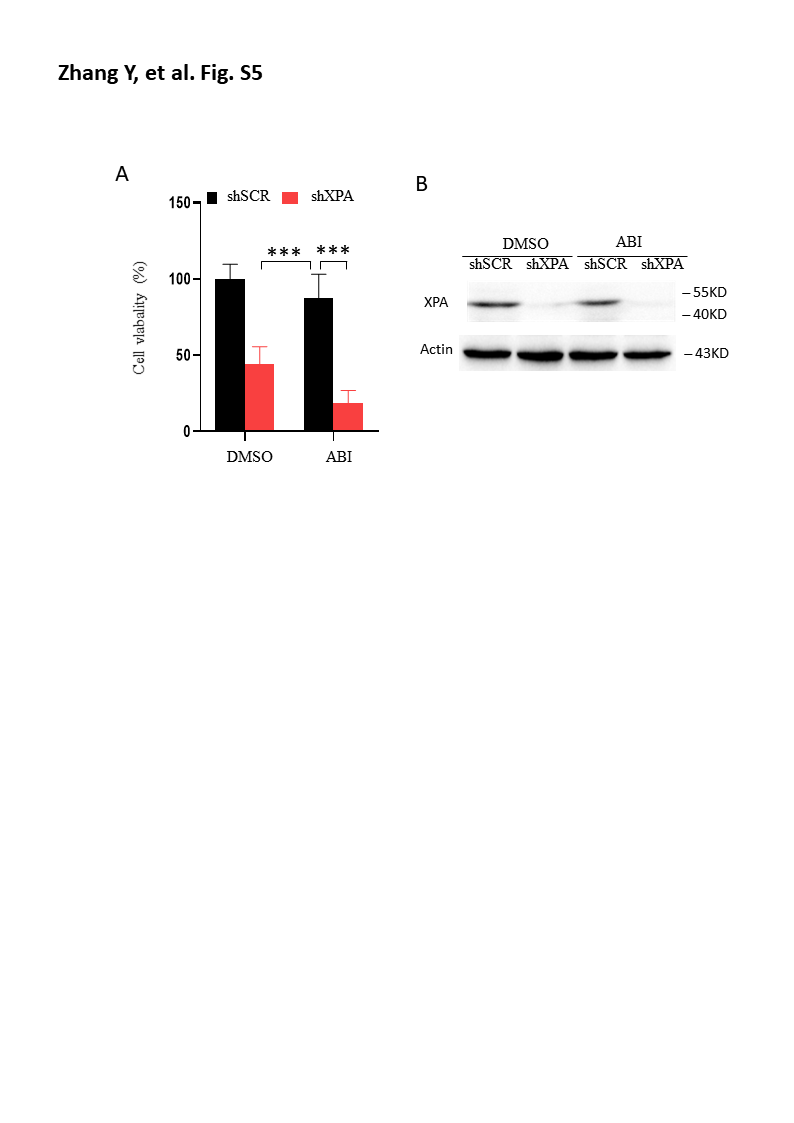

Supplement: Supplementary file 8 — Fig S5 [file 41419_2022_5490_MOESM8_ESM.tif]

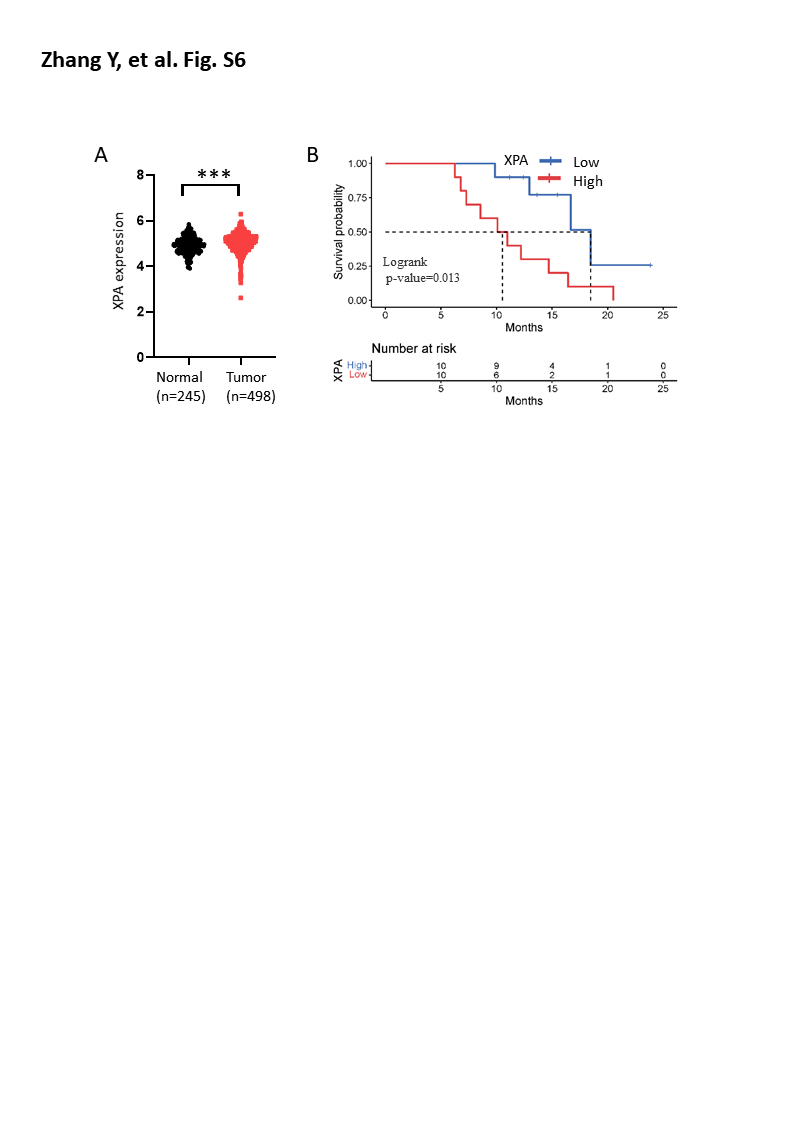

Supplement: Supplementary file 9 — Fig S6 [file 41419_2022_5490_MOESM9_ESM.tif]

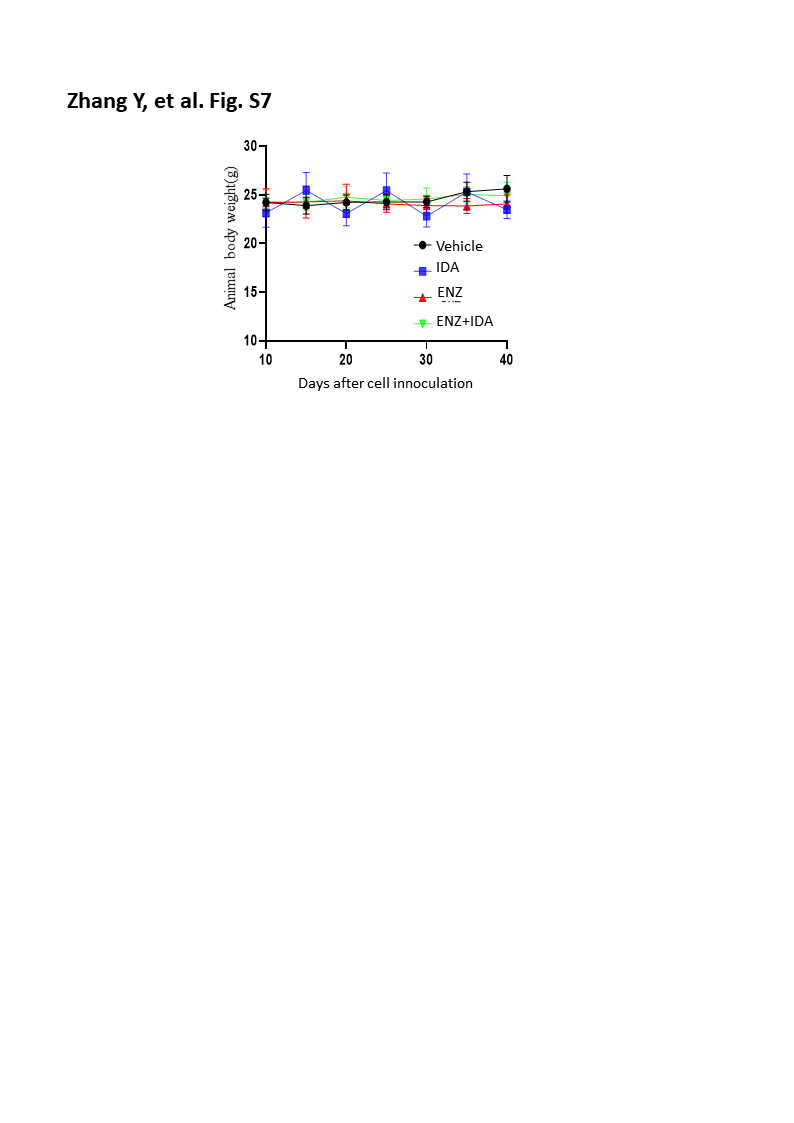

Supplement: Supplementary file 10 — Fig S7 [file 41419_2022_5490_MOESM10_ESM.tif]

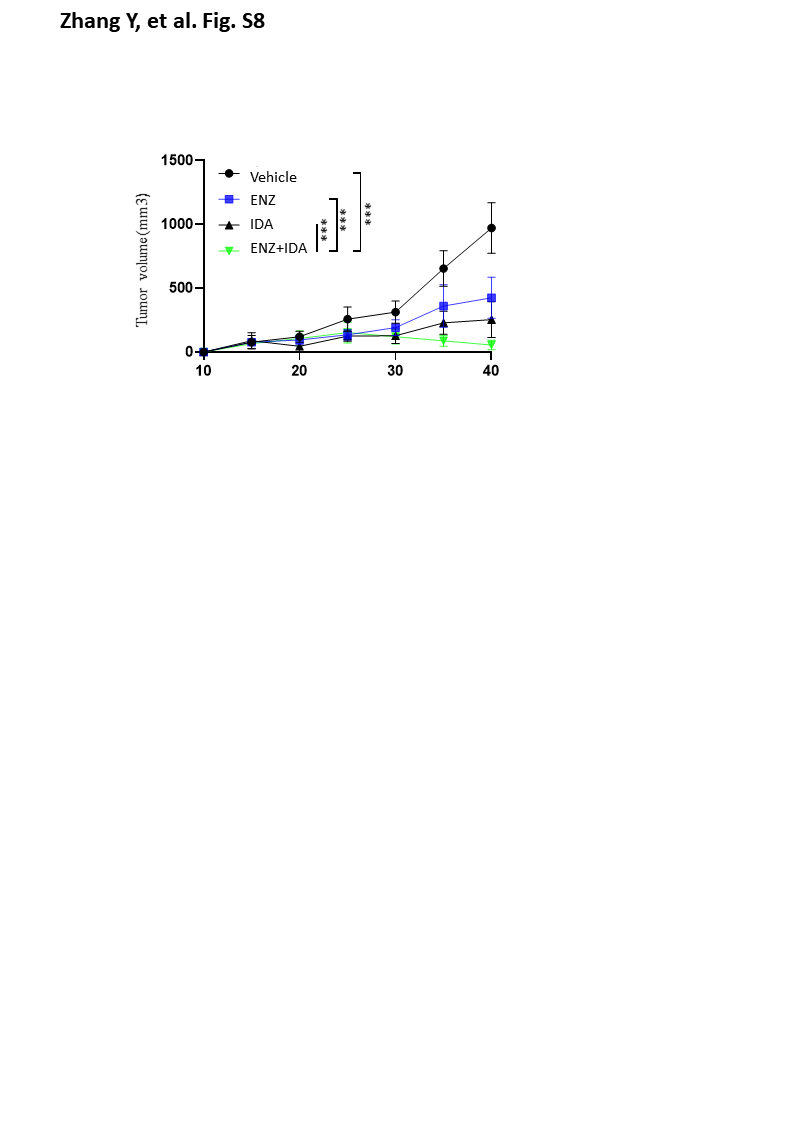

Supplement: Supplementary file 11 — Fig S8 [file 41419_2022_5490_MOESM11_ESM.tif]

Fig. 3A

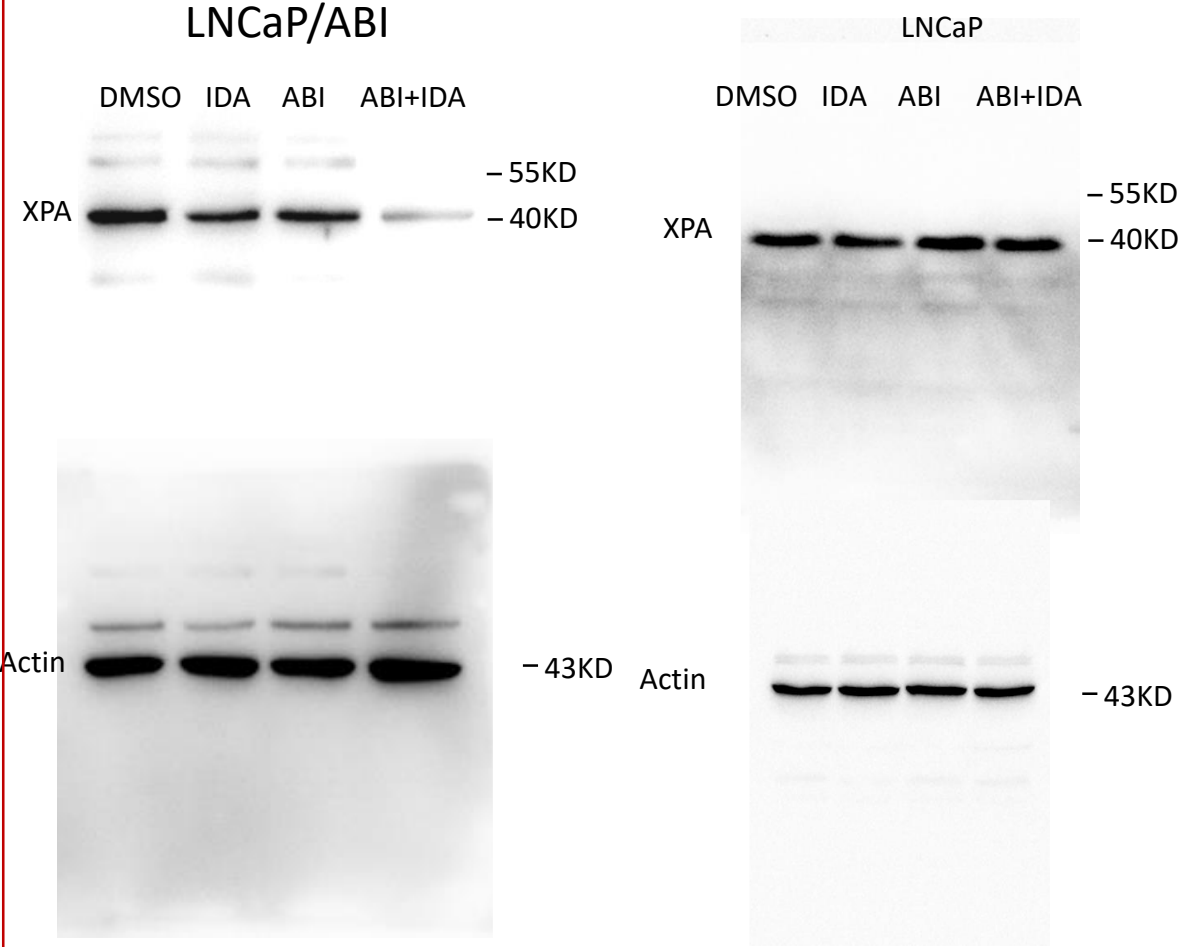

Fig. 3B

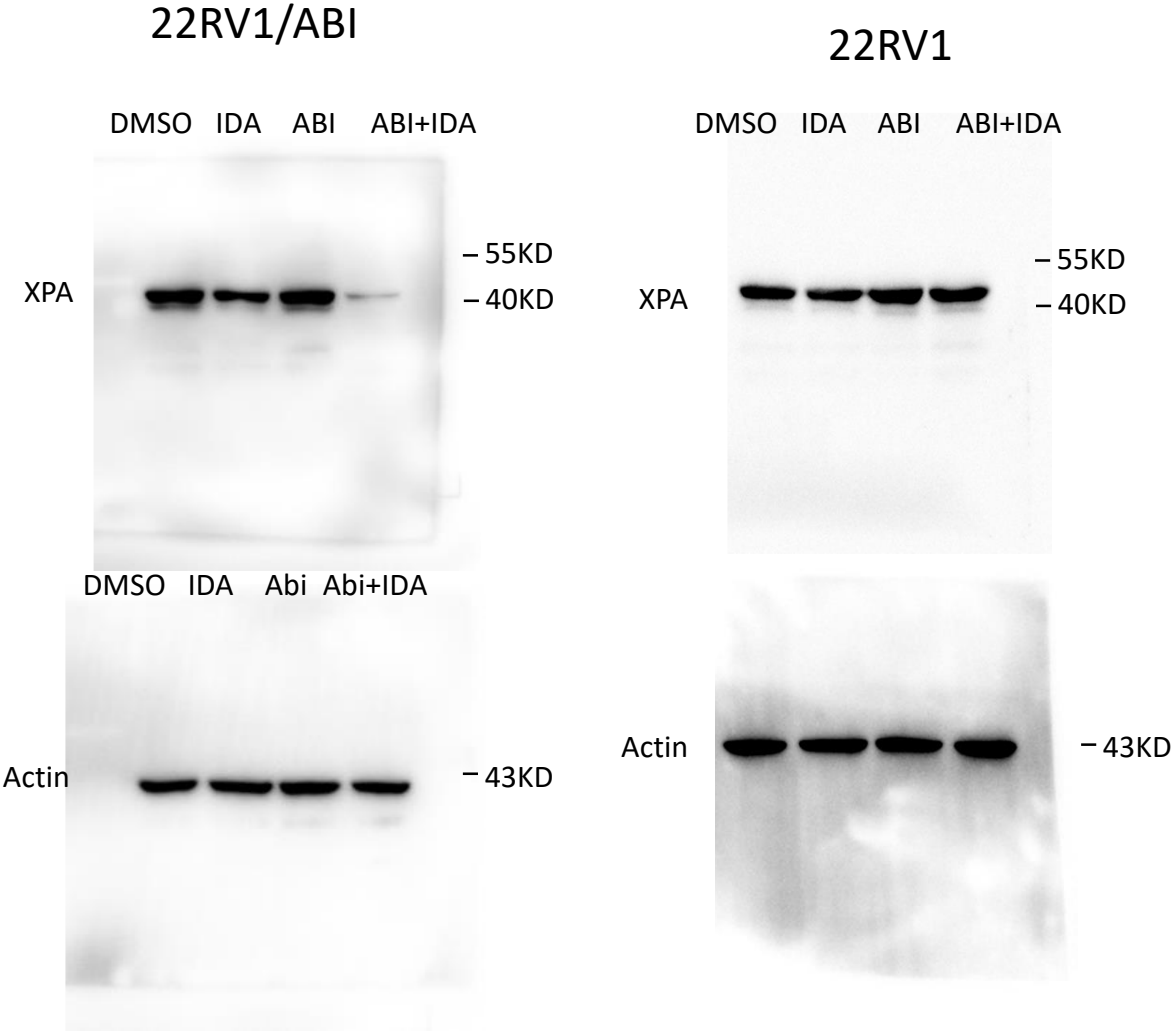

Fig. 3D

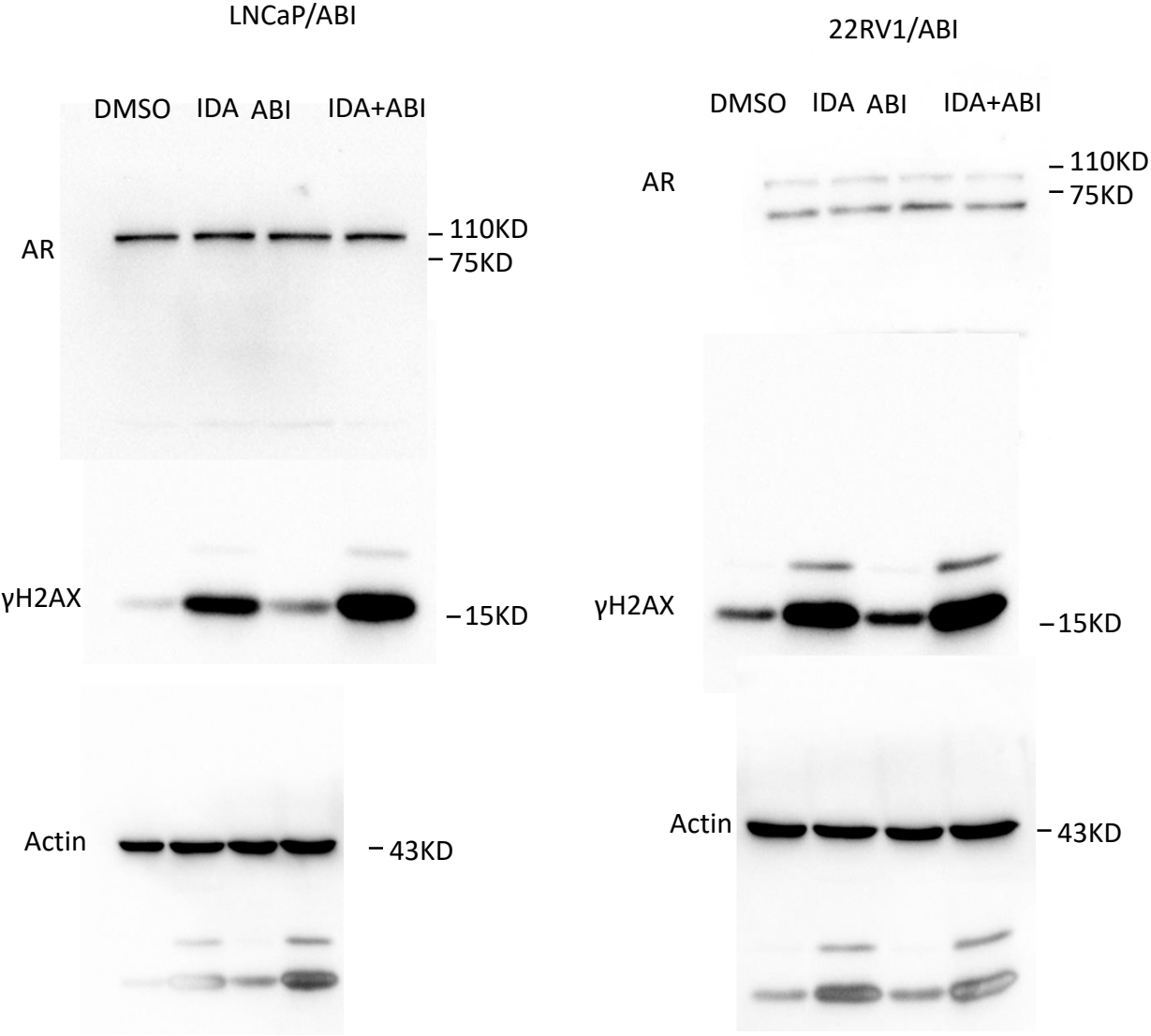

Fig. 4D

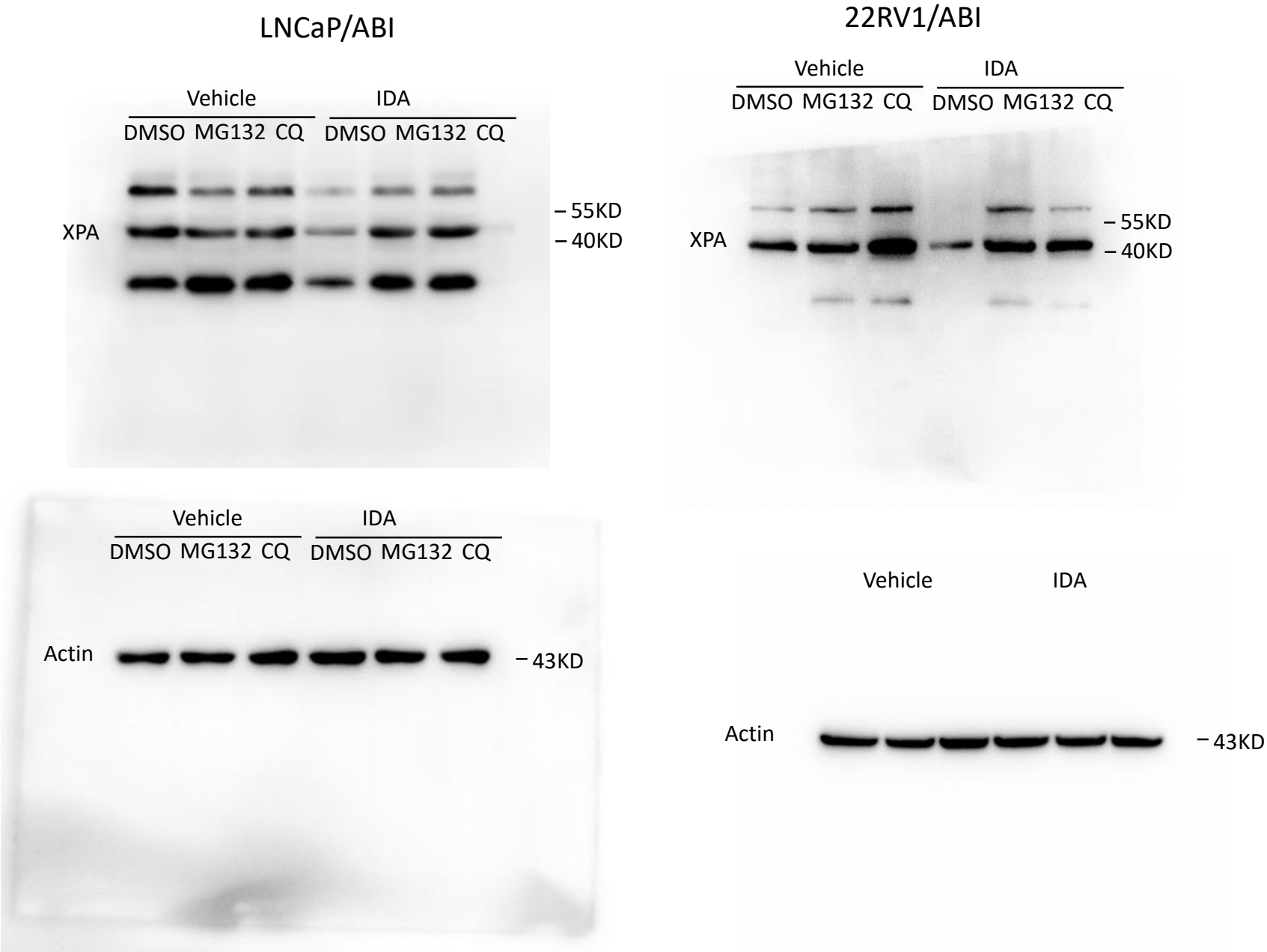

Fig. 4E

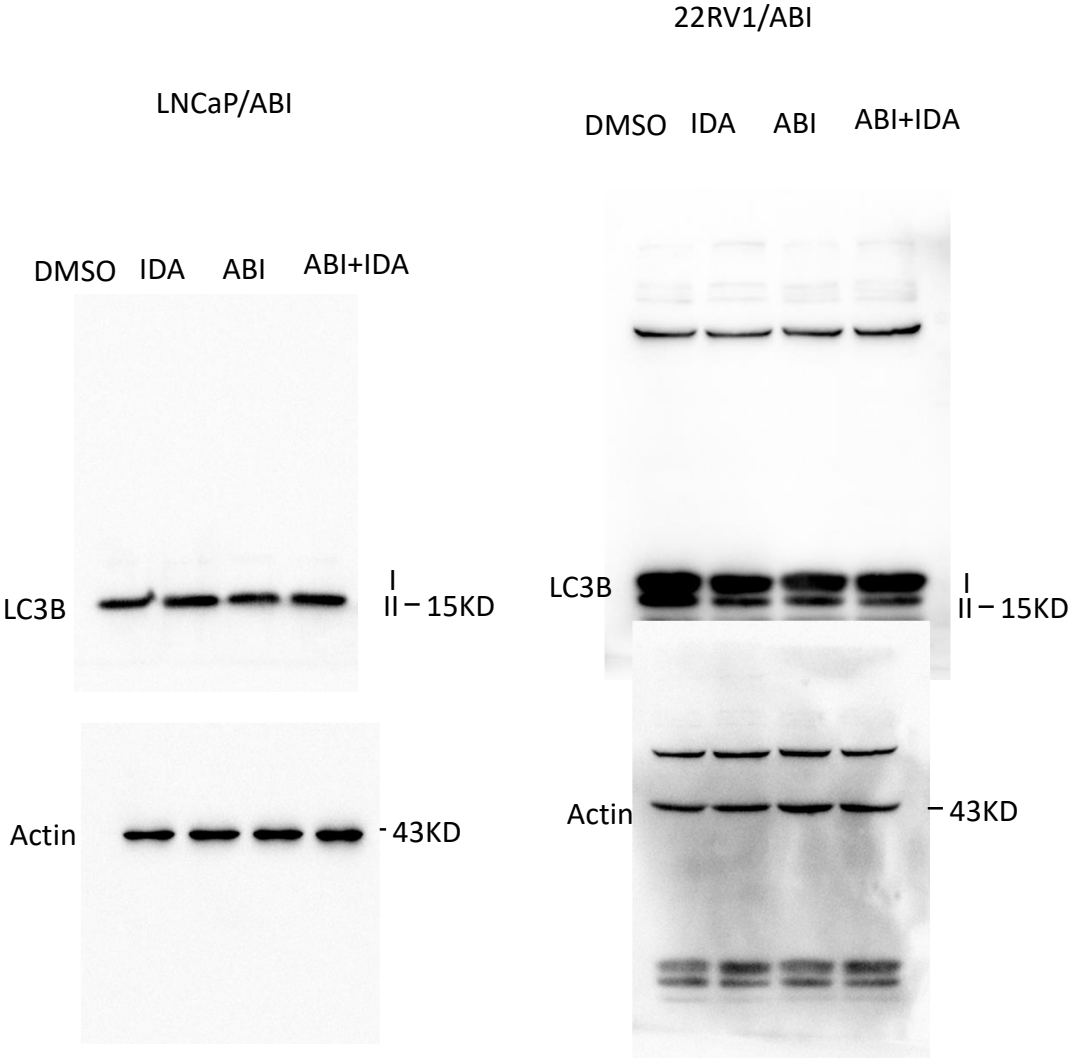

Fig4F

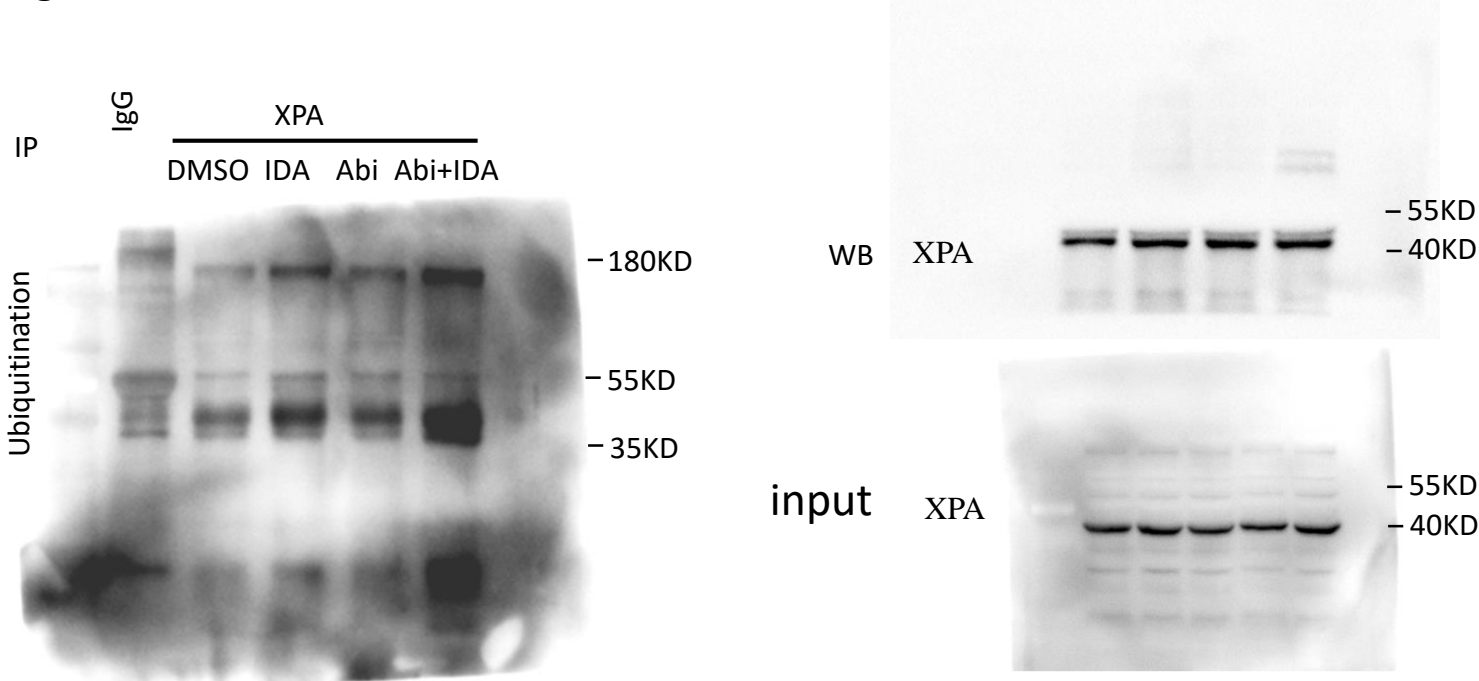

LNCaP/ENZ

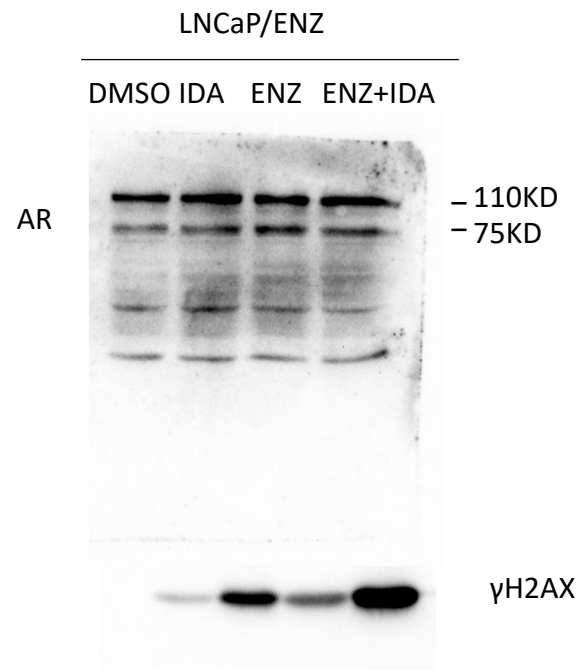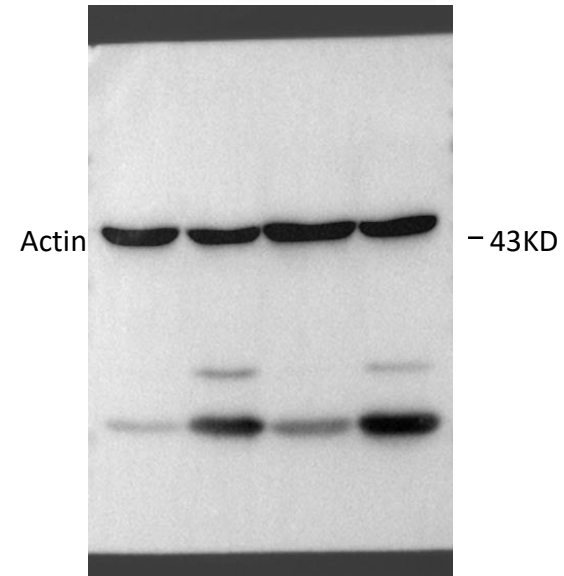

Fig. 5E

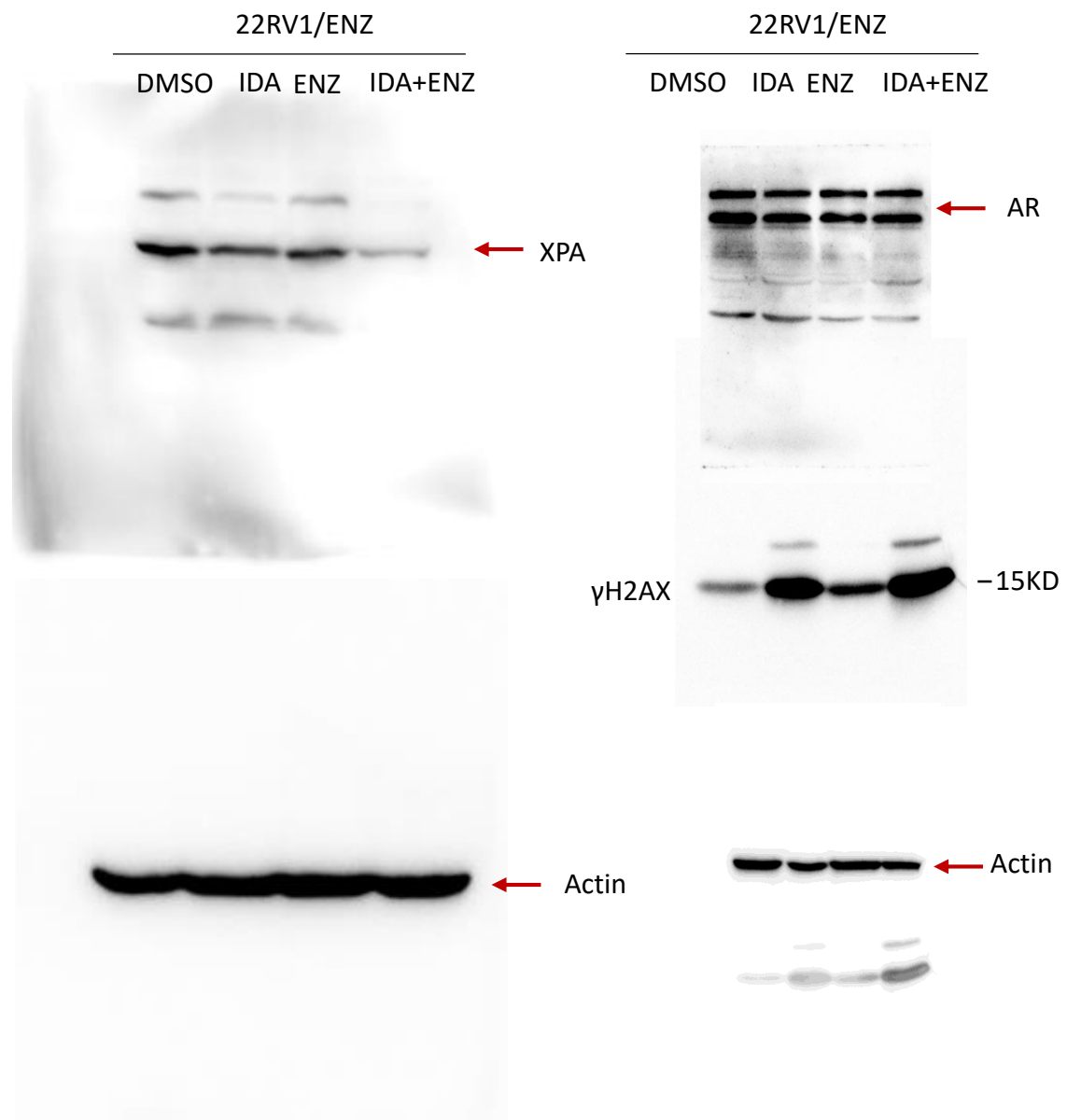

Fig. 5F

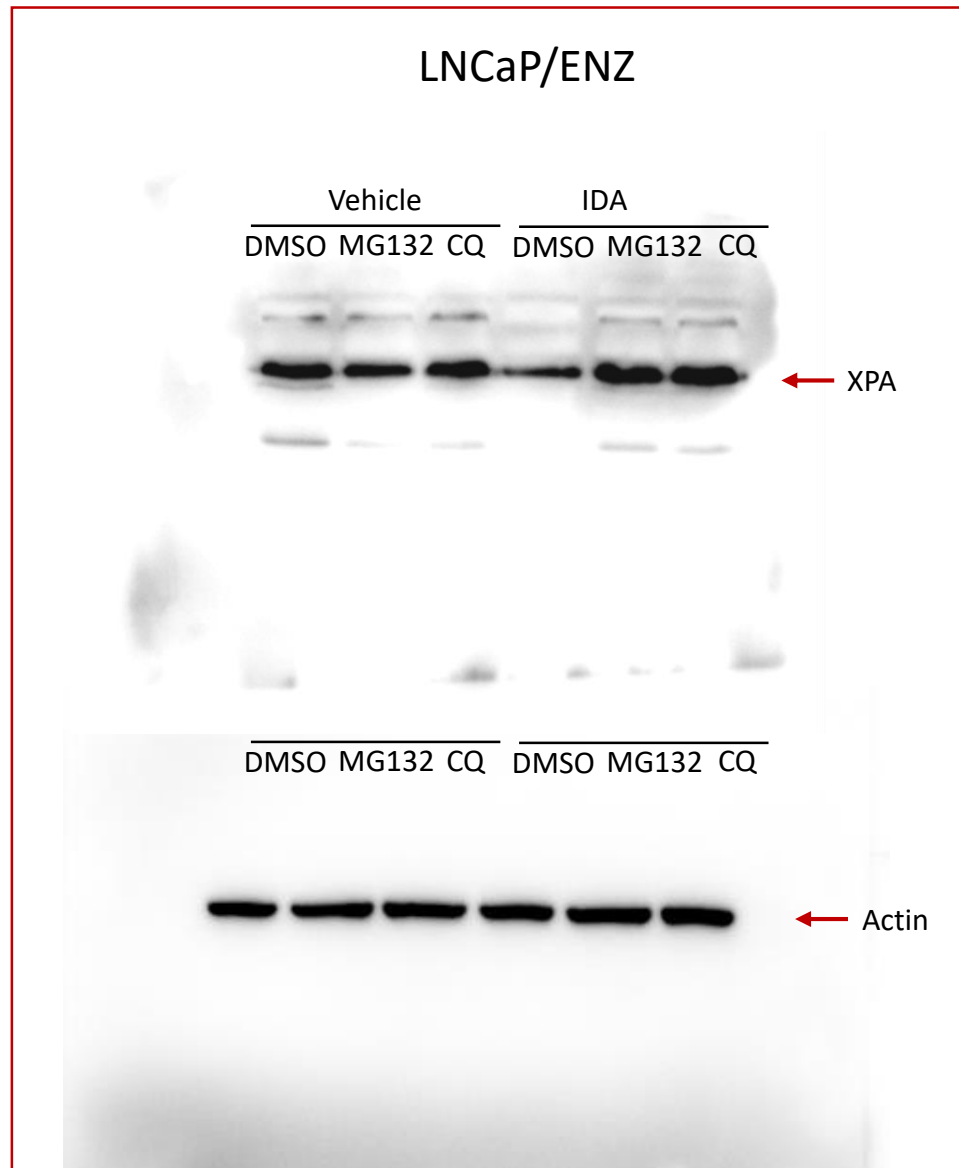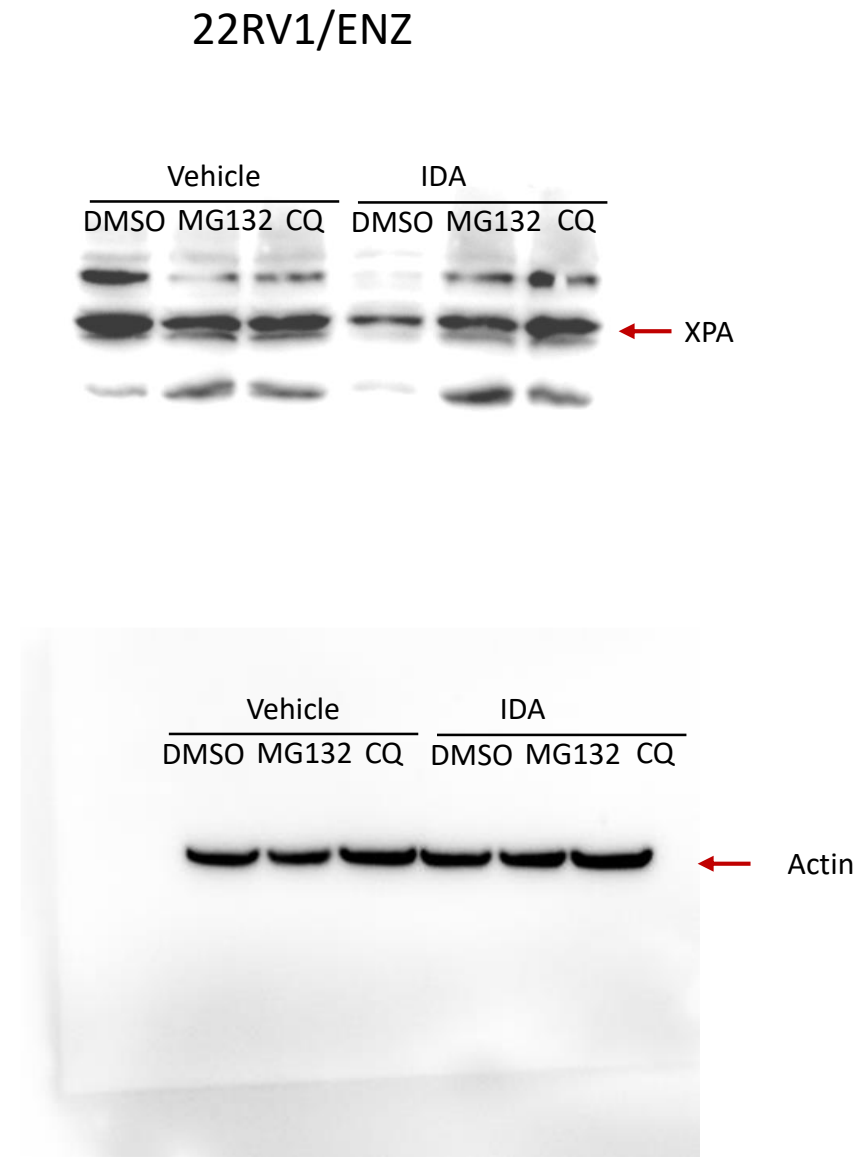

Fig. S5B

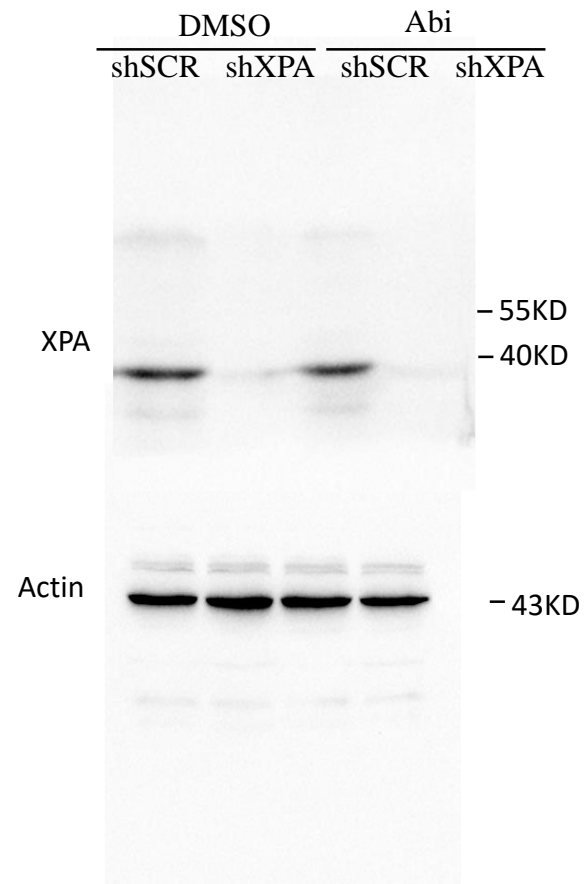

Supplement: Supplementary file 18 — Full uncut Western films [file 41419_2022_5490_MOESM18_ESM.pdf]
