# Supplementary material for: Idarubicin combats abiraterone and enzalutamide resistance in prostate cells via targeting XPA protein
Source: Cell Death Dis. 2022 Dec 12;13(12):1034. doi: 10.1038/s41419-022-05490-5 (PMC9744908; doi:10.1038/s41419-022-05490-5)
Supplement: Supplementary file 12 — Supplemental table legends [file 41419_2022_5490_MOESM12_ESM.docx]

Supplemental table legends

Table. S1 FDA-approved drug library list

Table. S2 Hits drugs in primary screen

Tab S3 Lost proteins

Tab S4 DAVID cluster

Tab. S5 Genes involving in the development of Abi resistance and decreased by IDA treatment
