# Supplementary material for: Idarubicin combats abiraterone and enzalutamide resistance in prostate cells via targeting XPA protein
Source: Cell Death Dis. 2022 Dec 12;13(12):1034. doi: 10.1038/s41419-022-05490-5 (PMC9744908; doi:10.1038/s41419-022-05490-5)
Supplement: Supplementary file 13 — Table s1 [file 41419_2022_5490_MOESM13_ESM.docx]

Table. S1 FDA-approved drug library list

| NO | Product Name | CAS No. |
| --- | --- | --- |
| 1 | Metergoline | 17692-51-2 |
| 2 | Cefmetazole (sodium) | 56796-39-5 |
| 3 | Nateglinide | 105816-04-4 |
| 4 | Indomethacin | 53-86-1 |
| 5 | Menaquinone-4 | 863-61-6 |
| 6 | Palonosetron (Hydrochloride) | 135729-62-3 |
| 7 | Ethamsylate | 2624-44-4 |
| 8 | Thio-TEPA | 52-24-4 |
| 9 | Naphazoline (hydrochloride) | 550-99-2 |
| 10 | Eplerenone | 107724-20-9 |
| 11 | Cabozantinib | 849217-68-1 |
| 12 | Sunitinib | 557795-19-4 |
| 13 | Chenodeoxycholic Acid | 474-25-9 |
| 14 | Ceritinib | 1032900-25-6 |
| 15 | Cefdinir | 91832-40-5 |
| 16 | Pemetrexed (disodium) | 150399-23-8 |
| 17 | Adiphenine (hydrochloride) | 50-42-0 |
| 18 | Niacin | 59-67-6 |
| 19 | Benzydamine (hydrochloride) | 132-69-4 |
| 20 | Cetilistat | 282526-98-1 |
| 21 | Diphenylpyraline (hydrochloride) | 132-18-3 |
| 22 | Stibogluconate (sodium) | 16037-91-5 |
| 23 | Cromolyn (sodium) | 15826-37-6 |
| 24 | Levodropropizine | 99291-25-5 |
| 25 | Fertirelin | 38234-21-8 |
| 26 | Cilnidipine | 132203-70-4 |
| 27 | Verapamil (hydrochloride) | 152-11-4 |
| 28 | Trazodone (hydrochloride) | 25332-39-2 |
| 29 | Solifenacin | 242478-37-1 |
| 30 | Diphenhydramine (hydrochloride) | 147-24-0 |
| 31 | Ornidazole | 16773-42-5 |
| 32 | Iohexol | 66108-95-0 |
| 33 | Perphenazine | 58-39-9 |
| 34 | Betahistine (dihydrochloride) | 5579-84-0 |
| 35 | Decitabine | 2353-33-5 |
| 36 | Reserpine | 50-55-5 |
| 37 | Amoxapine | 14028-44-5 |
| 38 | Dolasetron | 115956-12-2 |
| 39 | Fenspiride (Hydrochloride) | 5053-08-7 |
| 40 | Clofazimine | 2030-63-9 |
| 41 | Apronal | 528-92-7 |
| 42 | Idelalisib | 870281-82-6 |
| 43 | Anagrelide (hydrochloride) | 58579-51-4 |
| 44 | Cefprozil (monohydrate) | 121123-17-9 |
| 45 | Flecainide (acetate) | 54143-56-5 |
| 46 | Vorapaxar | 618385-01-6 |
| 47 | Naftidrofuryl (oxalate) | 3200-06-4 |
| 48 | Ruxolitinib | 941678-49-5 |
| 49 | Avibactam (sodium) | 1192491-61-4 |
| 50 | Thonzonium (bromide) | 553-08-2 |
| 51 | Fexofenadine (hydrochloride) | 153439-40-8 |
| 52 | Abacavir (sulfate) | 188062-50-2 |
| 53 | Thiamine hydrochloride | 67-03-8 |
| 54 | Flavin Adenine Dinucleotide Disodium | 84366-81-4 |
| 55 | Sulindac | 38194-50-2 |
| 56 | Tiopronin | 1953-02-2 |
| 57 | Thiamine monochloride | 59-43-8 |
| 58 | Palbociclib (isethionate) | 827022-33-3 |
| 59 | Desoximetasone | 382-67-2 |
| 60 | Fadrozole | 102676-47-1 |
| 61 | Nintedanib | 656247-17-5 |
| 62 | Aripiprazole | 129722-12-9 |
| 63 | Azasetron (hydrochloride) | 123040-16-4 |
| 64 | Estramustine (phosphate sodium) | 52205-73-9 |
| 65 | Sulfogaiacol | 1321-14-8 |
| 66 | Retinol | 68-26-8 |
| 67 | Lornoxicam | 70374-39-9 |
| 68 | Carbamoylcholine (chloride) | 51-83-2 |
| 69 | Atropine (sulfate monohydrate) | 5908-99-6 |
| 70 | Latrepirdine (dihydrochloride) | 97657-92-6 |
| 71 | Tetramisole (hydrochloride) | 5086-74-8 |
| 72 | Prednisolone (disodium phosphate) | 125-02-0 |
| 73 | Efinaconazole | 164650-44-6 |
| 74 | Riboflavin Tetrabutyrate | 752-56-7 |
| 75 | Trelagliptin (succinate) | 1029877-94-8 |
| 76 | Ramatroban | 116649-85-5 |
| 77 | Theophylline | 58-55-9 |
| 78 | Bromfenac (sodium hydrate) | 120638-55-3 |
| 79 | Cyclobenzaprine (hydrochloride) | 6202-23-9 |
| 80 | Sapacitabine | 151823-14-2 |
| 81 | Sildenafil (citrate) | 171599-83-0 |
| 82 | Bufexamac | 2438-72-4 |
| 83 | Tulobuterol hydrochloride | 56776-01-3 |
| 84 | Mupirocin | 12650-69-0 |
| 85 | Minoxidil | 38304-91-5 |
| 86 | Retinoic acid | 302-79-4 |
| 87 | Disulfiram | 97-77-8 |
| 88 | Obeticholic acid | 459789-99-2 |
| 89 | Ribociclib | 1211441-98-3 |
| 90 | Estradiol (cypionate) | 313-06-4 |
| 91 | Parecoxib | 198470-84-7 |
| 92 | Atenolol | 29122-68-7 |
| 93 | Scopolamine (hydrobromide) | 114-49-8 |
| 94 | Carbazochrome (sodium sulfonate) | 51460-26-5 |
| 95 | Salsalate | 552-94-3 |
| 96 | Tegaserod (maleate) | 189188-57-6 |
| 97 | Orphenadrine (citrate) | 4682-36-4 |
| 98 | Homatropine (Bromide) | 51-56-9 |
| 99 | Valsartan | 137862-53-4 |
| 100 | Succinylsulfathiazole | 116-43-8 |
| 101 | Azaperone | 1649-18-9 |
| 102 | Benzamil hydrochloride | 161804-20-2 |
| 103 | Diethylstilbestrol | 56-53-1 |
| 104 | Dehydrocholic acid | 81-23-2 |
| 105 | Hydrocortisone | 50-23-7 |
| 106 | Carbinoxamine maleate salt | 3505-38-2 |
| 107 | Ursodiol | 128-13-2 |
| 108 | Udenafil | 268203-93-6 |
| 109 | Piperacillin (sodium) | 59703-84-3 |
| 110 | Praziquantel | 55268-74-1 |
| 111 | Bifonazole | 60628-96-8 |
| 112 | Rivastigmine | 123441-03-2 |
| 113 | Sulfamonomethoxine | 1220-83-3 |
| 114 | Lercanidipine (hydrochloride) | 132866-11-6 |
| 115 | Propantheline (bromide) | 50-34-0 |
| 116 | Methacycline (hydrochloride) | 3963-95-9 |
| 117 | Betaxolol | 63659-18-7 |
| 118 | Pentostatin | 53910-25-1 |
| 119 | Toremifene (Citrate) | 89778-27-8 |
| 120 | Etidronic acid | 2809-21-4 |
| 121 | Etravirine | 269055-15-4 |
| 122 | Lacidipine | 103890-78-4 |
| 123 | Entecavir (monohydrate) | 209216-23-9 |
| 124 | Xylometazoline (hydrochloride) | 1218-35-5 |
| 125 | Cefixime | 79350-37-1 |
| 126 | Prostaglandin E2 | 363-24-6 |
| 127 | Gluconate (sodium) | 527-07-1 |
| 128 | Pralatrexate | 146464-95-1 |
| 129 | Neostigmine (methyl sulfate) | 51-60-5 |
| 130 | Carmustine | 154-93-8 |
| 131 | Benzyl alcohol | 100-51-6 |
| 132 | Galanthamine | 357-70-0 |
| 133 | Liothyronine | 6893-02-3 |
| 134 | Abiraterone (acetate) | 154229-18-2 |
| 135 | Clofarabine | 123318-82-1 |
| 136 | Methylprednisolone | 83-43-2 |
| 137 | Retapamulin | 224452-66-8 |
| 138 | Vinorelbine (ditartrate) | 125317-39-7 |
| 139 | Ketorolac (tromethamine salt) | 74103-07-4 |
| 140 | Sulfacarbamide | 547-44-4 |
| 141 | Tazarotene | 118292-40-3 |
| 142 | Tiagabine (hydrochloride) | 145821-59-6 |
| 143 | Sulfathiazole (sodium) | 144-74-1 |
| 144 | Pyrantel (pamoate) | 22204-24-6 |
| 145 | Rotundine | 483-14-7 |
| 146 | Anisodamine | 55869-99-3 |
| 147 | Doxorubicin (hydrochloride) | 25316-40-9 |
| 148 | Cefozopran (hydrochloride) | 113981-44-5 |
| 149 | Brimonidine (tartrate) | 70359-46-5 |
| 150 | Mebendazole | 31431-39-7 |
| 151 | Desogestrel | 54024-22-5 |
| 152 | Detomidine (hydrochloride) | 90038-01-0 |
| 153 | Pramipexole (dihydrochloride) | 104632-25-9 |
| 154 | Cefazolin (sodium) | 27164-46-1 |
| 155 | Ibrutinib | 936563-96-1 |
| 156 | Taltirelin | 103300-74-9 |
| 157 | Hydrocortisone (acetate) | 50-03-3 |
| 158 | Busulfan | 55-98-1 |
| 159 | Rofecoxib | 162011-90-7 |
| 160 | Goserelin (acetate) | 145781-92-6 |
| 161 | Eslicarbazepine (acetate) | 236395-14-5 |
| 162 | Ribociclib succinate | 1374639-75-4 |
| 163 | Tofogliflozin (hydrate) | 1201913-82-7 |
| 164 | Clevidipine | 167221-71-8 |
| 165 | L-(-)-α-Methyldopa (hydrate) | 41372-08-1 |
| 166 | 6-Mercaptopurine hydrate | 6112-76-1 |
| 167 | Desipramine hydrochloride | 58-28-6 |
| 168 | Cloxacillin (sodium monohydrate) | 7081-44-9 |
| 169 | 6-Acetamidohexanoic acid | 57-08-9 |
| 170 | Pasiniazid | 2066-89-9 |
| 171 | Adenosine | 58-61-7 |
| 172 | Mozavaptan | 137975-06-5 |
| 173 | Ruxolitinib (phosphate) | 1092939-17-7 |
| 174 | Guaifenesin | 93-14-1 |
| 175 | Maprotiline (hydrochloride) | 10347-81-6 |
| 176 | Iloperidone | 133454-47-4 |
| 177 | Chlorpheniramine (maleate) | 113-92-8 |
| 178 | Ceftibuten (dihydrate) | 118081-34-8 |
| 179 | Trelagliptin | 865759-25-7 |
| 180 | Pazopanib | 444731-52-6 |
| 181 | Rebamipide | 90098-04-7 |
| 182 | Alogliptin (Benzoate) | 850649-62-6 |
| 183 | Nafamostat (mesylate) | 82956-11-4 |
| 184 | Dimenhydrinate | 523-87-5 |
| 185 | Vilazodone | 163521-12-8 |
| 186 | Methylprednisolone succinate | 2921-57-5 |
| 187 | Dinoprost (tromethamine salt) | 38562-01-5 |
| 188 | Vinblastine (sulfate) | 143-67-9 |
| 189 | Piracetam | 7491-74-9 |
| 190 | Amoxicillin (sodium) | 34642-77-8 |
| 191 | Pindolol | 13523-86-9 |
| 192 | Prednisolone | 50-24-8 |
| 193 | Nelarabine | 121032-29-9 |
| 194 | Acarbose | 56180-94-0 |
| 195 | Acetazolamide | 59-66-5 |
| 196 | Enzalutamide | 915087-33-1 |
| 197 | Pimecrolimus | 137071-32-0 |
| 198 | Meloxicam | 71125-38-7 |
| 199 | Pixantrone (dimaleate) | 144675-97-8 |
| 200 | Chlorprothixene | 113-59-7 |
| 201 | Atovaquone | 95233-18-4 |
| 202 | Rosiglitazone (maleate) | 155141-29-0 |
| 203 | Zileuton | 111406-87-2 |
| 204 | Delavirdine (mesylate) | 147221-93-0 |
| 205 | Ibutilide (fumarate) | 122647-32-9 |
| 206 | Prilocaine | 721-50-6 |
| 207 | Gabexate (mesylate) | 56974-61-9 |
| 208 | Sodium copper chlorophyllin A | 11006-34-1 |
| 209 | Rotigotine | 99755-59-6 |
| 210 | Ifenprodil (tartrate) | 23210-58-4 |
| 211 | Quinapril (hydrochloride) | 82586-55-8 |
| 212 | Ramelteon | 196597-26-9 |
| 213 | Lifitegrast | 1025967-78-5 |
| 214 | Baricitinib (phosphate) | 1187595-84-1 |
| 215 | Histamine (phosphate) | 51-74-1 |
| 216 | Danofloxacin (mesylate) | 119478-55-6 |
| 217 | Reserpine (hydrochloride) | 16994-56-2 |
| 218 | Articaine (hydrochloride) | 23964-57-0 |
| 219 | Rilpivirine | 500287-72-9 |
| 220 | Afatinib | 850140-72-6 |
| 221 | Pomalidomide | 19171-19-8 |
| 222 | Benidipine (hydrochloride) | 91599-74-5 |
| 223 | Cloperastine fendizoate | 85187-37-7 |
| 224 | Nonivamide | 2444-46-4 |
| 225 | Milnacipran ((1S-cis) hydrochloride) | 175131-60-9 |
| 226 | Bexarotene | 153559-49-0 |
| 227 | Canagliflozin (hemihydrate) | 928672-86-0 |
| 228 | Alpha-Estradiol | 57-91-0 |
| 229 | Rufinamide | 106308-44-5 |
| 230 | Moroxydine (hydrochloride) | 3160-91-6 |
| 231 | Abacavir | 136470-78-5 |
| 232 | Naftopidil | 57149-07-2 |
| 233 | Dihydroergotamine (mesylate) | 6190-39-2 |
| 234 | Terazosin (hydrochloride dihydrate) | 70024-40-7 |
| 235 | Phenelzine (sulfate) | 156-51-4 |
| 236 | Idebenone | 58186-27-9 |
| 237 | Carbetocin | 37025-55-1 |
| 238 | Ornidazole (Levo-) | 166734-83-4 |
| 239 | Clevudine | 163252-36-6 |
| 240 | Didanosine | 69655-05-6 |
| 241 | Tamoxifen (Citrate) | 54965-24-1 |
| 242 | Allantoin | 97-59-6 |
| 243 | Edaravone | 89-25-8 |
| 244 | Altretamine | 645-05-6 |
| 245 | Epinastine | 80012-43-7 |
| 246 | Norethindrone acetate | 51-98-9 |
| 247 | Tetracaine | 94-24-6 |
| 248 | Febuxostat | 144060-53-7 |
| 249 | Ivacaftor | 873054-44-5 |
| 250 | Lenalidomide | 191732-72-6 |
| 251 | Clozapine | 5786-21-0 |
| 252 | Valbenazine | 1025504-45-3 |
| 253 | Daclatasvir (dihydrochloride) | 1009119-65-6 |
| 254 | Methoxsalen | 298-81-7 |
| 255 | Oxantel (pamoate) | 68813-55-8 |
| 256 | Malotilate | 59937-28-9 |
| 257 | Menadione bisulfite (sodium) | 130-37-0 |
| 258 | Tofacitinib (citrate) | 540737-29-9 |
| 259 | Bosentan (hydrate) | 157212-55-0 |
| 260 | Felbamate | 25451-15-4 |
| 261 | Felypressin | 56-59-7 |
| 262 | Bethanechol (chloride) | 590-63-6 |
| 263 | Chlorocresol | 59-50-7 |
| 264 | Ethynodiol (diacetate) | 297-76-7 |
| 265 | Orotic acid | 65-86-1 |
| 266 | Dapoxetine (hydrochloride) | 129938-20-1 |
| 267 | Milnacipran (hydrochloride) | 101152-94-7 |
| 268 | Ezetimibe | 163222-33-1 |
| 269 | Orlistat | 96829-58-2 |
| 270 | Diosmin | 520-27-4 |
| 271 | Propoxycaine (hydrochloride) | 550-83-4 |
| 272 | Trimethadione | 127-48-0 |
| 273 | Donepezil | 120014-06-4 |
| 274 | Ceftazidime | 72558-82-8 |
| 275 | Meticrane | 1084-65-7 |
| 276 | Azelnidipine | 123524-52-7 |
| 277 | Telotristat etiprate | 1137608-69-5 |
| 278 | Edrophonium (chloride) | 116-38-1 |
| 279 | Atorvastatin (hemicalcium salt) | 134523-03-8 |
| 280 | Gemfibrozil | 25812-30-0 |
| 281 | Iguratimod | 123663-49-0 |
| 282 | Tiratricol | 51-24-1 |
| 283 | Vismodegib | 879085-55-9 |
| 284 | Brompheniramine (maleate) | 980-71-2 |
| 285 | Ronidazole | 7681-76-7 |
| 286 | Luliconazole | 187164-19-8 |
| 287 | Vadadustat | 1000025-07-9 |
| 288 | Trifluridine | 70-00-8 |
| 289 | Isosorbide | 652-67-5 |
| 290 | Dantrolene (sodium hemiheptahydrate) | 24868-20-0 |
| 291 | Glimepiride | 93479-97-1 |
| 292 | Telotristat ethyl | 1033805-22-9 |
| 293 | Risedronate (sodium) | 115436-72-1 |
| 294 | Pamidronic acid | 40391-99-9 |
| 295 | Dequalinium (Chloride) | 522-51-0 |
| 296 | Ofloxacin | 82419-36-1 |
| 297 | Lumefantrine | 82186-77-4 |
| 298 | Alectinib | 1256580-46-7 |
| 299 | (R)-Lansoprazole | 138530-94-6 |
| 300 | Acamprosate (calcium) | 77337-73-6 |
| 301 | Furagin | 1672-88-4 |
| 302 | Methylthiouracil | 56-04-2 |
| 303 | Teneligliptin (hydrobromide) | 906093-29-6 |
| 304 | Pemirolast (potassium) | 100299-08-9 |
| 305 | Sitafloxacin (hydrate) | 163253-35-8 |
| 306 | Docetaxel | 114977-28-5 |
| 307 | Sucralfate | 54182-58-0 |
| 308 | Acrivastine | 87848-99-5 |
| 309 | Etofenamate | 30544-47-9 |
| 310 | Paliperidone | 144598-75-4 |
| 311 | Sapropterin (dihydrochloride) | 69056-38-8 |
| 312 | D-Sorbitol | 50-70-4 |
| 313 | Gefarnate | 51-77-4 |
| 314 | Emtricitabine | 143491-57-0 |
| 315 | Amantadine (hydrochloride) | 665-66-7 |
| 316 | Liothyronine (sodium) | 55-06-1 |
| 317 | Benfotiamine | 22457-89-2 |
| 318 | Trametinib | 871700-17-3 |
| 319 | Gatifloxacin | 112811-59-3 |
| 320 | Gefitinib (hydrochloride) | 184475-55-6 |
| 321 | Telmisartan | 144701-48-4 |
| 322 | Cevimeline (hydrochloride) | 107220-28-0 |
| 323 | Cobimetinib (hemifumarate) | 1369665-02-0 |
| 324 | Josamycin | 16846-24-5 |
| 325 | (+)-Ketoconazole | 142128-59-4 |
| 326 | Alectinib (Hydrochloride) | 1256589-74-8 |
| 327 | Vigabatrin (Hydrochloride) | 1391054-02-6 |
| 328 | Nimodipine | 66085-59-4 |
| 329 | Itraconazole | 84625-61-6 |
| 330 | Niraparib | 1038915-60-4 |
| 331 | Mebhydrolin (napadisylate) | 6153-33-9 |
| 332 | Balofloxacin | 127294-70-6 |
| 333 | Nimorazole | 6506-37-2 |
| 334 | Terbutaline (sulfate) | 23031-32-5 |
| 335 | Cetylpyridinium (chloride monohydrate) | 6004-24-6 |
| 336 | Nintedanib esylate | 656247-18-6 |
| 337 | Oxacillin (sodium monohydrate) | 7240-38-2 |
| 338 | Osimertinib mesylate | 1421373-66-1 |
| 339 | Ponatinib | 943319-70-8 |
| 340 | Irinotecan (hydrochloride trihydrate) | 136572-09-3 |
| 341 | Melphalan | 148-82-3 |
| 342 | Ropinirole (hydrochloride) | 91374-20-8 |
| 343 | Zoledronic acid (monohydrate) | 165800-06-6 |
| 344 | Toceranib | 356068-94-5 |
| 345 | Tedizolid | 856866-72-3 |
| 346 | Etodolac | 41340-25-4 |
| 347 | Pyrantel (tartrate) | 33401-94-4 |
| 348 | Leflunomide | 75706-12-6 |
| 349 | Fosphenytoin (disodium) | 92134-98-0 |
| 350 | Caspofungin (Acetate) | 179463-17-3 |
| 351 | Cefadroxil | 50370-12-2 |
| 352 | Pimozide | 2062-78-4 |
| 353 | Ziprasidone (hydrochloride monohydrate) | 138982-67-9 |
| 354 | Vorinostat | 149647-78-9 |
| 355 | Tenofovir alafenamide hemifumarate | 1392275-56-7 |
| 356 | Pipemidic acid | 51940-44-4 |
| 357 | Desvenlafaxine (succinate hydrate) | 386750-22-7 |
| 358 | Gadodiamide | 131410-48-5 |
| 359 | Oxethazaine | 126-27-2 |
| 360 | Ropivacaine hydrochloride | 98717-15-8 |
| 361 | Oseltamivir (acid) | 187227-45-8 |
| 362 | Irinotecan (hydrochloride) | 100286-90-6 |
| 363 | Miriplatin | 141977-79-9 |
| 364 | Dolutegravir (sodium) | 1051375-19-9 |
| 365 | Bisoctrizole | 103597-45-1 |
| 366 | Trilostane | 13647-35-3 |
| 367 | L-Thyroxine | 51-48-9 |
| 368 | Sugammadex (sodium) | 343306-79-6 |
| 369 | Metoprolol (Succinate) | 98418-47-4 |
| 370 | Dienogest | 65928-58-7 |
| 371 | Betaine (hydrochloride) | 590-46-5 |
| 372 | Cephalexin | 15686-71-2 |
| 373 | Trametinib (DMSO solvate) | 1187431-43-1 |
| 374 | Ozenoxacin | 245765-41-7 |
| 375 | Tetrabenazine | 58-46-8 |
| 376 | Vitamin B12 | 68-19-9 |
| 377 | Dolutegravir | 1051375-16-6 |
| 378 | Ginsenoside Rg3 | 14197-60-5 |
| 379 | Imiquimod | 99011-02-6 |
| 380 | Natamycin | 7681-93-8 |
| 381 | Silodosin | 160970-54-7 |
| 382 | Tropisetron (Hydrochloride) | 105826-92-4 |
| 383 | Sorafenib | 284461-73-0 |
| 384 | Acebutolol (hydrochloride) | 34381-68-5 |
| 385 | Clofibric acid | 882-09-7 |
| 386 | Isoconazole (nitrate) | 24168-96-5 |
| 387 | Valacyclovir (hydrochloride) | 124832-27-5 |
| 388 | Ethacridine (lactate monohydrate) | 6402-23-9 |
| 389 | Torsemide | 56211-40-6 |
| 390 | Lomustine | 13010-47-4 |
| 391 | Diltiazem (hydrochloride) | 33286-22-5 |
| 392 | Mirabegron | 223673-61-8 |
| 393 | Fimasartan | 247257-48-3 |
| 394 | Aztreonam | 78110-38-0 |
| 395 | Fluocinolone (Acetonide) | 67-73-2 |
| 396 | Naratriptan (hydrochloride) | 143388-64-1 |
| 397 | Mexiletine (hydrochloride) | 5370-01-4 |
| 398 | Isosorbide mononitrate | 16051-77-7 |
| 399 | Fingolimod (hydrochloride) | 162359-56-0 |
| 400 | Fluphenazine (dihydrochloride) | 146-56-5 |
| 401 | Eprosartan (mesylate) | 144143-96-4 |
| 402 | Hexachlorophene | 70-30-4 |
| 403 | Sulfamethoxazole | 723-46-6 |
| 404 | Decloxizine (dihydrochloride) | 13073-96-6 |
| 405 | Itopride (hydrochloride) | 122892-31-3 |
| 406 | Chlormadinone (acetate) | 302-22-7 |
| 407 | Ambrisentan | 177036-94-1 |
| 408 | Salicylic acid | 69-72-7 |
| 409 | Daclatasvir | 1009119-64-5 |
| 410 | Vilazodone (Hydrochloride) | 163521-08-2 |
| 411 | Melatonin | 73-31-4 |
| 412 | Loxapine | 1977-10-2 |
| 413 | Drofenine (hydrochloride) | 548-66-3 |
| 414 | Tripelennamine (hydrochloride) | 154-69-8 |
| 415 | Hydroxyprogesterone caproate | 630-56-8 |
| 416 | Atropine | 51-55-8 |
| 417 | Terbinafine | 91161-71-6 |
| 418 | Tinidazole | 19387-91-8 |
| 419 | Alibendol | 26750-81-2 |
| 420 | Protoporphyrin IX | 553-12-8 |
| 421 | Lidocaine (hydrochloride) | 73-78-9 |
| 422 | Metyrapone | 54-36-4 |
| 423 | Mitiglinide (Calcium) | 145525-41-3 |
| 424 | Vancomycin (hydrochloride) | 1404-93-9 |
| 425 | Benserazide hydrochloride (Synonyms: Serazide; Ro 4-4602) | 14919-77-8 |
| 426 | Safinamide | 133865-89-1 |
| 427 | Clorprenaline hydrochloride | 6933-90-0 |
| 428 | Sulfasalazine | 599-79-1 |
| 429 | Embelin | 550-24-3 |
| 430 | Bacampicillin (hydrochloride) | 37661-08-8 |
| 431 | Spironolactone | 52-01-7 |
| 432 | Tranilast | 53902-12-8 |
| 433 | Fidaxomicin | 873857-62-6 |
| 434 | Artemether | 71963-77-4 |
| 435 | Broxyquinoline | 521-74-4 |
| 436 | Amoxicillin | 26787-78-0 |
| 437 | Procarbazine (Hydrochloride) | 366-70-1 |
| 438 | Ipratropium (bromide) | 22254-24-6 |
| 439 | Alverine (citrate) | 5560-59-8 |
| 440 | Tavaborole | 174671-46-6 |
| 441 | Gestodene | 60282-87-3 |
| 442 | Etretinate | 54350-48-0 |
| 443 | Pitolisant (hydrochloride) | 903576-44-3 |
| 444 | Oxeladin (citrate) | 52432-72-1 |
| 445 | Homoharringtonine | 26833-87-4 |
| 446 | Oxaliplatin | 61825-94-3 |
| 447 | Amiloride hydrochloride dihydrate | 17440-83-4 |
| 448 | Clebopride (malate) | 57645-91-7 |
| 449 | Diclofenac | 15307-86-5 |
| 450 | Alprenolol (hydrochloride) | 13707-88-5 |
| 451 | Gefitinib | 184475-35-2 |
| 452 | Sertindole | 106516-24-9 |
| 453 | Erlotinib | 183321-74-6 |
| 454 | Linagliptin | 668270-12-0 |
| 455 | Ciprofibrate | 52214-84-3 |
| 456 | Benzbromarone | 3562-84-3 |
| 457 | Dienestrol | 84-17-3 |
| 458 | Cefsulodin (sodium) | 52152-93-9 |
| 459 | Cisapride | 81098-60-4 |
| 460 | Felbinac | 5728-52-9 |
| 461 | Benzyl benzoate | 120-51-4 |
| 462 | Chlorcyclizine (hydrochloride) | 14362-31-3 |
| 463 | Sodium tauroglycocholate | 41945-48-6 |
| 464 | Miglustat (hydrochloride) | 210110-90-0 |
| 465 | Eltrombopag (Olamine) | 496775-62-3 |
| 466 | Halcinonide | 3093-35-4 |
| 467 | Penfluridol | 26864-56-2 |
| 468 | Tolazamide | 1156-19-0 |
| 469 | Saquinavir | 127779-20-8 |
| 470 | Proglumide | 6620-60-6 |
| 471 | Azelastine (hydrochloride) | 79307-93-0 |
| 472 | Finasteride | 98319-26-7 |
| 473 | Warfarin | 81-81-2 |
| 474 | Fingolimod | 162359-55-9 |
| 475 | Lomerizine dihydrochloride | 101477-54-7 |
| 476 | Selumetinib | 606143-52-6 |
| 477 | Cephalothin (sodium) | 58-71-9 |
| 478 | Verteporfin | 129497-78-5 |
| 479 | Guanidine (hydrochloride) | 50-01-1 |
| 480 | Posaconazole | 171228-49-2 |
| 481 | Antipyrine | 60-80-0 |
| 482 | Triamcinolone | 124-94-7 |
| 483 | Betamipron | 3440-28-6 |
| 484 | Telaprevir | 402957-28-2 |
| 485 | Boceprevir | 394730-60-0 |
| 486 | Setiptiline (maleate) | 85650-57-3 |
| 487 | Benactyzine hydrochloride | 57-37-4 |
| 488 | Nabumetone | 42924-53-8 |
| 489 | Ciclopirox | 29342-05-0 |
| 490 | Miglitol | 72432-03-2 |
| 491 | Lafutidine | 118288-08-7 |
| 492 | Halobetasol (propionate) | 66852-54-8 |
| 493 | Sorafenib (Tosylate) | 475207-59-1 |
| 494 | Temsirolimus | 162635-04-3 |
| 495 | Proxyphylline | 603-00-9 |
| 496 | Flurbiprofen | 5104-49-4 |
| 497 | Dabrafenib | 1195765-45-7 |
| 498 | Etamivan | 304-84-7 |
| 499 | Simeprevir | 923604-59-5 |
| 500 | Mezlocillin (sodium) | 42057-22-7 |
| 501 | Alvimopan (monohydrate) | 1383577-62-5 |
| 502 | Temozolomide | 85622-93-1 |
| 503 | Dabigatran etexilate (mesylate) | 872728-81-9 |
| 504 | Oxybutynin (chloride) | 1508-65-2 |
| 505 | Rifabutin | 72559-06-9 |
| 506 | Prazosin (hydrochloride) | 19237-84-4 |
| 507 | Spectinomycin (dihydrochloride pentahydrate) | 22189-32-8 |
| 508 | Fludarabine (phosphate) | 75607-67-9 |
| 509 | Cefoselis (sulfate) | 122841-12-7 |
| 510 | Efonidipine (hydrochloride monoethanolate) | 111011-76-8 |
| 511 | Atracurium (besylate) | 64228-81-5 |
| 512 | Sulpiride | 15676-16-1 |
| 513 | Chlorquinaldol | 72-80-0 |
| 514 | Imatinib (Mesylate) | 220127-57-1 |
| 515 | Diloxanide furoate | 3736-81-0 |
| 516 | Tolvaptan | 150683-30-0 |
| 517 | Sulfamethizole | 144-82-1 |
| 518 | Silibinin | 22888-70-6 |
| 519 | Etofylline | 519-37-9 |
| 520 | Clomiphene (citrate) | 50-41-9 |
| 521 | Moxonidine | 75438-57-2 |
| 522 | Butamben | 94-25-7 |
| 523 | Cevimeline (hydrochloride hemihydrate) | 153504-70-2 |
| 524 | Clarithromycin | 81103-11-9 |
| 525 | Azilsartan | 147403-03-0 |
| 526 | Baricitinib | 1187594-09-7 |
| 527 | Fudosteine | 13189-98-5 |
| 528 | Rifaximin | 80621-81-4 |
| 529 | Trihexyphenidyl (hydrochloride) | 52-49-3 |
| 530 | Aliskiren (hemifumarate) | 173334-58-2 |
| 531 | Phenazopyridine (hydrochloride) | 136-40-3 |
| 532 | Dapagliflozin ((2S)-1,2-propanediol, hydrate) | 960404-48-2 |
| 533 | Vemurafenib | 918504-65-1 |
| 534 | Primidone | 125-33-7 |
| 535 | Betamethasone | 378-44-9 |
| 536 | Tenofovir (Disoproxil Fumarate) | 202138-50-9 |
| 537 | Pioglitazone (hydrochloride) | 112529-15-4 |
| 538 | Mosapride (citrate) | 112885-42-4 |
| 539 | Dimesna | 16208-51-8 |
| 540 | Lomitapide | 182431-12-5 |
| 541 | Ibuprofen | 15687-27-1 |
| 542 | Darifenacin (hydrobromide) | 133099-07-7 |
| 543 | Ketotifen (fumarate) | 34580-14-8 |
| 544 | Anethole (trithione) | 532-11-6 |
| 545 | Camostat (mesylate) | 59721-29-8 |
| 546 | Diphenmanil (methylsulfate) | 62-97-5 |
| 547 | Minaprine (dihydrochloride) | 25953-17-7 |
| 548 | Rupatadine (Fumarate) | 182349-12-8 |
| 549 | Aclidinium (Bromide) | 320345-99-1 |
| 550 | Octinoxate | 5466-77-3 |
| 551 | Emeramide | 351994-94-0 |
| 552 | Acetylcholine (chloride) | 60-31-1 |
| 553 | Afloqualone | 56287-74-2 |
| 554 | Roxithromycin | 80214-83-1 |
| 555 | Milrinone | 78415-72-2 |
| 556 | Chromocarb | 4940-39-0 |
| 557 | Taurochenodeoxycholic acid | 516-35-8 |
| 558 | Deferiprone | 30652-11-0 |
| 559 | Berberine (chloride hydrate) | 68030-18-2 |
| 560 | Salmeterol (xinafoate) | 94749-08-3 |
| 561 | Setiptiline | 57262-94-9 |
| 562 | Lenalidomide (hemihydrate) | 847871-99-2 |
| 563 | Tandospirone | 87760-53-0 |
| 564 | Phenytoin | 57-41-0 |
| 565 | Sulfaguanidine | 57-67-0 |
| 566 | Trifluoperazine (dihydrochloride) | 440-17-5 |
| 567 | Nitrofurantoin | 67-20-9 |
| 568 | Cladribine | 4291-63-8 |
| 569 | Tocofersolan | 9002-96-4 |
| 570 | Tenofovir alafenamide | 379270-37-8 |
| 571 | Erdosteine | 84611-23-4 |
| 572 | Oxybenzone | 131-57-7 |
| 573 | Vitamin K1 | 84-80-0 |
| 574 | Mequitazine | 29216-28-2 |
| 575 | Rocuronium (Bromide) | 119302-91-9 |
| 576 | Cefepime (Dihydrochloride Monohydrate) | 123171-59-5 |
| 577 | Acetylspiramycin | 24916-51-6 |
| 578 | Chlorhexidine | 55-56-1 |
| 579 | Proguanil | 500-92-5 |
| 580 | Gemcitabine (Hydrochloride) | 122111-03-9 |
| 581 | Tenoxicam | 59804-37-4 |
| 582 | Dihydroergotoxine (mesylate) | 8067-24-1 |
| 583 | Fenticonazole (Nitrate) | 73151-29-8 |
| 584 | Mycophenolic acid | 24280-93-1 |
| 585 | Mometasone furoate | 83919-23-7 |
| 586 | Tegafur | 17902-23-7 |
| 587 | Sulfamethazine | 57-68-1 |
| 588 | Cysteamine hydrochloride | 156-57-0 |
| 589 | Sodium Salicylate | 54-21-7 |
| 590 | Midecamycin | 35457-80-8 |
| 591 | Imipramine (hydrochloride) | 113-52-0 |
| 592 | Labetalol (hydrochloride) | 32780-64-6 |
| 593 | Apixaban | 503612-47-3 |
| 594 | Sildenafil | 139755-83-2 |
| 595 | Butylphthalide | 6066-49-5 |
| 596 | Pergolide (mesylate) | 66104-23-2 |
| 597 | Levosimendan | 141505-33-1 |
| 598 | Valrubicin | 56124-62-0 |
| 599 | Oxaprozin | 21256-18-8 |
| 600 | Olanzapine | 132539-06-1 |
| 601 | Topotecan (Hydrochloride) | 119413-54-6 |
| 602 | Cytidine | 65-46-3 |
| 603 | Methocarbamol | 532-03-6 |
| 604 | Ethionamide | 536-33-4 |
| 605 | Eptifibatide | 188627-80-7 |
| 606 | Ethacridine (lactate) | 1837-57-6 |
| 607 | Isoniazid | 54-85-3 |
| 608 | Epalrestat | 82159-09-9 |
| 609 | Lapatinib (ditosylate) | 388082-77-7 |
| 610 | Methazolamide | 554-57-4 |
| 611 | Mafenide (Acetate) | 13009-99-9 |
| 612 | Nifedipine | 21829-25-4 |
| 613 | Pioglitazone | 111025-46-8 |
| 614 | Levofloxacin | 100986-85-4 |
| 615 | Acetylcysteine | 616-91-1 |
| 616 | Vitamin D2 | 50-14-6 |
| 617 | Mitoxantrone (dihydrochloride) | 70476-82-3 |
| 618 | Oxiracetam | 62613-82-5 |
| 619 | Salmeterol | 89365-50-4 |
| 620 | Irinotecan | 97682-44-5 |
| 621 | Balsalazide | 80573-04-2 |
| 622 | Lesinurad | 878672-00-5 |
| 623 | Raltegravir | 518048-05-0 |
| 624 | Danazol | 17230-88-5 |
| 625 | Neostigmine (Bromide) | 114-80-7 |
| 626 | Docusate (Sodium) | 577-11-7 |
| 627 | Saxagliptin | 361442-04-8 |
| 628 | Tilorone (dihydrochloride) | 27591-69-1 |
| 629 | Levobunolol (hydrochloride) | 27912-14-7 |
| 630 | Vidarabine | 5536-17-4 |
| 631 | Amiodarone (hydrochloride) | 19774-82-4 |
| 632 | Palbociclib (hydrochloride) | 827022-32-2 |
| 633 | Sodium gualenate | 6223-35-4 |
| 634 | Nikethamide | 59-26-7 |
| 635 | Nizatidine | 76963-41-2 |
| 636 | Doripenem (monohydrate) | 364622-82-2 |
| 637 | Etomidate | 33125-97-2 |
| 638 | Propranolol (hydrochloride) | 318-98-9 |
| 639 | Simvastatin | 79902-63-9 |
| 640 | Oxcarbazepine | 28721-07-5 |
| 641 | Chlorphenoxamine | 77-38-3 |
| 642 | Pentoxifylline | 6493-05-6 |
| 643 | Repaglinide | 135062-02-1 |
| 644 | Chlormezanone | 80-77-3 |
| 645 | Carbidopa | 28860-95-9 |
| 646 | Acetohexamide | 968-81-0 |
| 647 | Rosuvastatin (Calcium) | 147098-20-2 |
| 648 | Betamethasone dipropionate | 5593-20-4 |
| 649 | Methimazole | 60-56-0 |
| 650 | Rosiglitazone | 122320-73-4 |
| 651 | Montelukast (sodium) | 151767-02-1 |
| 652 | Avanafil | 330784-47-9 |
| 653 | Amsacrine | 51264-14-3 |
| 654 | Iopanoic acid | 96-83-3 |
| 655 | Kasugamycin (hydrochloride hydrate) | 200132-83-8 |
| 656 | Granisetron (Hydrochloride) | 107007-99-8 |
| 657 | Ramipril | 87333-19-5 |
| 658 | Azlocillin (sodium salt) | 37091-65-9 |
| 659 | Benztropine (mesylate) | 132-17-2 |
| 660 | Ketoconazole | 65277-42-1 |
| 661 | Cobicistat | 1004316-88-4 |
| 662 | Terconazole | 67915-31-5 |
| 663 | Amitriptyline (hydrochloride) | 549-18-8 |
| 664 | Efavirenz | 154598-52-4 |
| 665 | Azathioprine | 446-86-6 |
| 666 | Prasugrel | 150322-43-3 |
| 667 | Glycopyrrolate | 596-51-0 |
| 668 | Oxytetracycline | 79-57-2 |
| 669 | Tadalafil | 171596-29-5 |
| 670 | Dexmedetomidine (hydrochloride) | 145108-58-3 |
| 671 | Clemizole (hydrochloride) | 1163-36-6 |
| 672 | Cyproheptadine (hydrochloride sesquihydrate) | 41354-29-4 |
| 673 | Tauroursodeoxycholate (Sodium) | 35807-85-3 |
| 674 | Alfuzosin | 81403-80-7 |
| 675 | Clemastine (fumarate) | 14976-57-9 |
| 676 | Cyclandelate | 456-59-7 |
| 677 | Nitrendipine | 39562-70-4 |
| 678 | Ticarcillin (disodium) | 4697-14-7 |
| 679 | Cilostazol | 73963-72-1 |
| 680 | Carbimazole | 22232-54-8 |
| 681 | Sulfaphenazole | 526-08-9 |
| 682 | Olsalazine (Disodium) | 6054-98-4 |
| 683 | Alosetron (Hydrochloride) | 122852-69-1 |
| 684 | Nebivolol (hydrochloride) | 152520-56-4 |
| 685 | Zidovudine | 30516-87-1 |
| 686 | Metoclopramide (hydrochloride hydrate) | 54143-57-6 |
| 687 | Phenytoin (sodium) | 630-93-3 |
| 688 | Dofetilide | 115256-11-6 |
| 689 | Fenofibric acid | 42017-89-0 |
| 690 | Imatinib | 152459-95-5 |
| 691 | (±)-Bisoprolol (hemifumarate) | 104344-23-2 |
| 692 | Ospemifene | 128607-22-7 |
| 693 | Clinofibrate | 30299-08-2 |
| 694 | Bucladesine (sodium salt) | 16980-89-5 |
| 695 | Pyrithioxin (dihydrochloride) | 10049-83-9 |
| 696 | Niclosamide | 50-65-7 |
| 697 | Phthalylsulfacetamide | 131-69-1 |
| 698 | Cefmenoxime (hydrochloride) | 75738-58-8 |
| 699 | Captopril | 62571-86-2 |
| 700 | Bendazol | 621-72-7 |
| 701 | Amezinium (methylsulfate) | 30578-37-1 |
| 702 | Cinnarizine | 298-57-7 |
| 703 | Dasatinib | 302962-49-8 |
| 704 | Sertaconazole (nitrate) | 99592-39-9 |
| 705 | Nicorandil | 65141-46-0 |
| 706 | Dabrafenib (Mesylate) | 1195768-06-9 |
| 707 | Flumethasone | 2135-17-3 |
| 708 | L-Ascorbic acid sodium salt | 134-03-2 |
| 709 | Nevirapine | 129618-40-2 |
| 710 | Oxolamine (citrate) | 1949-20-8 |
| 711 | Spiramycin | 8025-81-8 |
| 712 | Ambroxol | 18683-91-5 |
| 713 | Olmesartan (medoxomil) | 144689-63-4 |
| 714 | Canagliflozin | 842133-18-0 |
| 715 | Ledipasvir | 1256388-51-8 |
| 716 | 4-Aminopyridine | 504-24-5 |
| 717 | Prothionamide | 14222-60-7 |
| 718 | Vincamine | 1617-90-9 |
| 719 | Hydroxyfasudil (hydrochloride) | 155558-32-0 |
| 720 | Bambuterol hydrochloride | 81732-46-9 |
| 721 | Ibuprofen piconol | 64622-45-3 |
| 722 | Histamine | 51-45-6 |
| 723 | Faropenem daloxate | 141702-36-5 |
| 724 | Lapatinib | 231277-92-2 |
| 725 | Ibudilast | 50847-11-5 |
| 726 | Quinestrol | 152-43-2 |
| 727 | Triflusal | 322-79-2 |
| 728 | 10-Undecenoic acid | 112-38-9 |
| 729 | D-Mannitol | 69-65-8 |
| 730 | Perindopril (erbumine) | 107133-36-8 |
| 731 | Sitagliptin (phosphate monohydrate) | 654671-77-9 |
| 732 | Regadenoson | 313348-27-5 |
| 733 | Rabeprazole (sodium) | 117976-90-6 |
| 734 | Sulbactam | 68373-14-8 |
| 735 | Icotinib | 610798-31-7 |
| 736 | Dolasetron (Mesylate hydrate) | 878143-33-0 |
| 737 | Sulfisoxazole | 127-69-5 |
| 738 | Butenafine (Hydrochloride) | 101827-46-7 |
| 739 | Darunavir | 206361-99-1 |
| 740 | Benazepril (hydrochloride) | 86541-74-4 |
| 741 | Pizotifen | 15574-96-6 |
| 742 | Cefotaxime (sodium salt) | 64485-93-4 |
| 743 | Roxatidine (Acetate Hydrochloride) | 93793-83-0 |
| 744 | Abarelix | 183552-38-7 |
| 745 | Aceclofenac | 89796-99-6 |
| 746 | Duloxetine (hydrochloride) | 136434-34-9 |
| 747 | Pirarubicin (Hydrochloride) | 95343-20-7 |
| 748 | Raloxifene (hydrochloride) | 82640-04-8 |
| 749 | Glafenine (hydrochloride) | 65513-72-6 |
| 750 | Nelfinavir (Mesylate) | 159989-65-8 |
| 751 | Doxazosin (mesylate) | 77883-43-3 |
| 752 | Haloperidol | 52-86-8 |
| 753 | Ampiroxicam | 99464-64-9 |
| 754 | Terlipressin | 14636-12-5 |
| 755 | Roflumilast | 162401-32-3 |
| 756 | Carbamazepine | 298-46-4 |
| 757 | Pravastatin (sodium) | 81131-70-6 |
| 758 | Floxuridine | 50-91-9 |
| 759 | Temocapril (hydrochloride) | 110221-44-8 |
| 760 | Fluticasone (propionate) | 80474-14-2 |
| 761 | Piperidolate | 82-98-4 |
| 762 | Vildagliptin | 274901-16-5 |
| 763 | Atomoxetine (hydrochloride) | 82248-59-7 |
| 764 | Zofenopril (calcium) | 81938-43-4 |
| 765 | Esomeprazole magnesium | 161973-10-0 |
| 766 | Ciclopirox (olamine) | 41621-49-2 |
| 767 | D-Cycloserine | 68-41-7 |
| 768 | Bucladesine (calcium salt) | 938448-87-4 |
| 769 | Bepridil hydrochloride | 68099-86-5 |
| 770 | Tazobactam | 89786-04-9 |
| 771 | Naproxen (sodium) | 26159-34-2 |
| 772 | Tiotropium (Bromide) | 136310-93-5 |
| 773 | Misoprostol | 59122-46-2 |
| 774 | Dasatinib (hydrochloride) | 854001-07-3 |
| 775 | Rasagiline (mesylate) | 161735-79-1 |
| 776 | Ribociclib hydrochloride | 1211443-80-9 |
| 777 | Trandolapril | 87679-37-6 |
| 778 | Imidafenacin | 170105-16-5 |
| 779 | Pranlukast | 103177-37-3 |
| 780 | Topiroxostat | 577778-58-6 |
| 781 | Azatadine (dimaleate) | 3978-86-7 |
| 782 | Acetylleucine | 99-15-0 |
| 783 | Bisacodyl | 603-50-9 |
| 784 | Artesunate | 88495-63-0 |
| 785 | Voriconazole | 137234-62-9 |
| 786 | Niflumic acid | 4394-00-7 |
| 787 | Gimeracil | 103766-25-2 |
| 788 | Dutasteride | 164656-23-9 |
| 789 | Penciclovir | 39809-25-1 |
| 790 | Clobetasol propionate | 25122-46-7 |
| 791 | Mebhydrolin | 524-81-2 |
| 792 | Afatinib (dimaleate) | 850140-73-7 |
| 793 | Bilastine | 202189-78-4 |
| 794 | Sulfameter | 651-06-9 |
| 795 | Garenoxacin (Mesylate hydrate) | 223652-90-2 |
| 796 | Ribavirin | 36791-04-5 |
| 797 | Metoclopramide | 364-62-5 |
| 798 | Carprofen | 53716-49-7 |
| 799 | Pranoprofen | 52549-17-4 |
| 800 | Chlorthalidone | 77-36-1 |
| 801 | Lincomycin (hydrochloride hydrate) | 7179-49-9 |
| 802 | Levosulpiride | 23672-07-3 |
| 803 | Manidipine (dihydrochloride) | 89226-75-5 |
| 804 | Tetrahydrobiopterin | 17528-72-2 |
| 805 | Capecitabine | 154361-50-9 |
| 806 | Olprinone (Hydrochloride) | 119615-63-3 |
| 807 | Diflunisal | 22494-42-4 |
| 808 | Levobetaxolol (hydrochloride) | 116209-55-3 |
| 809 | Erythromycin | 114-07-8 |
| 810 | Budesonide | 51333-22-3 |
| 811 | Stiripentol | 49763-96-4 |
| 812 | Fenofibrate | 49562-28-9 |
| 813 | Netupitant | 290297-26-6 |
| 814 | Octenidine (dihydrochloride) | 70775-75-6 |
| 815 | Amoxicillin (trihydrate) | 61336-70-7 |
| 816 | Asenapine (hydrochloride) | 1412458-61-7 |
| 817 | Agomelatine (hydrochloride) | 1176316-99-6 |
| 818 | Folic acid | 59-30-3 |
| 819 | Novobiocin (Sodium) | 1476-53-5 |
| 820 | Lactulose | 4618-18-2 |
| 821 | Estrone | 53-16-7 |
| 822 | D-Pantothenic acid (sodium) | 867-81-2 |
| 823 | Naproxen | 22204-53-1 |
| 824 | Chlorhexidine (digluconate) | 18472-51-0 |
| 825 | Levocarnitine propionate (hydrochloride) | 119793-66-7 |
| 826 | Dronedarone | 141626-36-0 |
| 827 | Galanthamine (hydrobromide) | 1953-04-4 |
| 828 | Rimantadine (hydrochloride) | 1501-84-4 |
| 829 | Sulbutiamine | 3286-46-2 |
| 830 | Deoxycholic acid | 83-44-3 |
| 831 | Tipiracil (hydrochloride) | 183204-72-0 |
| 832 | Dichlorphenamide | 120-97-8 |
| 833 | Lasofoxifene (Tartrate) | 190791-29-8 |
| 834 | Solifenacin (Succinate) | 242478-38-2 |
| 835 | Brimonidine | 59803-98-4 |
| 836 | Fluconazole | 86386-73-4 |
| 837 | Gadodiamide (hydrate) | 122795-43-1 |
| 838 | Diacerein | 13739-02-1 |
| 839 | Permethrin | 52645-53-1 |
| 840 | Valproic acid | 99-66-1 |
| 841 | Desvenlafaxine | 93413-62-8 |
| 842 | Tetracycline (hydrochloride) | 64-75-5 |
| 843 | Canrenone | 976-71-6 |
| 844 | Rolapitant | 552292-08-7 |
| 845 | Dapsone | 80-08-0 |
| 846 | Econazole (nitrate) | 24169-02-6 |
| 847 | Desloratadine | 100643-71-8 |
| 848 | Vortioxetine | 508233-74-7 |
| 849 | Moclobemide | 71320-77-9 |
| 850 | Indacaterol (maleate) | 753498-25-8 |
| 851 | Nifuratel | 4936-47-4 |
| 852 | Vonoprazan Fumarate | 1260141-27-2 |
| 853 | Belotecan (hydrochloride) | 213819-48-8 |
| 854 | Pramiracetam | 68497-62-1 |
| 855 | Nilvadipine | 75530-68-6 |
| 856 | Hydroxyurea | 127-07-1 |
| 857 | Pentamidine (isethionate) | 140-64-7 |
| 858 | Ouabain (Octahydrate) | 11018-89-6 |
| 859 | Velpatasvir | 1377049-84-7 |
| 860 | Flutamide | 13311-84-7 |
| 861 | Estropipate | 7280-37-7 |
| 862 | Lansoprazole | 103577-45-3 |
| 863 | Acetohydroxamic acid | 546-88-3 |
| 864 | Pemetrexed | 137281-23-3 |
| 865 | Colchicine | 64-86-8 |
| 866 | Racecadotril | 81110-73-8 |
| 867 | Methicillin (sodium salt) | 132-92-3 |
| 868 | Carteolol hydrochloride | 51781-21-6 |
| 869 | Spectinomycin (dihydrochloride) | 21736-83-4 |
| 870 | Lovastatin | 75330-75-5 |
| 871 | Probucol | 23288-49-5 |
| 872 | Carmofur | 61422-45-5 |
| 873 | Ombitasvir | 1258226-87-7 |
| 874 | Ranitidine (hydrochloride) | 66357-59-3 |
| 875 | 6-Thioguanine | 154-42-7 |
| 876 | Dabigatran etexilate | 211915-06-9 |
| 877 | Paclitaxel | 33069-62-4 |
| 878 | Vardenafil (hydrochloride) | 224785-91-5 |
| 879 | Clofibrate | 637-07-0 |
| 880 | Estradiol | 50-28-2 |
| 881 | Ceritinib dihydrochloride | 1380575-43-8 |
| 882 | Paroxetine (hydrochloride) | 78246-49-8 |
| 883 | Pitavastatin (Calcium) | 147526-32-7 |
| 884 | Naftifine (hydrochloride) | 65473-14-5 |
| 885 | sn-Glycero-3-phosphocholine | 28319-77-9 |
| 886 | Ixazomib | 1072833-77-2 |
| 887 | Triamterene | 396-01-0 |
| 888 | Triflupromazine (hydrochloride) | 1098-60-8 |
| 889 | Selamectin | 220119-17-5 |
| 890 | Dexchlorpheniramine (maleate) | 2438-32-6 |
| 891 | Prulifloxacin | 123447-62-1 |
| 892 | Pazopanib (Hydrochloride) | 635702-64-6 |
| 893 | Dyclonine (hydrochloride) | 536-43-6 |
| 894 | L-Ornithine | 70-26-8 |
| 895 | Phenindione | 83-12-5 |
| 896 | Elvitegravir | 697761-98-1 |
| 897 | Fusidic acid (sodium salt) | 751-94-0 |
| 898 | Deflazacort | 14484-47-0 |
| 899 | Candesartan (Cilexetil) | 145040-37-5 |
| 900 | Piroctone olamine | 68890-66-4 |
| 901 | Promazine (hydrochloride) | 53-60-1 |
| 902 | Piperidolate (hydrochloride) | 129-77-1 |
| 903 | Enasidenib (mesylate) | 1650550-25-6 |
| 904 | Fondaparinux (sodium) | 114870-03-0 |
| 905 | Framycetin | 119-04-0 |
| 906 | Meclizine (dihydrochloride) | 1104-22-9 |
| 907 | Pyrimethamine | 58-14-0 |
| 908 | Plerixafor | 110078-46-1 |
| 909 | Azithromycin | 83905-01-5 |
| 910 | Citicoline sodium salt | 33818-15-4 |
| 911 | Riluzole hydrochloride | 850608-87-6 |
| 912 | Medroxyprogesterone (acetate) | 71-58-9 |
| 913 | Choline Fenofibrate | 856676-23-8 |
| 914 | Pirfenidone | 53179-13-8 |
| 915 | Citric acid (trilithium salt tetrahydrate) | 6080-58-6 |
| 916 | Doxycycline (hyclate) | 24390-14-5 |
| 917 | Teriflunomide | 163451-81-8 |
| 918 | Edoxaban (tosylate monohydrate) | 1229194-11-9 |
| 919 | Cortisone (acetate) | 50-04-4 |
| 920 | Crizotinib | 877399-52-5 |
| 921 | Eltrombopag | 496775-61-2 |
| 922 | Cilazapril (monohydrate) | 92077-78-6 |
| 923 | Pamidronate (disodium pentahydrate) | 109552-15-0 |
| 924 | Cariprazine | 839712-12-8 |
| 925 | Nilotinib | 641571-10-0 |
| 926 | Cidofovir | 113852-37-2 |
| 927 | Lomefloxacin (hydrochloride) | 98079-52-8 |
| 928 | Fosinopril (sodium) | 88889-14-9 |
| 929 | Cisatracurium (besylate) | 96946-42-8 |
| 930 | Enasidenib | 1446502-11-9 |
| 931 | Moxalactam (sodium salt) | 64953-12-4 |
| 932 | Erlotinib (Hydrochloride) | 183319-69-9 |
| 933 | Cariprazine (hydrochloride) | 1083076-69-0 |
| 934 | Piromidic acid | 19562-30-2 |
| 935 | Besifloxacin (Hydrochloride) | 405165-61-9 |
| 936 | Butoconazole (nitrate) | 64872-77-1 |
| 937 | Carfilzomib | 868540-17-4 |
| 938 | Cetirizine (dihydrochloride) | 83881-52-1 |
| 939 | Famciclovir | 104227-87-4 |
| 940 | Apremilast | 608141-41-9 |
| 941 | Dichlorisone acetate | 79-61-8 |
| 942 | Tenofovir (hydrate) | 206184-49-8 |
| 943 | Enoxacin (hydrate) | 84294-96-2 |
| 944 | Dimemorfan (phosphate) | 36304-84-4 |
| 945 | Mifepristone | 84371-65-3 |
| 946 | Ibandronate (Sodium Monohydrate) | 138926-19-9 |
| 947 | Domperidone | 57808-66-9 |
| 948 | Tioconazole | 65899-73-2 |
| 949 | Gallic acid | 149-91-7 |
| 950 | Etoposide | 33419-42-0 |
| 951 | Betrixaban | 330942-05-7 |
| 952 | Omeprazole | 73590-58-6 |
| 953 | Ledipasvir (D-tartrate) | 1502654-87-6 |
| 954 | Cabergoline | 81409-90-7 |
| 955 | Oxyclozanide | 2277-92-1 |
| 956 | Oxytocin (acetate) | 6233-83-6 |
| 957 | Dicloxacillin (Sodium hydrate) | 13412-64-1 |
| 958 | Fulvestrant | 129453-61-8 |
| 959 | Cefonicid (sodium) | 61270-78-8 |
| 960 | Regorafenib (monohydrate) | 1019206-88-2 |
| 961 | Glucosamine (hydrochloride) | 66-84-2 |
| 962 | Chlorambucil | 305-03-3 |
| 963 | Cytarabine | 147-94-4 |
| 964 | Avobenzone | 70356-09-1 |
| 965 | Nafcillin (sodium monohydrate) | 7177-50-6 |
| 966 | Nitroprusside (disodium dihydrate) | 13755-38-9 |
| 967 | Ketanserin | 74050-98-9 |
| 968 | Clofoctol | 37693-01-9 |
| 969 | Quetiapine | 111974-69-7 |
| 970 | Elafibranor | 923978-27-2 |
| 971 | Aspirin | 50-78-2 |
| 972 | Sarpogrelate (hydrochloride) | 135159-51-2 |
| 973 | L-Ascorbic acid | 50-81-7 |
| 974 | Anidulafungin | 166663-25-8 |
| 975 | Quinidine hydrochloride monohydrate | 6151-40-2 |
| 976 | Pazufloxacin (mesylate) | 163680-77-1 |
| 977 | Bendamustine (hydrochloride) | 3543-75-7 |
| 978 | Primaquine (Diphosphate) | 63-45-6 |
| 979 | Buflomedil (hydrochloride) | 35543-24-9 |
| 980 | Domiphen (bromide) | 538-71-6 |
| 981 | Diflorasone | 2557-49-5 |
| 982 | Loxapine (succinate) | 27833-64-3 |
| 983 | Carbetapentane (citrate) | 23142-01-0 |
| 984 | Quinine (hydrochloride dihydrate) | 6119-47-7 |
| 985 | Phentolamine (mesylate) | 65-28-1 |
| 986 | Letrozole | 112809-51-5 |
| 987 | Aliskiren | 173334-57-1 |
| 988 | Cinoxacin | 28657-80-9 |
| 989 | Vortioxetine (hydrobromide) | 960203-27-4 |
| 990 | Rivaroxaban | 366789-02-8 |
| 991 | Sodium diatrizoate | 737-31-5 |
| 992 | Bestatin | 58970-76-6 |
| 993 | 5-Aminosalicylic Acid | 89-57-6 |
| 994 | Olmesartan | 144689-24-7 |
| 995 | Doxapram (hydrochloride hydrate) | 7081-53-0 |
| 996 | Miconazole (nitrate) | 22832-87-7 |
| 997 | α-Lipoic Acid | 1077-28-7 |
| 998 | Saquinavir (Mesylate) | 149845-06-7 |
| 999 | Thalidomide | 50-35-1 |
| 1000 | Droperidol | 548-73-2 |
| 1001 | Diiodohydroxyquinoline | 83-73-8 |
| 1002 | Gramicidin | 1405-97-6 |
| 1003 | Metipranolol hydrochloride | 36592-77-5 |
| 1004 | Delamanid | 681492-22-8 |
| 1005 | Cefditoren (Pivoxil) | 117467-28-4 |
| 1006 | Ropivacaine (hydrochloride monohydrate) | 132112-35-7 |
| 1007 | Amifampridine | 54-96-6 |
| 1008 | Tolazoline (hydrochloride) | 59-97-2 |
| 1009 | Cinacalcet (hydrochloride) | 364782-34-3 |
| 1010 | Tosufloxacin (tosylate hydrate) | 1400591-39-0 |
| 1011 | Cortisone | 53-06-5 |
| 1012 | Gamithromycin | 145435-72-9 |
| 1013 | Lesinurad (sodium) | 1151516-14-1 |
| 1014 | Gestrinone | 16320-04-0 |
| 1015 | Piroxicam | 36322-90-4 |
| 1016 | Cephradine | 38821-53-3 |
| 1017 | Chlorothiazide | 58-94-6 |
| 1018 | Pimavanserin | 706779-91-1 |
| 1019 | Irsogladine | 57381-26-7 |
| 1020 | Bemegride | 64-65-3 |
| 1021 | Megestrol (Acetate) | 595-33-5 |
| 1022 | Agomelatine | 138112-76-2 |
| 1023 | Brivudine | 69304-47-8 |
| 1024 | Teneligliptin | 760937-92-6 |
| 1025 | Sulfadiazine | 68-35-9 |
| 1026 | Amlodipine (besylate) | 111470-99-6 |
| 1027 | Doxylamine (succinate) | 562-10-7 |
| 1028 | Nefazodone (hydrochloride) | 82752-99-6 |
| 1029 | Allopurinol | 315-30-0 |
| 1030 | Octreotide (acetate) | 79517-01-4 |
| 1031 | Loteprednol Etabonate | 82034-46-6 |
| 1032 | Leuprolide Acetate | 74381-53-6 |
| 1033 | Pidotimod | 121808-62-6 |
| 1034 | Meclofenoxate (hydrochloride) | 3685-84-5 |
| 1035 | Oxaceprol | 33996-33-7 |
| 1036 | Olopatadine (hydrochloride) | 140462-76-6 |
| 1037 | Mepyramine maleate | 59-33-6 |
| 1038 | Zonisamide | 68291-97-4 |
| 1039 | Propafenone (hydrochloride) | 34183-22-7 |
| 1040 | Cabazitaxel | 183133-96-2 |
| 1041 | Glipizide | 29094-61-9 |
| 1042 | Cinobufotalin | 1108-68-5 |
| 1043 | 3-Methyl-7-chloro-1,2,4-benzothiadiazine 1,1-dioxide | 364-98-7 |
| 1044 | Candesartan | 139481-59-7 |
| 1045 | Pefloxacin (mesylate) | 70458-95-6 |
| 1046 | Phenoxybenzamine (hydrochloride) | 63-92-3 |
| 1047 | Lamotrigine | 84057-84-1 |
| 1048 | Amisulpride | 71675-85-9 |
| 1049 | Pyrazinamide | 98-96-4 |
| 1050 | Resorcinol | 108-46-3 |
| 1051 | Tedizolid (phosphate) | 856867-55-5 |
| 1052 | Ajmaline | 4360-12-7 |
| 1053 | Ketanserin (tartrate) | 83846-83-7 |
| 1054 | Dapagliflozin | 461432-26-8 |
| 1055 | Metronidazole | 443-48-1 |
| 1056 | Rutin | 153-18-4 |
| 1057 | Atazanavir (sulfate) | 229975-97-7 |
| 1058 | Guanfacine (hydrochloride) | 29110-48-3 |
| 1059 | Guacetisal | 55482-89-8 |
| 1060 | Methscopolamine (bromide) | 155-41-9 |
| 1061 | Chlorzoxazone | 95-25-0 |
| 1062 | Quetiapine (fumarate) | 111974-72-2 |
| 1063 | Cefamandole (nafate) | 42540-40-9 |
| 1064 | Rucaparib (phosphate) | 459868-92-9 |
| 1065 | Trapidil | 15421-84-8 |
| 1066 | Norepinephrine (bitartrate monohydrate) | 108341-18-0 |
| 1067 | Ivermectin | 70288-86-7 |
| 1068 | Levamisole (hydrochloride) | 16595-80-5 |
| 1069 | Sodium Picosulfate | 10040-45-6 |
| 1070 | Daunorubicin (Hydrochloride) | 23541-50-6 |
| 1071 | Dorzolamide (hydrochloride) | 130693-82-2 |
| 1072 | Podofilox | 518-28-5 |
| 1073 | Raltegravir (potassium salt) | 871038-72-1 |
| 1074 | Nimesulide | 51803-78-2 |
| 1075 | Pralidoxime (chloride) | 51-15-0 |
| 1076 | Tamibarotene | 94497-51-5 |
| 1077 | 6α-Methylprednisolone 21-hemisuccinate (sodium salt) | 2375-03-3 |
| 1078 | 1-Docosanol | 661-19-8 |
| 1079 | Cinacalcet | 226256-56-0 |
| 1080 | Lumacaftor | 936727-05-8 |
| 1081 | Eflornithine (hydrochloride, hydrate) | 96020-91-6 |
| 1082 | Ivabradine (hydrochloride) | 148849-67-6 |
| 1083 | Ciclesonide | 126544-47-6 |
| 1084 | Ganciclovir | 82410-32-0 |
| 1085 | Riluzole | 1744-22-5 |
| 1086 | Treosulfan | 299-75-2 |
| 1087 | Bedaquiline (fumarate) | 845533-86-0 |
| 1088 | Scopolamine butylbromide | 149-64-4 |
| 1089 | Tigecycline | 220620-09-7 |
| 1090 | Fenoprofen (Calcium hydrate) | 71720-56-4 |
| 1091 | Tirofiban (hydrochloride monohydrate) | 150915-40-5 |
| 1092 | Flavoxate (hydrochloride) | 3717-88-2 |
| 1093 | Hydroxyzine (dihydrochloride) | 2192-20-3 |
| 1094 | Ulipristal (acetate) | 126784-99-4 |
| 1095 | Cefoxitin (sodium) | 33564-30-6 |
| 1096 | Osalmid | 526-18-1 |
| 1097 | Nitroxoline | 4008-48-4 |
| 1098 | Belinostat | 866323-14-0 |
| 1099 | Dithranol | 1143-38-0 |
| 1100 | Irbesartan | 138402-11-6 |
| 1101 | Nedocromil | 69049-73-6 |
| 1102 | Niraparib tosylate | 1038915-73-9 |
| 1103 | Cinepazide (Maleate) | 26328-04-1 |
| 1104 | Molsidomine | 25717-80-0 |
| 1105 | Cabozantinib (S-malate) | 1140909-48-3 |
| 1106 | Azelaic acid | 123-99-9 |
| 1107 | Clopidogrel (hydrogen sulfate) | 120202-66-6 |
| 1108 | Octocrylene | 6197-30-4 |
| 1109 | Bedaquiline | 843663-66-1 |
| 1110 | Lidocaine | 137-58-6 |
| 1111 | Nortriptyline (hydrochloride) | 894-71-3 |
| 1112 | Tamoxifen | 10540-29-1 |
| 1113 | Dronedarone (Hydrochloride) | 141625-93-6 |
| 1114 | Nitisinone | 104206-65-7 |
| 1115 | Ataluren | 775304-57-9 |
| 1116 | (R)-(-)-Phenylephrine (hydrochloride) | 61-76-7 |
| 1117 | Piribedil | 3605-01-4 |
| 1118 | Hexylresorcinol | 136-77-6 |
| 1119 | Metolazone | 17560-51-9 |
| 1120 | Fluoxetine (hydrochloride) | 56296-78-7 |
| 1121 | Clindamycin (hydrochloride) | 21462-39-5 |
| 1122 | Chlormethine (hydrochloride) | 55-86-7 |
| 1123 | Nicardipine (Hydrochloride) | 54527-84-3 |
| 1124 | Succimer | 304-55-2 |
| 1125 | Trimethobenzamide hydrochloride | 554-92-7 |
| 1126 | Diatrizoic acid | 117-96-4 |
| 1127 | Deferasirox | 201530-41-8 |
| 1128 | Seratrodast | 112665-43-7 |
| 1129 | Crizotinib (hydrochloride) | 1415560-69-8 |
| 1130 | Secnidazole | 3366-95-8 |
| 1131 | Digitoxin | 71-63-6 |
| 1132 | Enalapril (maleate) | 76095-16-4 |
| 1133 | Pantoprazole (sodium) | 138786-67-1 |
| 1134 | Bromocriptine (mesylate) | 22260-51-1 |
| 1135 | Rauwolscine (hydrochloride) | 6211-32-1 |
| 1136 | Adapalene | 106685-40-9 |
| 1137 | Tizanidine (hydrochloride) | 64461-82-1 |
| 1138 | Riociguat | 625115-55-1 |
| 1139 | Flumazenil | 78755-81-4 |
| 1140 | Sodium phenylbutyrate | 1716-12-7 |
| 1141 | Tasimelteon | 609799-22-6 |
| 1142 | Nadifloxacin | 124858-35-1 |
| 1143 | Gliclazide | 21187-98-4 |
| 1144 | Propylthiouracil | 51-52-5 |
| 1145 | Pancuronium (dibromide) | 15500-66-0 |
| 1146 | Chlorpropamide | 94-20-2 |
| 1147 | Flibanserin | 167933-07-5 |
| 1148 | Probenecid | 57-66-9 |
| 1149 | Difluprednate | 23674-86-4 |
| 1150 | Cobimetinib | 934660-93-2 |
| 1151 | Dimethyl fumarate | 624-49-7 |
| 1152 | Eicosapentaenoic Acid | 10417-94-4 |
| 1153 | Alprenolol | 13655-52-2 |
| 1154 | Clonidine (hydrochloride) | 4205-91-8 |
| 1155 | Bromhexine (hydrochloride) | 611-75-6 |
| 1156 | Alcaftadine | 147084-10-4 |
| 1157 | Iproniazid (phosphate) | 305-33-9 |
| 1158 | Etoricoxib | 202409-33-4 |
| 1159 | Esmolol (hydrochloride) | 81161-17-3 |
| 1160 | Diphenidol (hydrochloride) | 3254-89-5 |
| 1161 | Ethosuximide | 77-67-8 |
| 1162 | Ritonavir | 155213-67-5 |
| 1163 | Mirtazapine | 85650-52-8 |
| 1164 | Famotidine | 76824-35-6 |
| 1165 | 9-Aminoacridine | 90-45-9 |
| 1166 | 5-Azacytidine | 320-67-2 |
| 1167 | Oxiconazole nitrate | 64211-46-7 |
| 1168 | Trimetazidine (dihydrochloride) | 13171-25-0 |
| 1169 | Nilotinib (monohydrochloride monohydrate) | 923288-90-8 |
| 1170 | Isotretinoin | 4759-48-2 |
| 1171 | Dasabuvir | 1132935-63-7 |
| 1172 | Mepivacaine (hydrochloride) | 1722-62-9 |
| 1173 | Perhexiline maleate | 6724-53-4 |
| 1174 | Tetrahydrozoline (hydrochloride) | 522-48-5 |
| 1175 | Nifuroxazide | 965-52-6 |
| 1176 | Doxofylline | 69975-86-6 |
| 1177 | Vinpocetine | 42971-09-5 |
| 1178 | Progesterone | 57-83-0 |
| 1179 | Uridin | 58-96-8 |
| 1180 | Mitoxantrone | 65271-80-9 |
| 1181 | Indapamide | 26807-65-8 |
| 1182 | Citric acid | 77-92-9 |
| 1183 | Anagliptin | 739366-20-2 |
| 1184 | Bicalutamide | 90357-06-5 |
| 1185 | Dexamethasone (acetate) | 1177-87-3 |
| 1186 | Empagliflozin | 864070-44-0 |
| 1187 | Conivaptan (hydrochloride) | 168626-94-6 |
| 1188 | Digoxin | 20830-75-5 |
| 1189 | Danthron | 117-10-2 |
| 1190 | Entrectinib | 1108743-60-7 |
| 1191 | Bronopol | 52-51-7 |
| 1192 | Deferoxamine (mesylate) | 138-14-7 |
| 1193 | Tafluprost | 209860-87-7 |
| 1194 | Tolfenamic Acid | 13710-19-5 |
| 1195 | LCZ696 | 936623-90-4 |
| 1196 | Exemestane | 107868-30-4 |
| 1197 | Methotrexate | 59-05-2 |
| 1198 | Ticlopidine (hydrochloride) | 53885-35-1 |
| 1199 | Asenapine (maleate) | 85650-56-2 |
| 1200 | Solifenacin (hydrochloride) | 180468-39-7 |
| 1201 | Ertapenem sodium | 153773-82-1 |
| 1202 | Macitentan | 441798-33-0 |
| 1203 | Pranlukast (hemihydrate) | 150821-03-7 |
| 1204 | 6-Aminocaproic acid | 60-32-2 |
| 1205 | Levobupivacaine (hydrochloride) | 27262-48-2 |
| 1206 | Brinzolamide | 138890-62-7 |
| 1207 | 5-Fluorouracil | 51-21-8 |
| 1208 | Dexamethasone | 50-02-2 |
| 1209 | Levofloxacin (hydrate) | 138199-71-0 |
| 1210 | Levetiracetam | 102767-28-2 |
| 1211 | Ampicillin (sodium) | 69-52-3 |
| 1212 | Heptaminol (hydrochloride) | 543-15-7 |
| 1213 | Epirubicin (hydrochloride) | 56390-09-1 |
| 1214 | Doxifluridine | 3094-09-5 |
| 1215 | Fomepizole | 7554-65-6 |
| 1216 | Istradefylline | 155270-99-8 |
| 1217 | Ramosetron (Hydrochloride) | 132907-72-3 |
| 1218 | Alvimopan (dihydrate) | 170098-38-1 |
| 1219 | Crotamiton | 483-63-6 |
| 1220 | Hexaminolevulinate (hydrochloride) | 140898-91-5 |
| 1221 | Gastrodin | 62499-27-8 |
| 1222 | Firocoxib | 189954-96-9 |
| 1223 | L-Epinephrine (Bitartrate) | 51-42-3 |
| 1224 | Rifapentine | 61379-65-5 |
| 1225 | Aprepitant | 170729-80-3 |
| 1226 | Fludrocortisone (acetate) | 514-36-3 |
| 1227 | Losartan | 114798-26-4 |
| 1228 | Rotigotine (Hydrochloride) | 125572-93-2 |
| 1229 | Sulconazole (nitrate) | 82382-23-8 |
| 1230 | Amodiaquin (dihydrochloride dihydrate) | 6398-98-7 |
| 1231 | Losartan (potassium) | 124750-99-8 |
| 1232 | Isradipine | 75695-93-1 |
| 1233 | Homatropine (methylbromide) | 80-49-9 |
| 1234 | Pimavanserin tartrate | 706782-28-7 |
| 1235 | Lamivudine | 134678-17-4 |
| 1236 | Estradiol (benzoate) | 50-50-0 |
| 1237 | Menadione | 58-27-5 |
| 1238 | L-Glutamic acid monosodium salt | 142-47-2 |
| 1239 | Malathion | 121-75-5 |
| 1240 | Diphylline | 479-18-5 |
| 1241 | D-Pantothenic acid (hemicalcium salt) | 137-08-6 |
| 1242 | Bezafibrate | 41859-67-0 |
| 1243 | Enalaprilat (dihydrate) | 84680-54-6 |
| 1244 | Clotrimazole | 23593-75-1 |
| 1245 | Icotinib (Hydrochloride) | 1204313-51-8 |
| 1246 | Carglumic Acid | 1188-38-1 |
| 1247 | Procainamide (hydrochloride) | 614-39-1 |
| 1248 | Dacarbazine | 4342-03-4 |
| 1249 | Venlafaxine (hydrochloride) | 99300-78-4 |
| 1250 | Aniracetam | 72432-10-1 |
| 1251 | Flupirtine (Maleate) | 75507-68-5 |
| 1252 | Entacapone | 130929-57-6 |
| 1253 | Zanamivir | 139110-80-8 |
| 1254 | Amlexanox | 68302-57-8 |
| 1255 | Daptomycin | 103060-53-3 |
| 1256 | Triclosan | 3380-34-5 |
| 1257 | Argatroban (monohydrate) | 141396-28-3 |
| 1258 | Valpromide | 2430-27-5 |
| 1259 | Trimethoprim | 738-70-5 |
| 1260 | Amiloride (hydrochloride) | 2016-88-8 |
| 1261 | Clomipramine (hydrochloride) | 17321-77-6 |
| 1262 | Ledipasvir (acetone) | 1441674-54-9 |
| 1263 | (-)-Sparteine (sulfate pentahydrate) | 6160-12-9 |
| 1264 | Tolperisone (hydrochloride) | 3644-61-9 |
| 1265 | Adefovir dipivoxil | 142340-99-6 |
| 1266 | Latanoprost | 130209-82-4 |
| 1267 | Quinine | 130-95-0 |
| 1268 | Faropenem sodium | 122547-49-3 |
| 1269 | Estriol | 50-27-1 |
| 1270 | Osimertinib | 1421373-65-0 |
| 1271 | Sulfacetamide (Sodium) | 127-56-0 |
| 1272 | Pemetrexed (disodium hemipenta hydrate) | 357166-30-4 |
| 1273 | Flufenamic acid | 530-78-9 |
| 1274 | Mestranol | 72-33-3 |
| 1275 | Oseltamivir (phosphate) | 204255-11-8 |
| 1276 | Fosfluconazole | 194798-83-9 |
| 1277 | Trichlormethiazide | 133-67-5 |
| 1278 | Pheniramine (Maleate) | 132-20-7 |
| 1279 | Panobinostat | 404950-80-7 |
| 1280 | Fluvastatin (sodium) | 93957-55-2 |
| 1281 | Mefenamic acid | 61-68-7 |
| 1282 | Nicotinamide | 98-92-0 |
| 1283 | Imidapril (hydrochloride) | 89396-94-1 |
| 1284 | Venetoclax | 1257044-40-8 |
| 1285 | Cyproheptadine (hydrochloride) | 969-33-5 |
| 1286 | Capsaicin | 404-86-4 |
| 1287 | Fludarabine | 21679-14-1 |
| 1288 | Selexipag | 475086-01-2 |
| 1289 | Diethylcarbamazine (citrate) | 1642-54-2 |
| 1290 | Anastrozole | 120511-73-1 |
| 1291 | Bosentan | 147536-97-8 |
| 1292 | Cefuroxime (sodium) | 56238-63-2 |
| 1293 | Idramantone | 20098-14-0 |
| 1294 | Tebipenem pivoxil | 161715-24-8 |
| 1295 | Amorolfine (hydrochloride) | 78613-38-4 |
| 1296 | Talipexole dihydrochloride | 36085-73-1 |
| 1297 | Parecoxib (Sodium) | 198470-85-8 |
| 1298 | Fursultiamine | 804-30-8 |
| 1299 | Procyclidine (hydrochloride) | 1508-76-5 |
| 1300 | Dropropizine | 17692-31-8 |
| 1301 | Chlortetracycline (hydrochloride) | 64-72-2 |
| 1302 | Escitalopram (oxalate) | 219861-08-2 |
| 1303 | Thiamine nitrate | 532-43-4 |
| 1304 | Gemifloxacin (mesylate) | 210353-53-0 |
| 1305 | Micafungin (sodium) | 208538-73-2 |
| 1306 | Gliquidone | 33342-05-1 |
| 1307 | Sofalcone | 64506-49-6 |
| 1308 | S-(+)-Ketoprofen | 22161-81-5 |
| 1309 | Ribociclib succinate hydrate | 1374639-79-8 |
| 1310 | Protriptyline (hydrochloride) | 1225-55-4 |
| 1311 | Prednisone | 53-03-2 |
| 1312 | Carvedilol | 72956-09-3 |
| 1313 | Meropenem (trihydrate) | 119478-56-7 |
| 1314 | Valdecoxib | 181695-72-7 |
| 1315 | Pyridostigmine (bromide) | 101-26-8 |
| 1316 | Metaxalone | 1665-48-1 |
| 1317 | Griseofulvin | 126-07-8 |
| 1318 | Guanabenz (Acetate) | 23256-50-0 |
| 1319 | Valnemulin (Hydrochloride) | 133868-46-9 |
| 1320 | Sotalol (hydrochloride) | 959-24-0 |
| 1321 | Gabapentin enacarbil | 478296-72-9 |
| 1322 | Tinoridine hydrochloride | 25913-34-2 |
| 1323 | Ketoprofen | 22071-15-4 |
| 1324 | Cyclosporin A | 59865-13-3 |
| 1325 | Ilaprazole | 172152-36-2 |
| 1326 | Nicergoline | 27848-84-6 |
| 1327 | Bazedoxifene (acetate) | 198481-33-3 |
| 1328 | Metformin (hydrochloride) | 1115-70-4 |
| 1329 | Ozagrel | 82571-53-7 |
| 1330 | Acemetacin | 53164-05-9 |
| 1331 | Tropisetron | 89565-68-4 |
| 1332 | Prucalopride | 179474-81-8 |
| 1333 | Buspirone (hydrochloride) | 33386-08-2 |
| 1334 | Acyclovir | 59277-89-3 |
| 1335 | Teniposide | 29767-20-2 |
| 1336 | Mesna | 19767-45-4 |
| 1337 | Rucaparib (Camsylate) | 1859053-21-6 |
| 1338 | Regorafenib (Hydrochloride) | 835621-07-3 |
| 1339 | Trospium (chloride) | 10405-02-4 |
| 1340 | Vonoprazan | 881681-00-1 |
| 1341 | Telbivudine | 3424-98-4 |
| 1342 | Etomidate (hydrochloride) | 53188-20-8 |
| 1343 | Betaxolol (hydrochloride) | 63659-19-8 |
| 1344 | Otilonium (bromide) | 26095-59-0 |
| 1345 | Tofacitinib | 477600-75-2 |
| 1346 | Antazoline (hydrochloride) | 2508-72-7 |
| 1347 | Nalidixic acid | 389-08-2 |
| 1348 | Tolcapone | 134308-13-7 |
| 1349 | Mizoribine | 50924-49-7 |
| 1350 | Choline (chloride) | 67-48-1 |
| 1351 | Bicyclol | 118159-48-1 |
| 1352 | Bortezomib | 179324-69-7 |
| 1353 | Dextrose | 50-99-7 |
| 1354 | Fenbufen | 36330-85-5 |
| 1355 | Lodoxamide | 53882-12-5 |
| 1356 | Bromisoval | 496-67-3 |
| 1357 | Quinidine | 56-54-2 |
| 1358 | Tucidinostat | 1616493-44-7 |
| 1359 | Thiamphenicol | 15318-45-3 |
| 1360 | Felodipine | 72509-76-3 |
| 1361 | Bephenium (hydroxynaphthoate) | 3818-50-6 |
| 1362 | Pramocaine (hydrochloride) | 637-58-1 |
| 1363 | Sertraline (hydrochloride) | 79559-97-0 |
| 1364 | Rivastigmine (tartrate) | 129101-54-8 |
| 1365 | Dobutamine (hydrochloride) | 49745-95-1 |
| 1366 | Meglumine | 6284-40-8 |
| 1367 | Methylbenactyzium Bromide | 3166-62-9 |
| 1368 | Tolmetin (sodium dihydrate) | 64490-92-2 |
| 1369 | Amlodipine | 88150-42-9 |
| 1370 | Flucytosine | 2022-85-7 |
| 1371 | Doxepin (Hydrochloride) | 1229-29-4 |
| 1372 | Memantine (hydrochloride) | 41100-52-1 |
| 1373 | Nitrofurazone | 59-87-0 |
| 1374 | Hydroquinidine | 1435-55-8 |
| 1375 | Tolbutamide | 64-77-7 |
| 1376 | Raltitrexed | 112887-68-0 |
| 1377 | Indinavir (sulfate) | 157810-81-6 |
| 1378 | Dimetridazole | 551-92-8 |
| 1379 | Loxoprofen | 68767-14-6 |
| 1380 | Artemisinin | 63968-64-9 |
| 1381 | Valproic acid (sodium salt) | 1069-66-5 |
| 1382 | Methacholine (chloride) | 62-51-1 |
| 1383 | Troxipide | 30751-05-4 |
| 1384 | Mitotane | 53-19-0 |
| 1385 | Trimebutine (maleate) | 34140-59-5 |
| 1386 | Ticagrelor | 274693-27-5 |
| 1387 | Furosemide | 54-31-9 |
| 1388 | (S)-Timolol (Maleate) | 26921-17-5 |
| 1389 | Prednisolone (21-acetate) | 52-21-1 |
| 1390 | Molindone (hydrochloride) | 15622-65-8 |
| 1391 | Hydroxyfasudil | 105628-72-6 |
| 1392 | Fluocinonide | 356-12-7 |
| 1393 | Lenvatinib | 417716-92-8 |
| 1394 | Glibenclamide | 10238-21-8 |
| 1395 | Cefoperazone | 62893-19-0 |
| 1396 | Cilastatin | 82009-34-5 |
| 1397 | Ethambutol (dihydrochloride) | 1070-11-7 |
| 1398 | Triamcinolone (acetonide) | 76-25-5 |
| 1399 | Furazolidone | 67-45-8 |
| 1400 | Sulfalene | 152-47-6 |
| 1401 | Demeclocycline (hydrochloride) | 64-73-3 |
| 1402 | Mephenesin | 59-47-2 |
| 1403 | Troglitazone | 97322-87-7 |
| 1404 | Sivelestat (sodium tetrahydrate) | 201677-61-4 |
| 1405 | Deracoxib | 169590-41-4 |
| 1406 | Brexpiprazole | 913611-97-9 |
| 1407 | Nefopam (hydrochloride) | 23327-57-3 |
| 1408 | Ceftriaxone (sodium salt) | 74578-69-1 |
| 1409 | Moxisylyte (hydrochloride) | 964-52-3 |
| 1410 | Ixazomib citrate | 1239908-20-3 |
| 1411 | Albendazole | 54965-21-8 |
| 1412 | Amphotericin B | 1397-89-3 |
| 1413 | Cephalexin (monohydrate) | 23325-78-2 |
| 1414 | Stavudine | 3056-17-5 |
| 1415 | Mianserin (hydrochloride) | 21535-47-7 |
| 1416 | Apatinib | 1218779-75-9 |
| 1417 | Sulfanilamide | 63-74-1 |
| 1418 | D-Panthenol | 81-13-0 |
| 1419 | Olaparib | 763113-22-0 |
| 1420 | Tacrolimus (monohydrate) | 109581-93-3 |
| 1421 | Terpin (hydrate) | 2451-01-6 |
| 1422 | Fenoldopam (mesylate) | 67227-57-0 |
| 1423 | Darunavir (Ethanolate) | 635728-49-3 |
| 1424 | Tafamidis | 594839-88-0 |
| 1425 | Flunarizine (dihydrochloride) | 30484-77-6 |
| 1426 | Aceglutamide | 2490-97-3 |
| 1427 | Cefaclor | 53994-73-3 |
| 1428 | Dipyridamole | 58-32-2 |
| 1429 | Artemotil | 75887-54-6 |
| 1430 | Sivelestat | 127373-66-4 |
| 1431 | Acitretin | 55079-83-9 |
| 1432 | Mycophenolate Mofetil | 128794-94-5 |
| 1433 | Diclofenac (Sodium) | 15307-79-6 |
| 1434 | Sitagliptin | 486460-32-6 |
| 1435 | Suplatast (Tosilate) | 94055-76-2 |
| 1436 | Pargyline (hydrochloride) | 306-07-0 |
| 1437 | Lopinavir | 192725-17-0 |
| 1438 | Noscapine | 128-62-1 |
| 1439 | Iopamidol | 60166-93-0 |
| 1440 | 2-Ethoxybenzamide | 938-73-8 |
| 1441 | Vandetanib | 443913-73-3 |
| 1442 | Topiramate | 97240-79-4 |
| 1443 | Ranolazine (dihydrochloride) | 95635-56-6 |
| 1444 | Flucloxacillin sodium | 1847-24-1 |
| 1445 | Cyproterone (acetate) | 427-51-0 |
| 1446 | Sodium 4-aminosalicylate dihydrate | 6018-19-5 |
| 1447 | Bumetanide | 28395-03-1 |
| 1448 | Bivalirudin (TFA) | 1191386-55-6 |
| 1449 | (-)-Huperzine A | 102518-79-6 |
| 1450 | Acipimox | 51037-30-0 |
| 1451 | Nepafenac | 78281-72-8 |
| 1452 | Loratadine | 79794-75-5 |
| 1453 | Propyphenazone | 479-92-5 |
| 1454 | Pipobroman | 54-91-1 |
| 1455 | Moexipril (hydrochloride) | 82586-52-5 |
| 1456 | Pivmecillinam (hydrochloride) | 32887-03-9 |
| 1457 | Valganciclovir (hydrochloride) | 175865-59-5 |
| 1458 | Avibactam (sodium hydrate) |  |
| 1459 | Oxybutynin | 5633-20-5 |
| 1460 | Zaltoprofen | 74711-43-6 |
| 1461 | Iopromide | 73334-07-3 |
| 1462 | Trimipramine (maleate) | 521-78-8 |
| 1463 | Proparacaine (Hydrochloride) | 5875-06-9 |
| 1464 | Citalopram (hydrobromide) | 59729-32-7 |
| 1465 | Regorafenib | 755037-03-7 |
| 1466 | Amlodipine (maleate) | 88150-47-4 |
| 1467 | Bepotastine (Beslilate) | 190786-44-8 |
| 1468 | Voglibose | 83480-29-9 |
| 1469 | Almitrine mesylate | 29608-49-9 |
| 1470 | Tropicamide | 1508-75-4 |
| 1471 | Tenofovir alafenamide fumarate | 379270-38-9 |
| 1472 | Fluvoxamine (maleate) | 61718-82-9 |
| 1473 | Drospirenone | 67392-87-4 |
| 1474 | Sulfisomidin | 515-64-0 |
| 1475 | Idoxuridine | 54-42-2 |
| 1476 | Loperamide (hydrochloride) | 34552-83-5 |
| 1477 | Sofosbuvir | 1190307-88-0 |
| 1478 | Teprenone | 6809-52-5 |
| 1479 | Auranofin | 34031-32-8 |
| 1480 | Fasudil (Hydrochloride) | 105628-07-7 |
| 1481 | Vecuronium (bromide) | 50700-72-6 |
| 1482 | Desonide | 638-94-8 |
| 1483 | Rifampicin | 13292-46-1 |
| 1484 | Ornipressin | 3397-23-7 |
| 1485 | Ademetionine (disulfate tosylate) | 97540-22-2 |
| 1486 | Zafirlukast | 107753-78-6 |
| 1487 | Tenofovir (Disoproxil) | 201341-05-1 |
| 1488 | Gemcitabine | 95058-81-4 |
| 1489 | Dexrazoxane hydrochloride | 149003-01-0 |
| 1490 | Mefloquine (hydrochloride) | 51773-92-3 |
| 1491 | Prucalopride (succinate) | 179474-85-2 |
| 1492 | Monensin sodium salt | 22373-78-0 |
| 1493 | Sparfloxacin | 110871-86-8 |
| 1494 | Dirithromycin | 62013-04-1 |
| 1495 | Miridesap | 224624-80-0 |
| 1496 | Telithromycin | 191114-48-4 |
| 1497 | Flunisolide | 3385-03-3 |
| 1498 | Mitomycin C | 50-07-7 |
| 1499 | Bleomycin (sulfate) | 9041-93-4 |
| 1500 | 10-Undecenoic acid (zinc salt) | 557-08-4 |
| 1501 | Ioversol | 87771-40-2 |
| 1502 | Nitazoxanide | 55981-09-4 |
| 1503 | Colistin (sulfate) | 1264-72-8 |
| 1504 | Pirenzepine (dihydrochloride) | 29868-97-1 |
| 1505 | Deoxycholic acid sodium salt | 302-95-4 |
| 1506 | Tranexamic acid | 1197-18-8 |
| 1507 | Azaphen (dihydrochloride monohydrate) | 63302-99-8 |
| 1508 | Citicoline | 987-78-0 |
| 1509 | Donepezil (Hydrochloride) | 120011-70-3 |
| 1510 | Sisomicin (sulfate) | 53179-09-2 |
| 1511 | Heparin (sodium salt) | 9041-08-1 |
| 1512 | 4-(Aminomethyl)benzoic acid | 56-91-7 |
| 1513 | Aminophylline | 317-34-0 |
| 1514 | L-Arginine (hydrochloride) | 1119-34-2 |
| 1515 | Tenofovir | 147127-20-6 |
| 1516 | Kanamycin (sulfate) | 25389-94-0 |
| 1517 | Ozagrel sodium | 189224-26-8 |
| 1518 | L-SelenoMethionine | 3211-76-5 |
| 1519 | Gabapentin (hydrochloride) | 60142-95-2 |
| 1520 | Vigabatrin | 68506-86-5 |
| 1521 | Lecithin | 8002-43-5 |
| 1522 | Biapenem | 120410-24-4 |
| 1523 | Dexamethasone phosphate disodium | 2392-39-4 |
| 1524 | Gabapentin | 60142-96-3 |
| 1525 | Netilmicin (sulfate) | 56391-57-2 |
| 1526 | Salbutamol (hemisulfate) | 51022-70-9 |
| 1527 | Nedaplatin | 95734-82-0 |
| 1528 | Streptomycin (sulfate) | 3810-74-0 |
| 1529 | Sodium Fluoride | 7681-49-4 |
| 1530 | Neomycin (sulfate) | 1405-10-3 |
| 1531 | Paromomycin (sulfate) | 1263-89-4 |
| 1532 | Gadobutrol | 770691-21-9 |
| 1533 | Fosfomycin (calcium) | 26016-98-8 |
| 1534 | Bekanamycin | 4696-76-8 |
| 1535 | Diquafosol (tetrasodium) | 211427-08-6 |
| 1536 | Ribostamycin (sulfate) | 53797-35-6 |
| 1537 | Chloroquine (diphosphate) | 50-63-5 |
| 1538 | Tobramycin | 32986-56-4 |
| 1539 | Gluconate (Calcium) | 299-28-5 |
| 1540 | Alendronate (sodium hydrate) | 121268-17-5 |
| 1541 | Amifostine | 20537-88-6 |
| 1542 | (2S)-2-Amino-3-methyl-3-sulfanylbutanoic acid | 52-67-5 |
| 1543 | Hydralazine (hydrochloride) | 304-20-1 |
| 1544 | L-Arginine | 74-79-3 |
| 1545 | Peramivir (trihydrate) | 1041434-82-5 |
| 1546 | Amikacin (sulfate) | 39831-55-5 |
| 1547 | Levoleucovorin (Calcium) | 80433-71-2 |
| 1548 | Tranylcypromine (hemisulfate) | 13492-01-8 |
| 1549 | Hydroxychloroquine sulfate | 747-36-4 |
| 1550 | Mangafodipir (trisodium) | 140678-14-4 |
| 1551 | Plerixafor (octahydrochloride) | 155148-31-5 |
| 1552 | Capreomycin (sulfate) | 1405-37-4 |
| 1553 | Gastrodenol | 57644-54-9 |
| 1554 | DL-Glutamine | 6899-04-3 |
| 1555 | Lisinopril (dihydrate) | 83915-83-7 |
| 1556 | Selenomethionine | 1464-42-2 |
| 1557 | DL-Arginine | 7200-25-1 |
| 1558 | Miltefosine | 58066-85-6 |
| 1559 | L-Glutamine | 56-85-9 |
| 1560 | Bremelanotide (Acetate) | 1607799-13-2 |
| 1561 | Maraviroc | 376348-65-1 |
| 1562 | Diclofenac (diethylamine) | 78213-16-8 |
| 1563 | Benzthiazide | 91-33-8 |
| 1564 | Dalbavancin | 171500-79-1 |
| 1565 | Ibrutinib Racemate | 936563-87-0 |
| 1566 | Pexidartinib hydrochloride | 2040295-03-0 |
| 1567 | Allopregnanolone | 516-54-1 |
| 1568 | Adenine | 73-24-5 |
| 1569 | Teriparatide | 52232-67-4 |
| 1570 | Quinagolide (hydrochloride) | 94424-50-7 |
| 1571 | Urea | 57-13-6 |
| 1572 | Reboxetine (mesylate) | 98769-84-7 |
| 1573 | Omarigliptin | 1226781-44-7 |
| 1574 | Bosutinib | 380843-75-4 |
| 1575 | Forodesine (hydrochloride) | 284490-13-7 |
| 1576 | Aprotinin | 9087-70-1 |
| 1577 | Risperidone | 106266-06-2 |
| 1578 | Blonanserin | 132810-10-7 |
| 1579 | Talc | 14807-96-6 |
| 1580 | Fumaric acid | 110-17-8 |
| 1581 | Imrecoxib | 395683-14-4 |
| 1582 | Idarubicin (hydrochloride) | 57852-57-0 |
| 1583 | Penbutolol (sulfate) | 38363-32-5 |
| 1584 | Glycine | 56-40-6 |
| 1585 | Norepinephrine | 51-41-2 |
| 1586 | Bimatoprost | 155206-00-1 |
| 1587 | Ripasudil | 887375-67-9 |
| 1588 | Cyclic somatostatin | 38916-34-6 |
| 1589 | Varenicline (Tartrate) | 375815-87-5 |
| 1590 | Clindamycin (phosphate) | 24729-96-2 |
| 1591 | Berberine (chloride) | 633-65-8 |
| 1592 | Abemaciclib (methanesulfonate) | 1231930-82-7 |
| 1593 | Benzocaine | 94-09-7 |
| 1594 | Erismodegib diphosphate | 1218778-77-8 |
| 1595 | Ingenol | 30220-46-3 |
| 1596 | Neratinib | 698387-09-6 |
| 1597 | Norfloxacin | 70458-96-7 |
| 1598 | Vesnarinone | 81840-15-5 |
| 1599 | Prednisone acetate | 125-10-0 |
| 1600 | Merbromin | 129-16-8 |
| 1601 | Talazoparib | 1207456-01-6 |
| 1602 | Minocycline (hydrochloride) | 13614-98-7 |
| 1603 | Flubendazole | 31430-15-6 |
| 1604 | Triamcinolone hexacetonide | 5611-51-8 |
| 1605 | Erdafitinib | 1346242-81-6 |
| 1606 | Acalabrutinib | 1420477-60-6 |
| 1607 | Bictegravir | 1611493-60-7 |
| 1608 | Cefoperazone (sodium salt) | 62893-20-3 |
| 1609 | Tannic acid | 1401-55-4 |
| 1610 | Dydrogesterone | 152-62-5 |
| 1611 | Ziprasidone | 146939-27-7 |
| 1612 | Lusutrombopag | 1110766-97-6 |
| 1613 | Escin | 6805-41-0 |
| 1614 | L-Lactic acid | 79-33-4 |
| 1615 | Amprenavir | 161814-49-9 |
| 1616 | Duvelisib | 1201438-56-3 |
| 1617 | (R)-Baclofen | 69308-37-8 |
| 1618 | Nystatin | 1400-61-9 |
| 1619 | Edoxaban | 480449-70-5 |
| 1620 | i-Inositol | 87-89-8 |
| 1621 | Dextrorotation nimorazole phosphate ester | 1124347-33-6 |
| 1622 | L-Thyroxine (sodium salt pentahydrate) | 6106-07-6 |
| 1623 | Tafamidis meglumine | 951395-08-7 |
| 1624 | Biperiden (Hydrochloride) | 1235-82-1 |
| 1625 | Maltitol | 585-88-6 |
| 1626 | Pyrvinium pamoate | 3546-41-6 |
| 1627 | Sucrose | 57-50-1 |
| 1628 | Carboxin | 5234-68-4 |
| 1629 | Abiraterone | 154229-19-3 |
| 1630 | Clioquinol | 130-26-7 |
| 1631 | Fostamatinib Disodium | 1025687-58-4 |
| 1632 | Finafloxacin | 209342-40-5 |
| 1633 | L-Tryptophan | 73-22-3 |
| 1634 | Upadacitinib | 1310726-60-3 |
| 1635 | Peficitinib | 944118-01-8 |
| 1636 | Erismodegib | 956697-53-3 |
| 1637 | Glecaprevir | 1365970-03-1 |
| 1638 | Taurodeoxychloic Acid (sodium hydrate) | 110026-03-4 |
| 1639 | Ranolazine | 95635-55-5 |
| 1640 | Baloxavir marboxil | 1985606-14-1 |
| 1641 | Esaxerenone | 1632006-28-0 |
| 1642 | Larotrectinib | 1223403-58-4 |
| 1643 | Moxidectin | 113507-06-5 |
| 1644 | Travoprost | 157283-68-6 |
| 1645 | Norvancomycin (hydrochloride) | 213997-73-0 |
| 1646 | Cobimetinib (racemate) | 934662-91-6 |
| 1647 | Alpelisib | 1217486-61-7 |
| 1648 | Apalutamide | 956104-40-8 |
| 1649 | Lodoxamide (tromethamine) | 63610-09-3 |
| 1650 | Dapiprazole (hydrochloride) | 72822-13-0 |
| 1651 | Iloprost | 78919-13-8 |
| 1652 | Celecoxib | 169590-42-5 |
| 1653 | Santonin | 481-06-1 |
| 1654 | Emetine (dihydrochloride) | 316-42-7 |
| 1655 | Gallamine Triethiodide | 65-29-2 |
| 1656 | Nalfurafine (hydrochloride) | 152658-17-8 |
| 1657 | Lodenafil | 139755-85-4 |
| 1658 | Asenapine | 65576-45-6 |
| 1659 | Axitinib | 319460-85-0 |
| 1660 | Lubiprostone | 136790-76-6 |
| 1661 | Pexidartinib | 1029044-16-3 |
| 1662 | Sulfabenzamide | 127-71-9 |
| 1663 | Suprofen | 40828-46-4 |
| 1664 | Arterolane | 664338-39-0 |
| 1665 | Siponimod | 1230487-00-9 |
| 1666 | Crisaborole | 906673-24-3 |
| 1667 | Chloroxine | 773-76-2 |
| 1668 | Ebastine | 90729-43-4 |
| 1669 | Guanethidine (sulfate) | 645-43-2 |
| 1670 | Grazoprevir potassium salt | 1206524-86-8 |
| 1671 | Delafloxacin (meglumine) | 352458-37-8 |
| 1672 | Furosemide (sodium) | 41733-55-5 |
| 1673 | Aminohippurate (sodium) | 94-16-6 |
| 1674 | Phenprocoumon | 435-97-2 |
| 1675 | Fabomotizole (hydrochloride) | 173352-39-1 |
| 1676 | Elbasvir | 1370468-36-2 |
| 1677 | Anisindione | 117-37-3 |
| 1678 | Aclacinomycin A hydrochloride | 75443-99-1 |
| 1679 | β-Carotene | 7235-40-7 |
| 1680 | Midostaurin | 120685-11-2 |
| 1681 | Methylcobalamin | 13422-55-4 |
| 1682 | Opicapone | 923287-50-7 |
| 1683 | Nifurtimox | 23256-30-6 |
| 1684 | Fluorescein | 2321-07-5 |
| 1685 | Eravacycline (dihydrochloride) | 1334714-66-7 |
| 1686 | Dibucaine | 85-79-0 |
| 1687 | Lanatoside C | 17575-22-3 |
| 1688 | Niraparib hydrochloride | 1038915-64-8 |
| 1689 | Metixene hydrochloride hydrate | 7081-40-5 |
| 1690 | Limaprost | 74397-12-9 |
| 1691 | Thiabendazole | 148-79-8 |
| 1692 | Nelfinavir | 159989-64-7 |
| 1693 | Laropiprant | 571170-77-9 |
| 1694 | Dacomitinib | 1110813-31-4 |
| 1695 | Azilsartan (medoxomil) | 863031-21-4 |
| 1696 | Deoxycorticosterone (acetate) | 56-47-3 |
| 1697 | Binimetinib | 606143-89-9 |
| 1698 | Favipiravir | 259793-96-9 |
| 1699 | Tipranavir | 174484-41-4 |
| 1700 | Eliglustat | 491833-29-5 |
| 1701 | Grazoprevir | 1350514-68-9 |
| 1702 | Phenylbutazone | 50-33-9 |
| 1703 | Sulfamerazine | 127-79-7 |
| 1704 | Gonadorelin (acetate) | 34973-08-5 |
| 1705 | Biotin | 58-85-5 |
| 1706 | Benfluorex hydrochloride | 23642-66-2 |
| 1707 | Monocrotaline | 315-22-0 |
| 1708 | Linezolid | 165800-03-3 |
| 1709 | Danoprevir | 850876-88-9 |
| 1710 | Camphor | 76-22-2 |
| 1711 | Decamethonium (Bromide) | 541-22-0 |
| 1712 | Atropine methyl bromide | 2870-71-5 |
| 1713 | Degarelix | 214766-78-6 |
| 1714 | Sacubitril hemicalcium salt | 1369773-39-6 |
| 1715 | AZD7545 | 252017-04-2 |
| 1716 | Cysteamine | 60-23-1 |
| 1717 | Caffeic acid | 331-39-5 |
| 1718 | Tezacaftor | 1152311-62-0 |
| 1719 | Yohimbine (Hydrochloride) | 65-19-0 |
| 1720 | Morinidazole | 92478-27-8 |
| 1721 | Cytisinicline | 485-35-8 |
| 1722 | Pirmenol (hydrochloride) | 61477-94-9 |
| 1723 | Piperonyl butoxide | 51-03-6 |
| 1724 | Taurine | 107-35-7 |
| 1725 | Ixabepilone | 219989-84-1 |
| 1726 | Urapidil | 34661-75-1 |
| 1727 | Hydrocortisone 17-butyrate | 13609-67-1 |
| 1728 | Sulfadoxine | 2447-57-6 |
| 1729 | Pretomanid | 187235-37-6 |
| 1730 | Inosine pranobex | 36703-88-5 |
| 1731 | Lofexidine | 31036-80-3 |
| 1732 | Pyridoxine (hydrochloride) | 58-56-0 |
| 1733 | Erythromycin Ethylsuccinate | 1264-62-6 |
| 1734 | Paritaprevir | 1216941-48-8 |
| 1735 | Triclabendazole | 68786-66-3 |
| 1736 | Evans Blue | 314-13-6 |
| 1737 | Tafenoquine (Succinate) | 106635-81-8 |
| 1738 | Ceftaroline fosamil | 400827-46-5 |
| 1739 | Tigecycline (tetramesylate) |  |
| 1740 | Temoporfin | 122341-38-2 |
| 1741 | Larotrectinib sulfate | 1223405-08-0 |
| 1742 | Pasireotide (ditrifluoroacetate) |  |
| 1743 | Deferasirox (Fe3+ chelate) | 554435-83-5 |
| 1744 | Mirodenafil (dihydrochloride) | 862189-96-6 |
| 1745 | Treprostinil (sodium) | 289480-64-4 |
| 1746 | Cholic acid | 81-25-4 |
| 1747 | Bentiromide | 37106-97-1 |
| 1748 | Docosahexaenoic Acid | 6217-54-5 |
| 1749 | Glycerol phenylbutyrate | 611168-24-2 |
| 1750 | (+)-α-Tocopherol | 59-02-9 |
| 1751 | Helicid | 80154-34-3 |
| 1752 | Relugolix | 737789-87-6 |
| 1753 | Hydrocortisone buteprate | 72590-77-3 |
| 1754 | Lorlatinib | 1454846-35-5 |
| 1755 | Tolterodine (tartrate) | 124937-52-6 |
| 1756 | Ertugliflozin L-pyroglutamic acid | 1210344-83-4 |
| 1757 | (Z)-Capsaicin | 25775-90-0 |
| 1758 | Zalcitabine | 7481-89-2 |
| 1759 | VAL-083 | 23261-20-3 |
| 1760 | Benorilate | 5003-48-5 |
| 1761 | Morinidazole (R enantiomer) | 898230-59-6 |
| 1762 | Catechin | 154-23-4 |
| 1763 | Ivosidenib | 1448347-49-6 |
| 1764 | L-Cysteine | 52-90-4 |
| 1765 | Roxadustat | 808118-40-3 |
| 1766 | Toloxatone | 29218-27-7 |
| 1767 | Argipressin | 113-79-1 |
| 1768 | Atrasentan (hydrochloride) | 195733-43-8 |
| 1769 | Ethynyl Estradiol | 57-63-6 |
| 1770 | Ethoxzolamide | 452-35-7 |
| 1771 | (-)-Menthol | 2216-51-5 |
| 1772 | Gadoxetate (Disodium) | 135326-22-6 |
| 1773 | Sodium citrate dihydrate | 6132-04-3 |
| 1774 | Varenicline | 249296-44-4 |
| 1775 | DHEA | 53-43-0 |
| 1776 | Tolterodine | 124937-51-5 |
| 1777 | Dibucaine (hydrochloride) | 61-12-1 |
| 1778 | Xylitol | 87-99-0 |
| 1779 | Demecarium Bromide | 56-94-0 |
| 1780 | Ceftizoxime | 68401-81-0 |
| 1781 | Resveratrol | 501-36-0 |
| 1782 | Umeclidinium (bromide) | 869113-09-7 |
| 1783 | Eletriptan (hydrobromide) | 177834-92-3 |
| 1784 | Etripamil | 1593673-23-4 |
| 1785 | Glasdegib | 1095173-27-5 |
| 1786 | Hydrocortisone cypionate | 508-99-6 |
| 1787 | Asunaprevir | 630420-16-5 |
| 1788 | Icatibant | 130308-48-4 |
| 1789 | Tricaprilin | 538-23-8 |
| 1790 | Eperisone (Hydrochloride) | 56839-43-1 |
| 1791 | Tiamulin (fumarate) | 55297-96-6 |
| 1792 | Terbinafine hydrochloride | 78628-80-5 |
| 1793 | Ertugliflozin | 1210344-57-2 |
| 1794 | Fosamprenavir | 226700-79-4 |
| 1795 | Fesoterodine (fumarate) | 286930-03-8 |
| 1796 | Guaiacol | 90-05-1 |
| 1797 | Vernakalant (Hydrochloride) | 748810-28-8 |
| 1798 | Fructose | 7660-25-5 |
| 1799 | Benznidazol | 22994-85-0 |
| 1800 | Thioridazine (hydrochloride) | 130-61-0 |
| 1801 | Medetomidine (hydrochloride) | 86347-15-1 |
| 1802 | Varenicline (Hydrochloride) | 230615-23-3 |
| 1803 | Pinaverium bromide | 53251-94-8 |
| 1804 | Sacubitril | 149709-62-6 |
| 1805 | Dixyrazine | 2470-73-7 |
| 1806 | Vilanterol (trifenatate) | 503070-58-4 |
| 1807 | Elagolix sodium | 832720-36-2 |
| 1808 | Naloxegol (oxalate) | 1354744-91-4 |
| 1809 | Calcium dobesilate | 20123-80-2 |
| 1810 | Fluralaner | 864731-61-3 |
| 1811 | L-5-Hydroxytryptophan | 4350-09-8 |
| 1812 | 5-Aminolevulinic acid (hydrochloride) | 5451-09-2 |
| 1813 | Revefenacin | 864750-70-9 |
| 1814 | Methyl Salicylate | 119-36-8 |
| 1815 | Prasugrel (hydrochloride) | 389574-19-0 |
