# Supplementary material for: Idarubicin combats abiraterone and enzalutamide resistance in prostate cells via targeting XPA protein
Source: Cell Death Dis. 2022 Dec 12;13(12):1034. doi: 10.1038/s41419-022-05490-5 (PMC9744908; doi:10.1038/s41419-022-05490-5)
Supplement: Supplementary file 14 — Table s2 [file 41419_2022_5490_MOESM14_ESM.docx]

Table. S2 Hits drugs in primary screen

| No | Drugs Name | Rep1 | Rep2 |
| --- | --- | --- | --- |
| 1 | Otilonium (bromide) | 96.25453 | 94.1297 |
| 2 | Idarubicin (hydrochloride) | 91.23618 | 72.44526 |
| 3 | Auranofin | 50.32962 | 70.08197 |
| 4 | Sitafloxacin (hydrate) | 62.24832 | 59.92385 |
| 5 | Pyrvinium pamoate | 59.42987 | 55.03965 |
| 6 | Erdafitinib | 57.66534 | 54.98721 |
