# Supplementary material for: Idarubicin combats abiraterone and enzalutamide resistance in prostate cells via targeting XPA protein
Source: Cell Death Dis. 2022 Dec 12;13(12):1034. doi: 10.1038/s41419-022-05490-5 (PMC9744908; doi:10.1038/s41419-022-05490-5)
Supplement: Supplementary file 17 — Table s5 [file 41419_2022_5490_MOESM17_ESM.docx]

Tab. S5 Genes involving in the development of Abi resistance and decreased by IDA treatment

| Protein | Protein Name | Gene | LFC | score | p.low | p.high | p.twosided | FDR |
| --- | --- | --- | --- | --- | --- | --- | --- | --- |
| P23025 | DNA repair protein complementing XP-A cells | XPA | -5.444 | 4.3614 | 6.46E-06 | 0.99999 | 1.29E-05 | 4.12E-05 |
| Q15723 | ETS-related transcription factor Elf-2 | ELF2 | -5.2441 | 4.1141 | 1.94E-05 | 0.99998 | 3.89E-05 | 0.000113 |
| Q13868 | Exosome complex component RRP4 | EXOSC2 | -5.163 | 4.0184 | 2.93E-05 | 0.99997 | 5.86E-05 | 0.000165 |
| P20248 | Cyclin-A2 | CCNA2 | -5.1314 | 3.9817 | 3.42E-05 | 0.99997 | 6.84E-05 | 0.00019 |
| P40616 | ADP-ribosylation factor-like protein 1 | ARL1 | -5.1294 | 3.9794 | 3.45E-05 | 0.99997 | 6.91E-05 | 0.000192 |
| Q8NDX5 | Polyhomeotic-like protein 3 | PHC3 | -5.11 | 3.957 | 3.79E-05 | 0.99996 | 7.59E-05 | 0.000209 |
| P61966 | AP-1 complex subunit sigma-1A | AP1S1 | -5.0949 | 3.9399 | 4.08E-05 | 0.99996 | 8.15E-05 | 0.000223 |
| Q96F25 | UDP-N-acetylglucosamine transferase subunit ALG14 homolog | ALG14 | -5.0602 | 3.9005 | 4.8E-05 | 0.99995 | 9.6E-05 | 0.000259 |
| Q5TZA2 | Rootletin | CROCC | -5.0596 | 3.8999 | 4.81E-05 | 0.99995 | 9.63E-05 | 0.00026 |
| Q86WW8 | Cytochrome c oxidase assembly factor 5 | COA5 | -5.0393 | 3.8769 | 5.29E-05 | 0.99995 | 0.000106 | 0.000283 |
| Q92871 | Phosphomannomutase 1 | PMM1 | -5.0387 | 3.8763 | 5.3E-05 | 0.99995 | 0.000106 | 0.000284 |
